# Genome-Wide Identification and Characterization of ABC Transporters in Nine Rosaceae Species Identifying *MdABCG28* as a Possible Cytokinin Transporter linked to Dwarfing

**DOI:** 10.3390/ijms20225783

**Published:** 2019-11-17

**Authors:** Yi Feng, Qiran Sun, Guifen Zhang, Ting Wu, Xinzhong Zhang, Xuefeng Xu, Zhenhai Han, Yi Wang

**Affiliations:** 1 Department of Pomology, College of Horticulture, China Agricultural University, Beijing 100193, China; fengyi@cau.edu.cn (Y.F.); b20193170856@cau.edu.cn (Q.S.); b20163170699@cau.edu.cn (G.Z.); wuting@cau.edu.cn (T.W.); zhangxinzhong999@126.com (X.Z.); xuefengx@cau.edu.cn (X.X.); rschan@cau.edu.cn (Z.H.); 2 State Key Laboratory of Plant Physiology and Biochemistry, College of Biological Sciences, China Agricultural University, Beijing 100193, China

**Keywords:** ABC transporter, cytokinin transport, Rosaceae, apple, dwarfing, bioinformatics

## Abstract

ATP-binding cassette (ABC) transporters constitute a large, diverse, and ubiquitous superfamily that is involved in a broad range of processes. The completion of genome sequencing provides an opportunity to understand the phylogenetic history of the ABC transporter superfamily among Rosaceae species. This study identified a total of 1323 ABC transporter genes from nine Rosaceae genomes: 191 from *Malus domestica*, 174 from *Pyrus communis*, 138 from *Prunus persica*, 118 from *Prunus avium*, 141 from *Prunus dulcis*, 122 from *Fragaria vesca*, 98 from *Rubus occidentalis*, 162 from *Prunus mume*, and 179 from *Rosa chinensis*. Their chemical characterization, phylogenetic analysis, chromosomal localization, gene structure, gene duplication, and tissue-specific expression were studied. Their subcellular localization, transmembrane structures, and protein motifs were predicted. All the ABC transporter genes were grouped into eight subfamilies on the basis of their phylogenetic relationships and structural features. Furthermore, cis-element and expression analysis of 10 potential phytohormone transporters in MdABCG subfamily genes were also performed. Loss of the W-box in the promoter region of *MdABCG28* was found to reduce the gene expression level and was linked to the dwarfing phenotype in apple rootstocks. *MdABCG28* overexpression promoted shoot growth of *atabcg14* mutants in Arabidopsis.

## 1. Introduction

The ATP-binding cassette (ABC) transporter superfamily comprises both membrane-bound transporters and soluble proteins. The majority of all identified ABC transporter genes encode membrane-bound proteins that directly participate in the transport of a wide range of molecules across membranes [1]. ABC transporters are defined as having ABCs, which are also known as nucleotide-binding domains (NBDs), and contain several highly conserved motifs, including the Walker A and B sequences, the ABC signature motif, the H loop, and the Q loop [2]. In addition to NBDs, ABC transporters also contain transmembrane domains (TMDs), each consisting of several hydrophobic α-helices. The core unit contains a functional ABC transporter, comprising four domains: two NBDs and two TMDs. Both NBDs synergistically bind and hydrolyze ATP, and provide the driving force for transport; moreover, the TMDs are involved in substrate recognition and translocation across the lipid bilayer [3]. Although all NBDs share both a common evolutionary origin and mechanism, the sequences of these coupled TMDs may differ considerably, which can potentially exhibit important mechanistic differences [4]. The subfamily G of the ABC transporter (ABCG) is markedly expanded in plants, with 131 members in Arabidopsis. The expansion of this ABCG transporter appears to be related to the functional diversification of specific taxa.

Phytohormones coordinate both plant growth and development in response to endogenous cues and environmental changes. It has been proposed that the transportability of most phytohormones is particularly important, which has been suggested to be compatible with their role as information carriers across plant tissues and organs [5]. During the past few years, different transporter families have been reported to mediate the phytohormone transport. These studies indicated that ABC transporters play a central role in both the export and import of abscisic acid (ABA), cytokinins, and auxigenic compounds.

AtABCG25 is a half-size ABC transporter that has been identified in ABA sensitivity screening. The germination of *atabcg25* mutant seeds was more strongly inhibited by externally applied ABA than the corresponding wild-type. *AtABCG25* is localized in the plasma membrane and is mainly expressed in the vasculature of roots and fruits, with only very weak expression in leaves [6]. In addition, the expression can be induced by exogenous ABA treatment in the shoot vasculature. Therefore, *AtABCG25* expression overlaps strongly with ABA biosynthetic enzymes. Transport experiments, using vesicles isolated from insect cells expressing AtABCG25, also identified AtABCG25 as a high-affinity ABA exporter with strong substrate preference for the natural ABA. Further studies showed that overexpression of *AtABCG25* resulted in elevated leaf temperature, which indicates a decrease in transpiration, consistent with the role of AtABCG25 as an ABA efflux pump and for the de novo synthesis of ABA.

Screening of a large number of ABCG knockout plants showed that the stomatal movement of *atabcg40* was impaired after treatment with exogenous ABA. Using thermal imagery, it has been demonstrated that the leaves of *atabcg40* plants heated up slowly in response to ABA exposure, indicating a higher rate of transpiration in response to both ABA exposure and osmotic stress [7]. The AtABCG40 expression in yeast and tobacco cells showed that AtABCG40 exhibits a high-affinity and stereospecific ABA uptake activity. This was also confirmed by the much slower up-regulation of ABA-responsive genes in *atabcg40* plants compared with the corresponding wild-type when exposed to exogenous ABA.

To identify further potential ABA transporters, thermal imagery showed that *atabcg22* transpired more than the corresponding wild-type. *AtABCG22* is mainly expressed in guard cells and is localized in the plasma membrane in the aerial parts of Arabidopsis [8]. It has been suggested that AtABCG22 has an ABA-related phenotype, but does not act directly as an ABA transporter. Consistent with this suggestion, no AtABCG22-mediated ABA transport activity was observed during heterologous expression in various systems.

Using genome-wide association mapping, the AtABCG16 transporter was identified as a candidate gene involved in the defense against *Pseudomonas syringae* DC3000 (*Pst* DC3000, a virulence factor that leads to the up-regulation of the ABA signaling pathway) [9]. This ABC transporter is highly up-regulated in response to ABA exposure, and knockout plants were not only more susceptible to ABA, but also to *Pst* DC3000. These data suggest that AtABCG16 is an ABA exporter. However, to corroborate this speculation, it must be demonstrated how the exact pathogen- sensitivity increasing mechanism is associated with the increased sensitivity to exogenously applied ABA.

AtABCG14 has been reported to be involved in cytokinin transport, which is highly co-expressed as a gene with cytokinin biosynthesis. The phenotype of the *atabcg14* knockout plants could be rescued with *trans*-zeatin (*t*Z-type) cytokinin spray, indicating the ability of AtABCG14 to mediate root to shoot translocation of *t*Z-type cytokinin. Determination of the cytokinin contents in roots and shoots showed that the *t*Z-type cytokinin level was strongly decreased in shoots, whereas it was increased in the roots of *atabcg14* mutant plants. In contrast, the *N*^6^-(Δ^2^-isopentenyl) adenine (iP-type) cytokinin level was increased both in *atabcg14* roots and shoots [10,11]. The main reason for this is that iP-type cytokinin is transported via the phloem to the root, where it is converted to *t*Z-type cytokinin and redistributed to the shoot via AtABCG14. AtABCG14 is a root-localized transporter that is responsible for the allocation of *t*Z-type cytokinin to the shoot.

AtABCG37 is a pleiotropic drug-resistant transporter that transports a range of synthetic auxinic compounds as well as the endogenous auxin precursor indole-3-butyric acid (IBA); however, it does not transport free indole-3-acetic acid (IAA) [12]. AtABCG37 acts redundantly at the outermost root plasma membranes and removes IBA from the cells, unlike established IAA transporters of the PINFORMED (PIN) and ABCB families.

Rosaceae form a large branch with high economic value in Archichlamydeae, and has been divided into three subfamilies: Maloideae, Prunoideae, and Rosoideae. Although the ABC transporter superfamily has already been reported in a number of horticultural plants, such as *Vitis vinifera* [13], *Ananas comosus* [14], *Solanum lycopersicum* [15], *Brassica rapa* [16], and *Brassica napus* [17], no comprehensive study of ABC transporters in Rosaceae has been reported to date. This study conducted bioinformatic research on nine published Rosaceae genomes toward the genome-wide identification and characterization of members of the ABC transporter superfamily. Two members of Maloideae (*Malus domestica* and *Pyrus communis*), four members of Prunoideae (*Prunus persica*, *Prunus avium*, *Prunus dulcis*, and *Prunus mume*), and three members of Rosoideae (*Fragaria vesca*, *Rubus occidentalis*, and *Rosa chinensis*) were investigated. Expression analysis of 10 potential phytohormone transporters in MdABCG subfamily genes and validation of *MdABCG28* function were conducted to provide a theoretical basis for further study of the long-distance transport of phytohormones in Rosaceae.

## 2. Results

### 2.1. The ABC Transporter Superfamilies in Rosaceae

To identify and study ABC transporter superfamilies within the Rosaceae genome, Arabidopsis members were used as query in basic local alignment search tool of protein (BLASTp) to search for homologues. A total of 1323 ABC transporter genes were identified from nine Rosaceae species: 191 from the apple genome (*M. domestica*), 174 from the European pear genome (*P. communis*), 138 from the peach genome (*P. persica*), 118 from the sweet cherry genome (*P. avium*), 141 from the almond genome (*P. dulcis*), 122 from the woodland strawberry genome (*F. vesca*), 98 from the black raspberry genome (*R. occidentalis*), 162 from the mei genome (*P. mume*), and 179 from the rose genome (*R. chinensis*).

**Figure 1:**
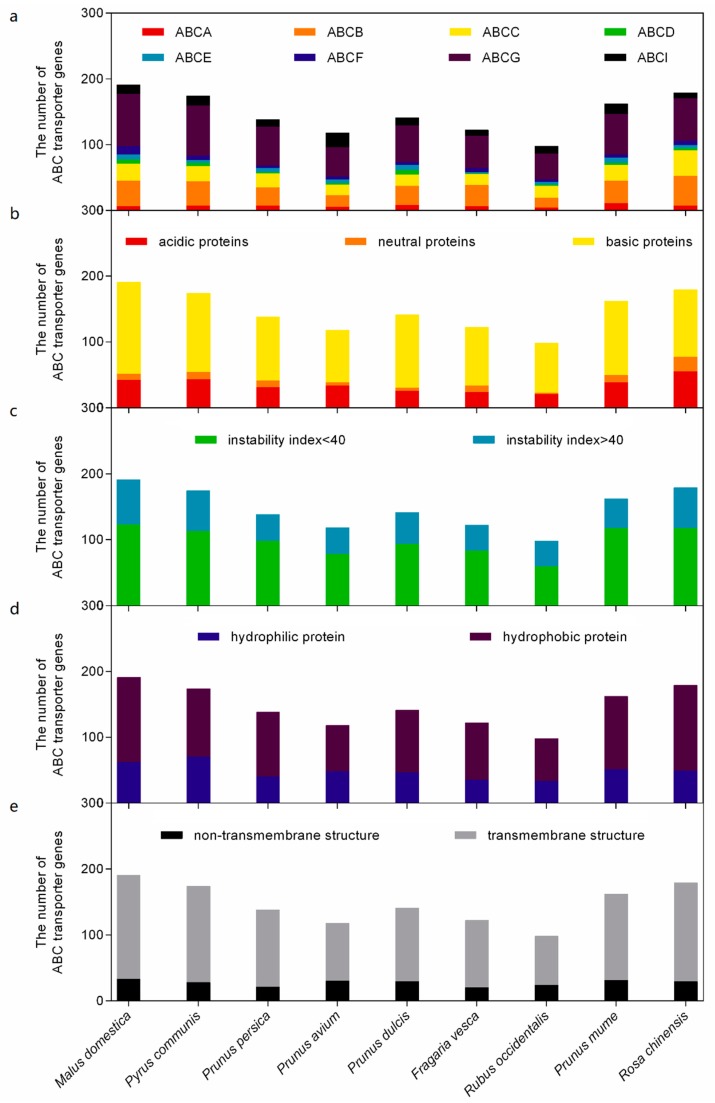
Numbers of ATP-binding cassette (ABC) transporter family members for each classification in Rosaceae: (**a**) subfamily; (**b**) isoelectric point; (**c**) instability index; (**d**) grand average of hydropathicity; (**e**) transmembrane structure.

Phylogenetic analyses were performed to investigate the evolutionary relationships of all ABC transporter genes of these nine Rosaceae species (Supplementary Figure S1). The ABCG proteins constitute the largest subfamily among these nine genomes, whereas the ABCB and ABCC subfamilies rank second and third, respectively (Figure 1a). The chemical characterization, including protein length, molecular weight, isoelectric point, total number of negatively/positively charged residues, molecular formula, instability index, aliphatic index, and grand average of hydropathicity, were preformed. Moreover, subcellular localization and transmembrane structures were predicted for each member of the ABC proteins (Supplementary Table S1).

The protein lengths of these ABC transporter genes ranged from 157 to 4352, and the molecular weights ranged from 17.4 to 485.5 kD. The 1323 proteins were classified into 310 acidic proteins, 85 neutral proteins, and 928 basic proteins, according to their isoelectric points (Figure 1b). Of the 1323 proteins, 442 had an instability index above 40, indicating that they were instable (Figure 1c). Furthermore, 434 of the grand average of hydropathicity were negative, identifying them as hydrophilic proteins (Figure 1d). Most proteins used for the prediction for subcellular localization were consistent with their Arabidopsis homologues, and 1078 of these featured a number of transmembrane structures (Figure 1e).

### 2.2. Chromosomal Localization, Motif Array, and Gene Structure of ABC Transporter Families

These ABC transporter genes of nine Rosaceae species were classified on the basis of their location on chromosomes to investigate their genomic distribution. The five genomes of *M. domestica*, *P. persica*, *P. dulcis*, *F. vesca*, and *R. chinensis*, have 17, 8, 8, 7, and 7 chromosomes, respectively. The ABC transporter genes are distributed on all chromosomes of each genome, whereas the other four genomes of *P. communis*, *P. avium*, *R. occidentalis*, and *P. mume* were not assembled to the chromosome level but only to the scaffold level (Supplementary Figures S2a and S3).

MEME analysis identified a total of 20 putative conserved motifs in the 1323 ABC transporter genes, which were named motif 1 to motif 20 (Supplementary Table S2), the lengths of which ranged from 15 to 50 amino acids (Supplementary Figures S2b and S4).

The exon–intron structure of the ABC transporter genes in nine Rosaceae species have been analyzed to determine their structural diversity. Among the 1323 ABC transporter genes, 59 members did not have introns, including six in *M. domestica*, five in *P. communis*, six in *P. persica*, four in *P. avium*, six in *P. dulcis*, six in *F. vesca*, nine in *R. occidentalis*, nine in *P. mume*, and eight in *R. chinensis* (Supplementary Figures S2c and S4).

### 2.3. Tandem Duplication and Segmental Duplication of the ABC Transporter Families

Tandem duplications and segmental duplications formed the main gene duplication events. A total of 132 pairs of ABC transporter genes were distributed close to each other, including 13 in *M. domestica*, 6 in *P. communis*, 13 in *P. persica*, 8 in *P. avium*, 29 in *P. dulcis*, 6 in *F. vesca*, 2 in *R. occidentalis*, 34 in *P. mume*, and 21 in *R. chinensis*. These had similar coding lengths, exon-intron structures, and motif arrays, indicating that both genes may be tandem duplicated genes (Supplementary Figures S2d and S5).

In addition, 319 pairs of segmental duplicated ABC transporter genes were found in the nine Rosaceae genomes, including 97 in *M. domestica*, 97 in *P. communis*, 13 in *P. persica*, 39 in *P. avium*, 32 in *P. dulcis*, 9 in *F. vesca*, 10 in *R. occidentalis*, 5 in *P. mume*, and 17 in *R. chinensis*. These had identity indexes >80% when BLASTp in pairs were performed among the 1323 ABC transporter proteins (Supplementary Figures S2e and S5).

### 2.4. Identification and Characterization of Potential Phytohormone Transporters in MdABCG Subfamily Genes

**Figure 2:**
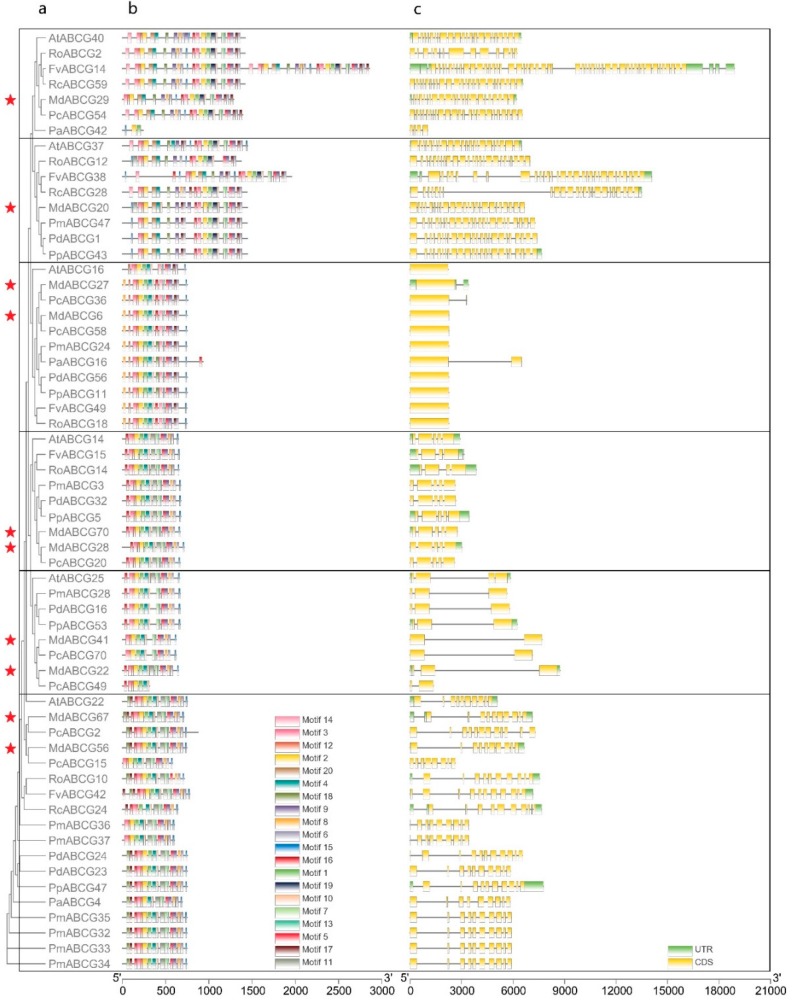
Phylogenetic trees, motif analysis, and gene structures of potential phytohormone transporters in ABCG subfamily genes of nine Rosaceae species. (**a**) The phylogenetic tree was constructed on the basis of the full-length sequences of known phytohormone transporter genes in Arabidopsis and homologous genes in nine Rosaceae species using the neighbor-joining (NJ) method and 1000 replications for bootstrap analysis in MEGA 6.0. Red stars indicate apple homologues. (**b**) Motif analyses. Twenty motifs are indicated with different colored boxes. Logos of each motif are shown in Table 1. All motifs were identified via online analysis with the package MEME Suite 5.0.2. (**c**) Gene structures. Exons are represented by yellow boxes. Black lines connecting two exons represent introns. Green boxes represent untranslated regions (UTRs). Gene structure maps were drawn with TBtools.

To further investigate potential phytohormone transporters in MdABCG subfamily genes and their evolution in Rosaceae, a total of 55 homologous genes were identified in these nine Rosaceae species using the known phytohormone transporter genes in Arabidopsis (Figure 2). Most of these known Arabidopsis phytohormone transporter genes have two apple homologs, but have no homologs in other species (Figure 2a). The motif array and exon–intron structure in homologous genes were quite consistent (Figure 2b,c). These motifs were also strongly conserved in ABCG subfamily genes of these nine Rosaceae species (Table 1).

**Figure 3:**
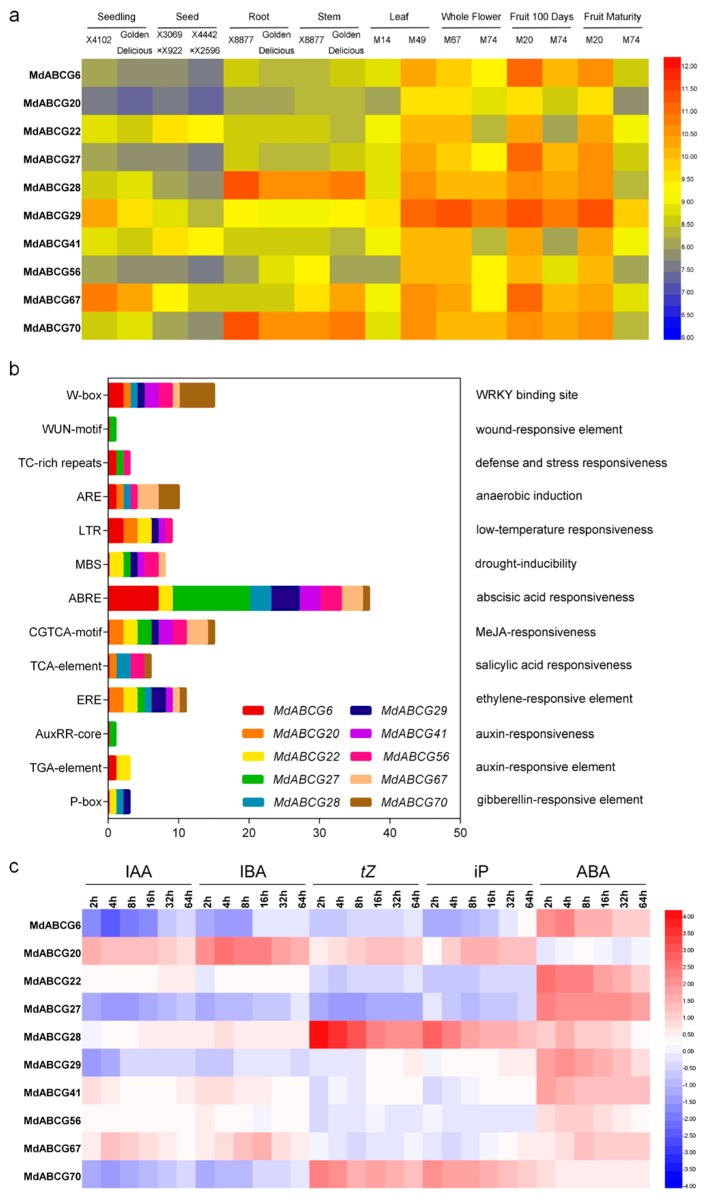
Expression profiles of 10 potential phytohormone transporters in MdABCG subfamily genes. (**a**) Tissue expression profiles obtained from ArrayExpress data displaying diverse expression levels of 10 potential phytohormone transporters in MdABCG subfamily genes in different tissues. Relative transcript levels based on ArrayExpress data are presented as heat maps from blue to red, reflecting relative signal values. (**b**) Cis-elements analysis. These cis-elements responded to environmental stress and phytohormone signal in the sense strands of 10 MdABCG subfamily gene promoters. The *x*-axis indicates the number of each cis-element. (**c**) Inducible expression profiles of 10 MdABCG subfamily genes under indole-3-acetic acid (IAA) (1 mM), indole-3-butyric acid (IBA) (1 mM), *trans*-zeatin (*t*Z) (1 mM), *N*^6^-(Δ^2^-isopentenyl) adenine (iP) (1 mM), or abscisic acid (ABA) (1 mM) in apple callus. The color scale represents the log2 transformed gene relative expression compared with controls.

The biological roles of 10 potential phytohormone transporters in MdABCG subfamily genes were further examined in different developmental stages and tissues on the basis of a set of microarray data obtained from the ArrayExpress database (E-EGOD-42873). A total of 7 tissue or organ types from 10 apple varieties or hybrids were analyzed (Figure 3a). Although the expression of these 10 MdABCG subfamily genes varied among different samples, most of the MdABCG subfamily genes were minimally expressed in seedlings and seeds. It is worth noting that *MdABCG28* and *MdABCG70*, as homologues of the cytokinin transporter *AtABCG14*, were highly expressed in roots.

The diversity of the distribution of cis-elements in promoters may lead to differences in gene functions and regulatory patterns. Cis-elements were identified in the promoter regions of 10 potential phytohormone transporters in MdABCG subfamily genes. These were involved in the response to environmental stress or phytohormone signal, including six stress-related elements and seven phytohormone-related elements. All these genes contained five to eight cis-elements related to the stress or phytohormone response (Figure 3b).

One of the indicators of a phytohormone transporter was induced. To further confirm whether the expressions of these 10 MdABCG subfamily genes were phytohormone-induced, apple callus were used and their expression patterns in response to phytohormone treatments were analyzed (Figure 3c). The underlying reason was that undifferentiated tissue is conducive to a focus on exogenous induction rather than tissue specificity. *AtABCG37* has been reported as a transporter of IBA (rather than a free IAA transporter), which removes IBA from cells, unlike established IAA transporters from the PIN and ABCB families. *MdABCG20*, as a homologue of *AtABCG37*, was strongly induced by IBA from 2 to 64 h, indicating that *MdABCG20* has the function to transport excessive IBA. For cytokinins, *MdABCG28* and *MdABCG70*, as homologues of the cytokinin transporter *AtABCG14*, were strongly induced by *t*Z and iP. In addition, as homologues of ABA transporters, five genes can respond to ABA, including *MdABCG22* and *MdABCG41* as homologues of *AtABCG25*, *MdABCG29* as a homologue of *AtABCG40*, and *MdABCG6* and *MdABCG27* as homologues of *AtABCG16*, whereas *MdABCG56* and *MdABCG67* as homologues of *AtABCG22* are not induced by ABA.

### 2.5. Natural Variation in the Promoter Region of MdABCG28 Reduces the Gene Expression Level and Links to the Dwarfing Phenotype in Apple Rootstocks

**Figure 4:**
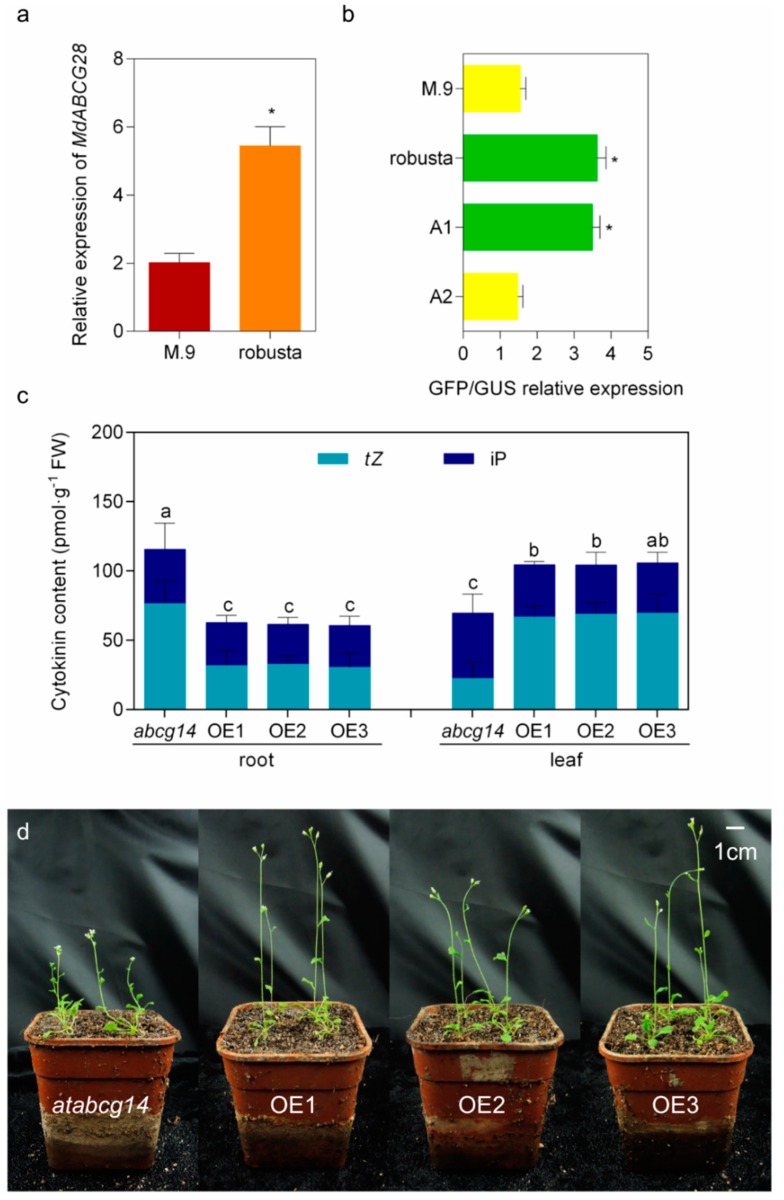
Functional analysis of *MdABCG28* and its variant promoter. (**a**) Relative expression of *MdABCG28* in roots of apple rootstocks. (**b**) Transient GFP expression assay in Arabidopsis protoplasts testing the effects of the 17 bp deletion variant in the *MdABCG28* promoter. The 2 kb promoter region of *MdABCG28* was amplified and cloned into the pCambia1302 vector (GFP tag) by inserting it into the EcoRI/KpnI site. The M.9 construct contained a 17 bp deletion sequence (-GAACCGTC**TTGAC**ATGT-) in the region from 1186 to 1202 bp with a W-box (-TTGAC-) compared with the robusta construct. The A1 construct was generated on the basis of the M.9 construct with a 17 bp deletion sequence inserted back. The A2 construct was generated on the basis of robusta construct with W-box mutated (-GAACCGTC**TTCAC**ATGT-). The pCAMBIA1301 vector (GUS tag) was used as internal control. The panel shows the GFP expression levels relative to GUS (*n* = 10). (**c**) Cytokinin contents and (**d**) phenotypes of the *atabcg14* mutant and its overexpressed lines of *MdABCG28*. The full-length cDNA of *MdABCG28* was amplified and cloned into the pCambia1300 vector by inserting it into the EcoRI/KpnI site. The resulting recombinant vector was inserted into *Agrobacterium tumefaciens* LBA4404 cells to transform *atabcg14* mutant plants. Homozygous T3 transgenic lines were used. Biological replicates were performed in triplicate. Error bars represent the standard error. Asterisks indicate *p*-values below 0.05, obtained using a two-tailed Student’s *t*-test. Different letters indicate *p*-values below 0.05, obtained using Duncan’s test.

Since *MdABCG28* was up-regulated more than 10-fold within a short time (up to 16 times within 2 h in apple callus) when induced by *t*Z (Figure 3c), its expression in apple rootstocks has aroused further interest. The expression of *MdABCG28* in the roots of a dwarfing rootstock M.9 was significantly lower than in the roots of the vigorous rootstock robusta (Figure 4a). To understand the difference in *MdABCG28* expression, the *MdABCG28* upstream promoter sequence was cloned. A heterozygous 17 bp deletion sequence (-GAACCGTCTTGACATGT-) was identified from 1186 to 1202 bp upstream of the start codon on the *MdABCG28* promoter in the M.9 rootstock, but not in the robusta rootstock. Further analysis of this 17 bp deleted sequence identified a W-box (-TTGAC-) on it, which is a WRKY binding site. To test whether this 17 bp deletion variant affected *MdABCG28* expression, the promoter activity of this promoter variant was tested using GFP/GUS double-label transient expression in Arabidopsis protoplasts. Expression constructs containing M.9-type promoter with 17 bp deletion sequence inserted back (A1 construct) and robusta-type promoter with W-box mutated (A2 construct) were generated and compared with the expression levels of both M.9 and robusta full promoter sequences. The results showed that the activity of the M.9-type promoter was significantly lower than that of the robusta-type promoter. When the 17 bp deletion sequence was inserted back into the M.9-type promoter, the activity was enhanced. When the W-box was mutated on the robusta-type promoter, the activity was decreased (Figure 4b). This indicated that the loss of W-box led to a decrease of *MdABCG28* promoter activity.

To verify the function of *MdABCG28*, its full-length complementary DNA (cDNA) was overexpressed into *atabcg14* mutants in Arabidopsis. Three T3 transgenic lines (OE1, OE2, and OE3) with higher basal were selected for the further analysis of their function in cytokinin transport (Supplementary Figure S6). With the transfer of cytokinin from roots to shoots (Figure 4c), transgenic plants showed a phenotype that promoted shoot growth (Figure 4d). This suggests that *MdABCG28* as a possible cytokinin transporter and the dwarfing phenotype of *atabcg14* has been restored. Accordingly, low expression of *MdABCG28* may be linked to the dwarfing phenotype in the M.9 rootstock.

## 3. Discussion

To adapt to a variable environment, plants have evolved complex movement systems to establish steep concentration gradients of solutes across cellular membranes. Among these systems, ABC transporter proteins play an important role, and are involved in various biological processes and ubiquitous in all living organisms [18]. Numerous ABC transporter genes have been identified in different horticultural plant species, including 135 members in *V. vinifera* [13], 100 members in *A. comosus* [14], 154 members in *S. lycopersicum* [15], 179 members in *B. rapa* [16], and 314 members in *B. napus* [17]. This study comprehensively investigated ABC transporter genes of nine Rosaceae species, using whole genome-wide identification, phylogenetic analysis, chromosomal localization, structural investigation, gene duplication, and tissue-specific expression. A total of 1323 ABC transporter genes were identified, with numbers ranging from a minimum of 98 to a maximum of 191 among the nine Rosaceae genomes (Figure 1a).

Gene duplication events are significant for the rapid expansion and evolution of gene families. Furthermore, tandem duplications and segmental duplications have been identified as major duplication modes for gene family expansion [19]. This study showed that 345 and 319 genes were involved in tandem duplication and segmental duplication, accounting for 26.1% and 24.1% of all ABC transporter members, respectively (Supplementary Figure S2d,e). Notably, *M. domestica* and *P. communis* (both of which belong to Maloideae) made a major contribution to the number of segmental duplications, and accounted for more than 60% among nine Rosaceae genomes (Supplementary Figure S2e). The reason is that whole-genome duplication (WGD) occurred during the process of apple and pear domestication, but not in the other seven investigated species [20,21].

Over the past few years, many families of transporters have been shown to mediate phytohormone transport. These studies showed that ABC transporters play a central role in exporting and importing ABA, cytokinins, and auxigenic compounds [5]. In this study, 10 MdABCG genes were induced by phytohormone application in the apple callus (Figure 3c), which were potential phytohormone transporters. Genetic and functional screens identified four ABC transporter family members implicated in ABA transport in Arabidopsis [22]. This study identified seven of their homologues in the apple genome (Figure 2a), which were verified to be induced by ABA (Figure 3c). Undoubtedly, these transporters contributed to the success of apple plants in colonizing broad areas of land, as they allowed seeds to travel great distances from the mother plant and colonize new habitats. AtABCG14 has been identified as an efflux transporter, essential for the root-to-shoot translocation of *t*Z-type cytokinin [10,11]. Consistently, as its homologues *MdABCG28* and *MdABCG70* were highly expressed in roots (Figure 3a), and could be verified to be induced not only by *t*Z-type cytokinin, but also by iP-type cytokinin (Figure 3c). The likely reason is that both types can be converted into each other. *AtABCG37* has been reported to transport a range of auxinic compounds (including IBA), but not free IAA [12]. *MdABCG20* has been identified as its homologue and has been verified to be induced by IBA (Figure 3c). However, IBA may not be a primary transport substrate for *AtABCG37*, as its protein was found to transport other substrates [22].

It has previously been shown that root cytokinin synthesis was weak in the M.9 rootstock compared with the robusta rootstock [23]. Here, the expression of *MdABCG28* in roots of the M.9 rootstock was lower than that in roots of the robusta rootstock (Figure 4a). This means that the dual factor reduced cytokinin transport from root to shoot in M.9 rootstocks. Cytokinins are signaling molecules that facilitate communication between below-ground and above-ground organs. When conditions are favorable for growth, the root *t*Z-type cytokinin is translocated to the shoot through the xylem to stimulate shoot growth [24]. However, natural variation changes this situation. The loss of W-box found on the *MdABCG28* promoter in M.9 rootstock was found to reduce the expression of *MdABCG28* (Figure 4b), thus inhibiting the root-to-shoot transport of *t*Z-type cytokinin. Although it cannot be fully determined whether *MdABCG28* is directly associated with dwarf traits, overexpression lines of *MdABCG28* promote the shoot growth of *atabcg14* mutants (Figure 4c,d). In summary, the obtained results suggest that the loss of W-box on the *MdABCG28* promoter was one reason for the inhibition of stem elongation of the M.9 rootstock. In addition, the WRKY transcription factor has also been reported to regulate stem elongation [25,26]. Natural variation has been reported to provide a significant mechanism for evolutionary changes and phenotypic variations in apple rootstocks [27,28]. However, the relationship between the 17 bp deletion variant and the dwarfing phenotype in apple rootstocks requires further study.

In conclusion, a total of 1323 ABC transporter genes were identified from nine Rosaceae genomes in this study. A total of 10 MdABCG subfamily genes were identified to be phytohormone-induced. A loss of W-box on the *MdABCG28* promoter was found to decrease the gene expression level and could be linked to the dwarfing phenotype in apple rootstocks.

## 4. Materials and Methods

### 4.1. Database Searches and Identification of ABC Transporter Superfamilies in Rosaceae

The protein sequences of the Arabidopsis ABC transporter superfamilies were obtained from The Arabidopsis Information Resource database (http://www.arabidopsis.org/) and were used as query in BLASTp to search for homologues in the Genome Database for Rosaceae (http://www.rosaceae.org). The following genomes were included: *Malus × domestica* GDDH13 Whole Genome v1.1 [29], *Pyrus communis* Genome v1.0 [30], *Prunus persica* Genome v2.0.a1 [31], *Prunus avium* Genome v1.0.a1 [32], *Prunus dulcis* Texas Genome v2.0 [33], *Fragaria vesca* Genome v4.0.a1 [34], *Rubus occidentalis* Whole Genome Assembly v1.0.a1 [35], *Prunus mume* Genome v1.0 [36], and *Rosa chinensis* Genome v1.0 [37]. According to all candidates, amino acid sequences of the ABC transporter domain (PF00005) were identified through the Pfam protein families database (http://pfam.xfam.org/) [38]. The ProtParam tool in the ExPASy database (https://web.expasy.org/protparam/) [39] was used to analyze the chemical characterization and amino acid sequence composition of each member. The WoLF PSORT tool (https://wolfpsort.hgc.jp/) and TMHMM Server v.2.0 program (http://www.cbs.dtu.dk/services/TMHMM-2.0/) were used to predict subcellular localizations and transmembrane structures, respectively. All members of each genome were named according to chromosomal localizations.

### 4.2. Phylogenetic Analysis and Chromosome Localization

All ABC transporter gene sequences were aligned using the MAFFT algorithm on the MAFFT version 7 alignment server (https://mafft.cbrc.jp/alignment/server/) [40]. Phylogenetic trees were estimated with the MEGA 6.0 program using the neighbor-joining (NJ) method and implementing 1000 replications for the bootstrap analysis [41]. All ABC transporter gene annotations and their chromosomal locations were obtained from the GDR website. The exact location of genes on chromosomes was identified by using TBtools [42].

### 4.3. Motifs Analysis and Gene Structures

All protein motifs were recognized using the online analysis package MEME Suite 5.0.2 (http://meme-suite.org/tools/meme) [43]. The repetition was set to any number with an optimal width of 6–200 residues and the maximum number of motifs was set to 20. The exon/intron organizations were obtained by applying the method for the comparison sequence coding for aminoacids in protein (CDS) in exon position and gene length using TBtools [42], on the basis of the full-length genome sequence and the corresponding coding sequences.

### 4.4. Tandem Duplication and Segmental Duplication Analysis

Using the phylogenetic analysis approach, gene duplications were first estimated by manually verifying each subgroup. Then, an all-against-all BLASTp in pairs were performed in each genome. Duplicated gene pairs derived from tandem and segmental duplications were further identified on the basis of the method described in the Plant Genome Duplication Database (PGDD, http://chibba.agtec.uga.edu/duplication) [44]. The physical location of a gene on the chromosome was used to identify the tandem duplication. Genes with an adjacent homologous gene on the same chromosome and no more than one intervening gene were considered to be tandemly duplicated. Visual circos of the segmental duplication for each genome was performed using TBtools [42].

### 4.5. Putative Promoter Cis-Element Analysis

The putative promoter sequence, 2 kb of genomic sequence upstream of the translation start site of each potential phytohormone transporter in MdABCG subfamily genes, was extracted from the *Malus × domestica* GDDH13 Whole Genome v1.1 according to the general feature format file. Cis-elements were identified using the PlantCARE online program (http://bioinformatics.psb.ugent.be/webtools/plantcare/html) [45], and elements for stress responsiveness and phytohormone responsiveness were counted.

### 4.6. Transient Expression Assays of Promoter Activity

Fusion products of the *MdABCG28* promoters were generated using overlap PCR technology [46]. The pCambia1302 vector with GFP tag was intended to be used as an expression vector and was simultaneously transferred with the GUS tagged pCAMBIA1301 vector into Arabidopsis protoplasts as internal control. The promoter activity was represented by the GFP/GUS value. Primers with restriction sites used for vector construction are listed in Supplementary Table S3. Protoplasts were isolated from fully expanded healthy leaves of four-week-old Arabidopsis plants using a modified version of a previously published protocol [47]. Protoplasts were transformed with polyethylene glycol for 16 h, as previously described [48]. The protoplasts were then harvested for RNA extraction and GFP expression analysis.

### 4.7. Growth Conditions and Treatments of Apple Callus

For experiments involving expression patterns of 10 potential phytohormone transporters in MdABCG subfamily genes, *Malus domestica* Borkh. ‘Orin’ callus were cultured using a published procedure [49]. Callus were induced and subcultured every 20 days, and then transferred to a culture dish containing Murashige and Skoog (MS) medium supplemented with 1 mM IAA, 1 mM IBA, 1 mM *t*Z, 1 mM iP, or 1 mM ABA, separately. Callus in dishes were collected, according to treatment, either 2, 4, 8, 16, 32, or 64 h after treatment. For RNA extraction and determination of gene expression, data were expressed as means ± standard errors of three independent experiments performed in triplicate.

### 4.8. Generation of Transgenic Plants Using the atabcg14 Mutant

Arabidopsis ecotype Columbia was used as the wild type. The transfer DNA (T-DNA) insertion lines SK_15918 were obtained from the Arabidopsis Biological Resource Center. Homozygous seeds were sown on culture dishes containing half-strength MS medium. Seedlings with two true leaves were then transplanted and grown at 22 °C in a growth chamber under a long day regime (16 h light and 8 h dark). To construct the plasmid for plant transformation, the full-length cDNA of *MdABCG28* was isolated and cloned into the pCambia1300 vector to generate an overexpressing construct. Primers with restriction sites used for vector construction are listed in Supplementary Table S3. The resulting recombinant vector was inserted into *Agrobacterium tumefaciens* LBA4404 cells, which were then used to transform *atabcg14* mutant plants according to a floral dip protocol [50]. Positive transgenic plants were identified through RT-PCR screening. Transgenic lines were selected with 30 mg/L hygromycin. Homozygous T3 generation plants were used for further analysis.

### 4.9. RNA Isolation and RT-PCR

Total RNA was prepared using the Plant RNA Reagent (Invitrogen, Carlsbad, CA, USA). Superscript III First Strand cDNA Synthesis Kit (Invitrogen, USA) was used to synthesize first-strand cDNA. SYBR premix Ex Taq (TaKaRa, Shiga, Japan) was used to perform quantitative RT-PCR analysis in the ABI 7500 Real-time PCR system (Applied Biosystems, Foster City, CA, USA). The following cycles were used: 30 s at 95 °C, followed by 40 cycles of 5 s at 95 °C, 30 s at 60 °C, and then entering the melt curve stage [51]. Primers for quantitative RT-PCR are listed in Supplementary Table S4. *β-ACTIN* served as an internal reference to estimate relative expression levels.

### 4.10. Cytokinins Extraction and Quantification

Plant materials were homogenized in liquid nitrogen and placed in an extraction mixture consisting of methanol/water/formic acid. The supernatants were evaporated in a vacuum concentrator (Alpha RVC, Christ, Osterode, Germany), and were then applied to a mixed mode reversed phase-cation exchange SPE column (Oasis-MCX, Waters, Milford, MA, USA), as previously described [52]. The cytokinin fraction was sequentially eluted, evaporated, and finally dissolved in 5% MeOH. An ultra-performance liquid chromatography (1290, Agilent, Pal Alto, CA, USA) coupled to a hybrid triple quadrupole/linear ion trapmass spectrometer (4500 Q TRAP, AB SCIEX, Waltham, MA, USA) was used to analyze each aliquot.

## Figures and Tables

**Table 1:** Motifs information of potential phytohormone transporters in ABCG subfamily genes of nine Rosaceae species.

Motif	Logo	Sequence	*E-*Value	Sites	Width
1	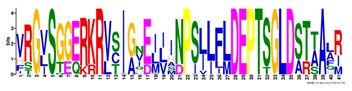	VRGVSGGERKRVSIGNEJIINPSJLFLDEPTSGLDSTTALR	4.9 × 10^−1822^	60	41
2	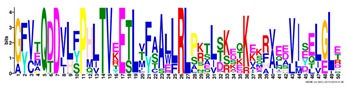	GFVTQDDVLFPHLTVEETLVFAALLRLPKTLSKEQKEKRVEEVISELGLE	1.1 × 10^−1875^	60	50
3	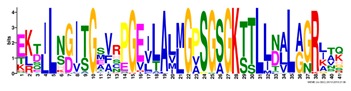	EKTJLNGITGSVRPGEILALLGPSGSGKTTLLBALAGRLTQ	1.2 × 10^−1524^	60	41
4	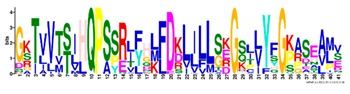	GKTVVTSIHQPSSRLFHLFDKLJLLSKGSLLYFGKASEAMV	1.5 × 10^−1397^	60	41
5	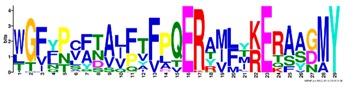	WGFYPCFTALFTFPQERAMLTKERAAGMY	8.3 × 10^−1056^	59	29
6	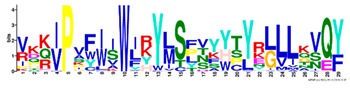	VKKIPVFIIWIRYLSFVYYTYRLLLKVQY	3.2 × 10^−924^	58	29
7	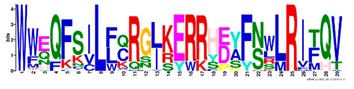	WWZQFSILFQRGJKERRHEYFNWLRITQV	3.9 × 10^−814^	41	29
8	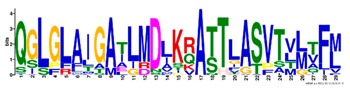	QGLGLAIGATLMDLKRATTLASVTVLTFM	4.9 × 10^−674^	46	29
9	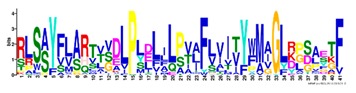	RLSAYFLARTVVDLPLDLILPVAFLVITYWMAGLRPSAETF	2.3 × 10^−1088^	58	41
10	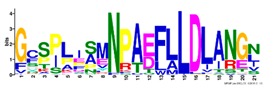	GCSPLISMNPAEFLLDLANGN	5.6 × 10^−528^	54	21
11	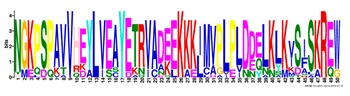	NGKPSPAVVHEYLVEAYETRVADEEKKKJMVPLPLDDELKLKVSISKREW	4.1 × 10^−705^	21	50
12	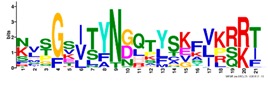	KVSGSITYNGQTYSKFVKRRT	2.1 × 10^−485^	59	21
13	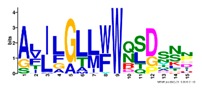	ALJLGLLWWQSDSNN	1.9 × 10^−350^	58	15
14	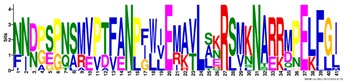	NNBPSPNSMVPTFANPFWIEMAVLAKRSMKNARRMPELFGI	3.9 × 10^−318^	15	41
15	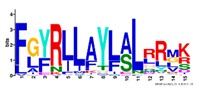	FGYRLLAYLALRRMK	5.3 × 10^−276^	54	15
16	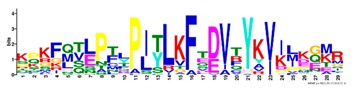	KPKFQTLPTLPJTLKFTDVTYKVILKGMR	1.8 × 10^−520^	48	29
17	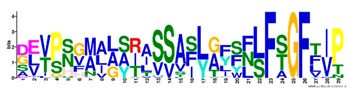	DEVPSGMALSRASSASLAFSFLFSGFTIP	4.9 × 10^−498^	40	29
18	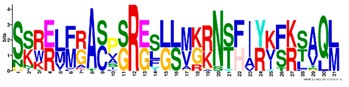	SSRELFRASPSRESLLMKRNSFIYKFKSAQL	1.0 × 10^−390^	26	31
19	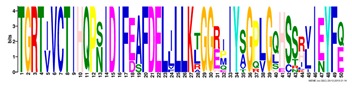	TGRTIVCTIHQPNIDIFEAFDELJLLKTGGRIIYSGPLGQHSSRVIEYFZ	2.9 × 10^−479^	14	50
20	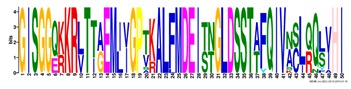	GISGGQKKRLTTAEMJIGPTKALFMDEITNGLDSSTAFQIVNSLQQLVHI	5.0 × 10^−469^	14	50

## Supplementary Materials

Supplementary materials can be found at https://www.mdpi.com/1422-0067/20/22/5783/s1.
