# Supplementary material for: Genome-Wide Identification and Characterization of ABC Transporters in Nine Rosaceae Species Identifying MdABCG28 as a Possible Cytokinin Transporter linked to Dwarfing
Source: Int J Mol Sci. 2019 Nov 17;20(22):5783. doi: 10.3390/ijms20225783 (PMC6887749; doi:10.3390/ijms20225783)

Supplemental Figure 3. Chromosomal location of ABC transporter genes in nine Rosaceae species

Supplemental Figure 3-1 Chromosomal location of ABC transporter genes in *Malus domestica*


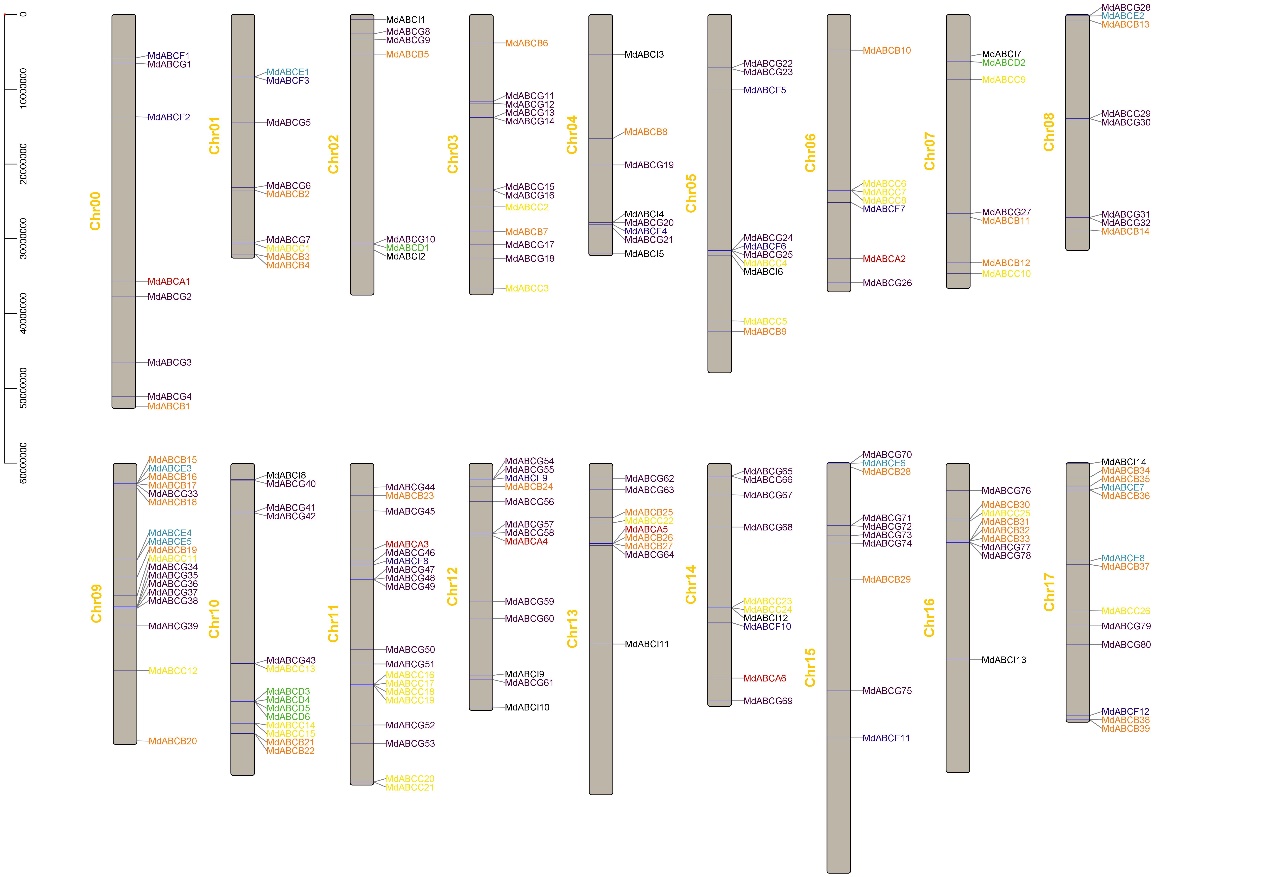


Supplemental Figure 3-2 Chromosomal location of ABC transporter genes in *Pyrus communis*


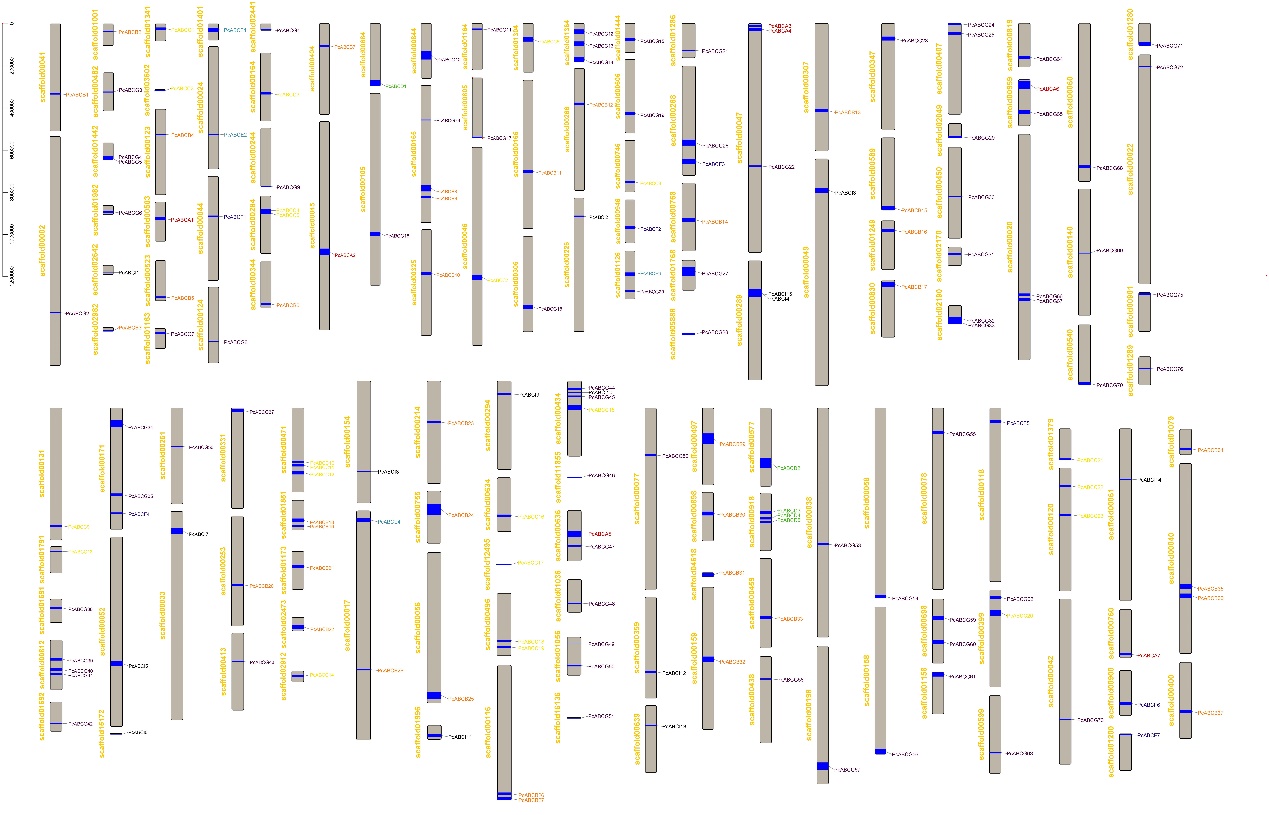


Supplemental Figure 3-3 Chromosomal location of ABC transporter genes in *Prunus persica*


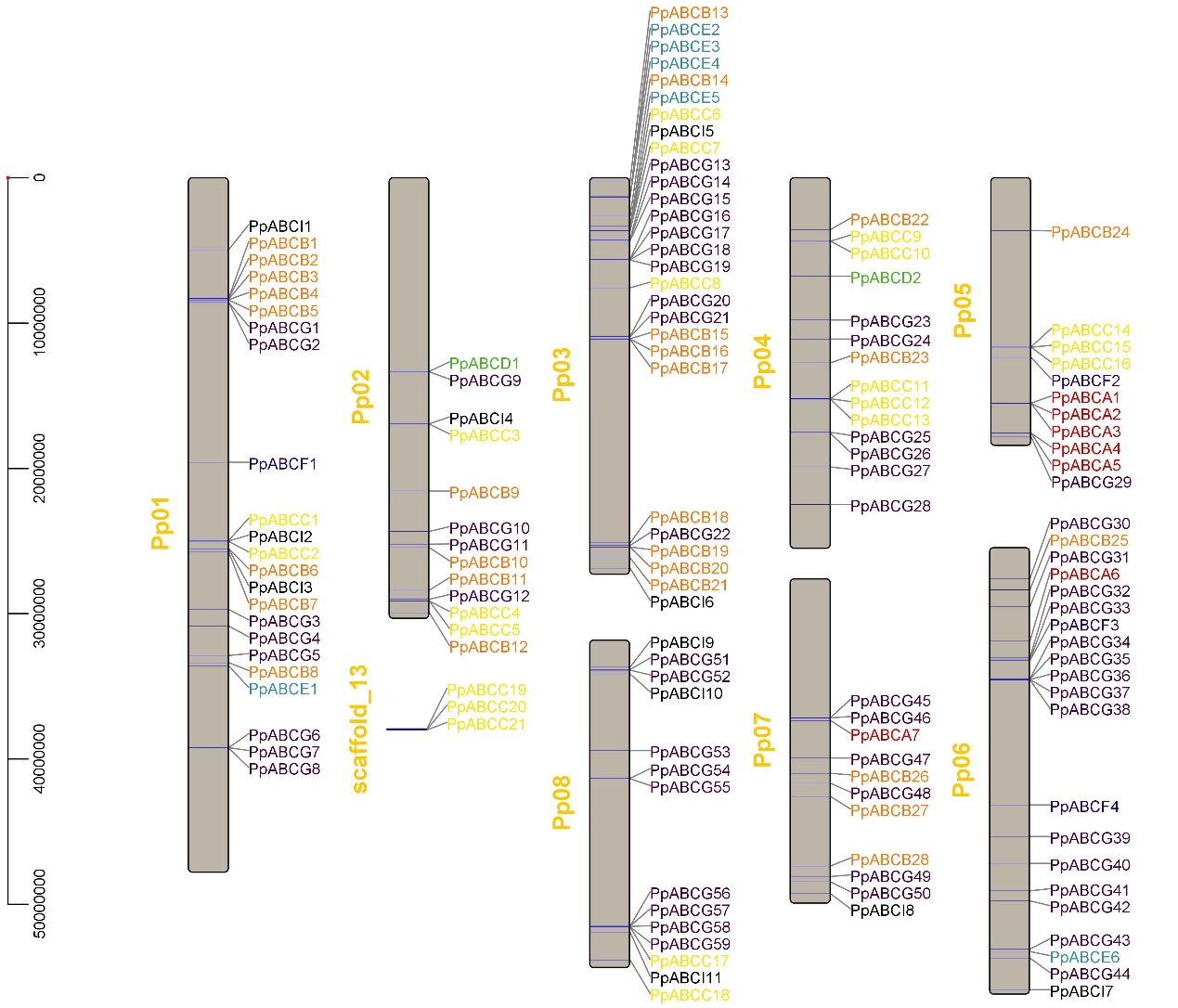


Supplemental Figure 3-4 Chromosomal location of ABC transporter genes in *Prunus avium*


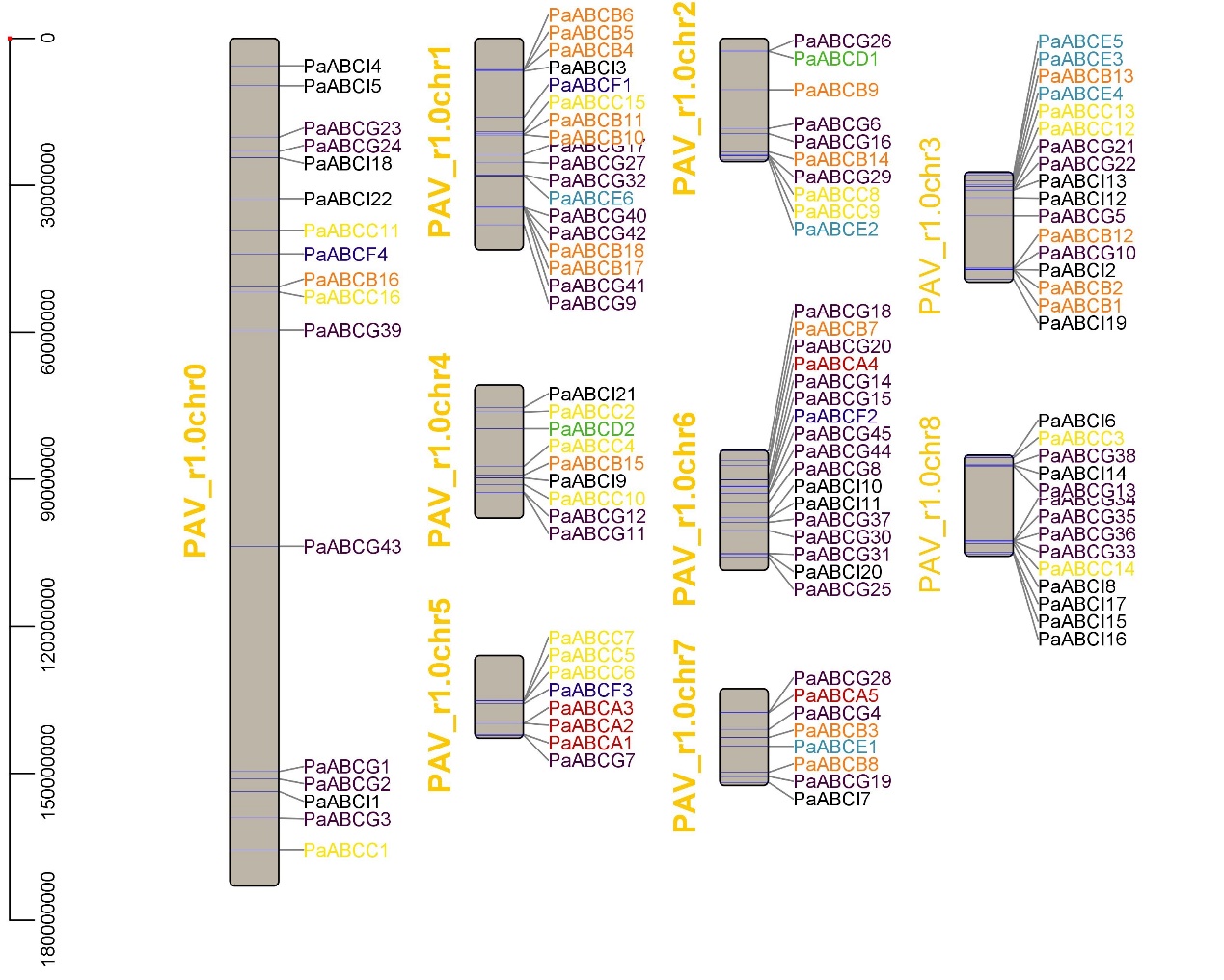


Supplemental Figure 3-5 Chromosomal location of ABC transporter genes in *Prunus dulcis*


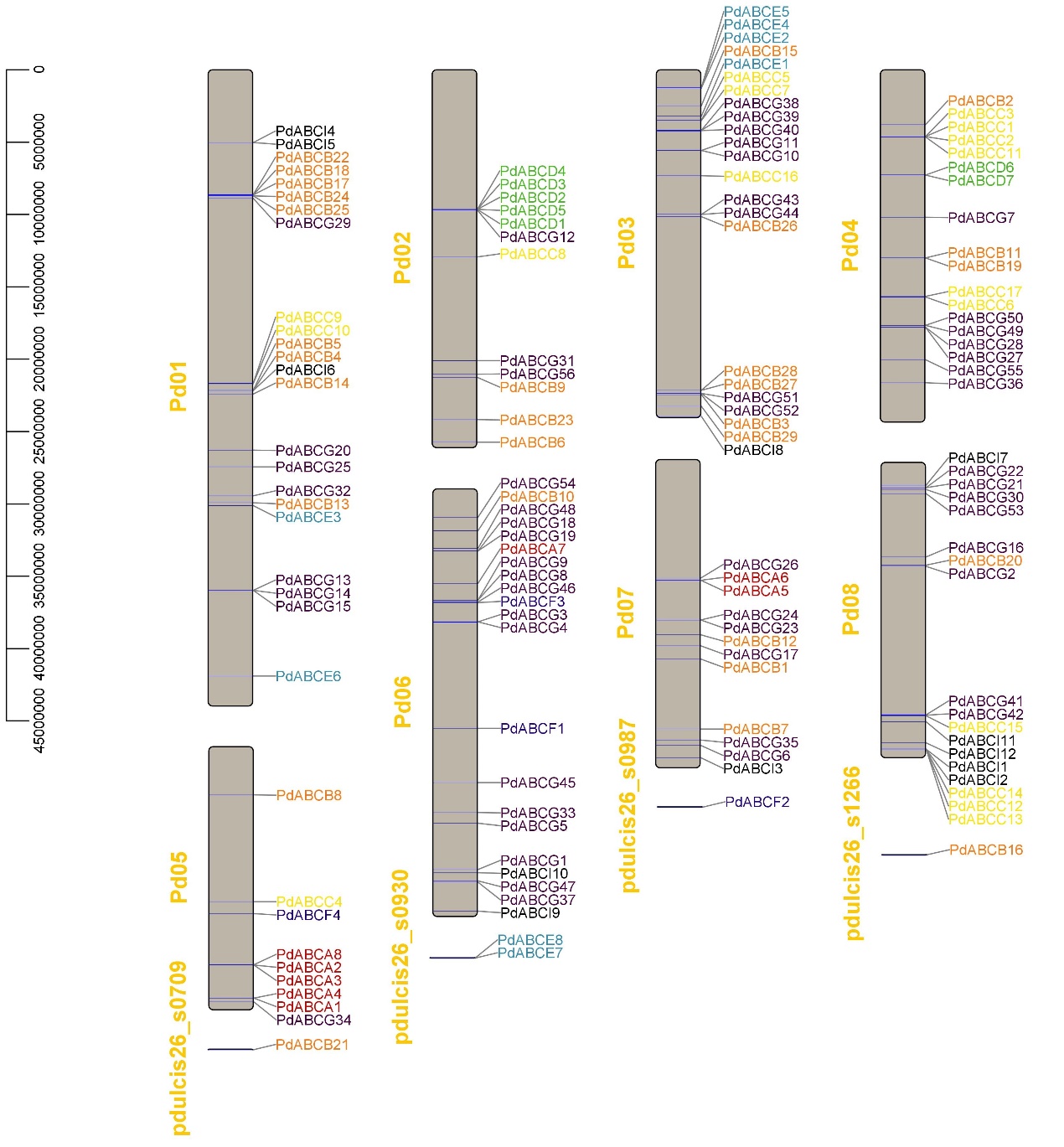


Supplemental Figure 3-6 Chromosomal location of ABC transporter genes in *Fragaria vesca*


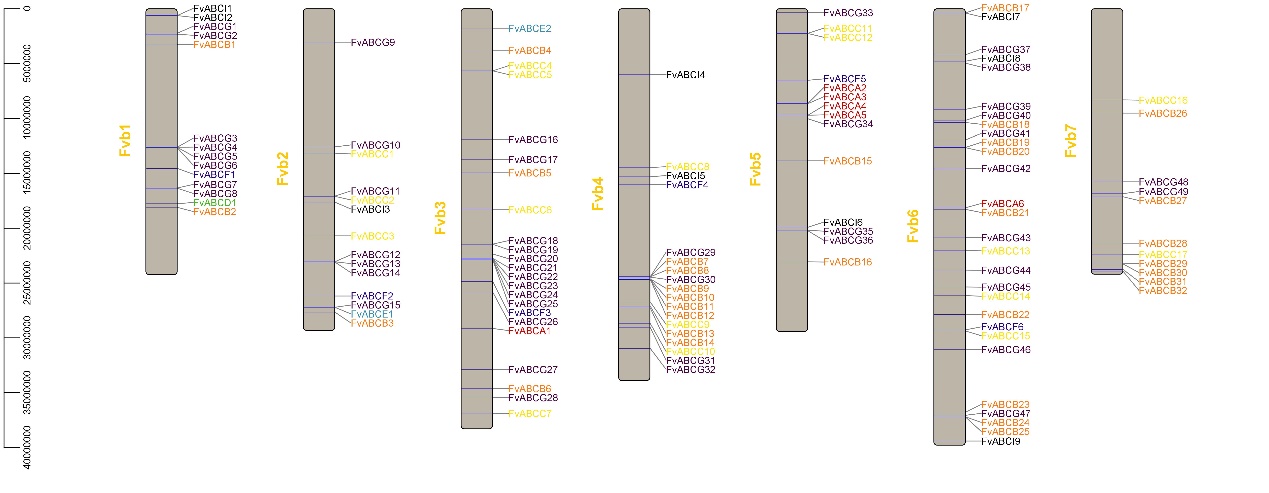


Supplemental Figure 3-7 Chromosomal location of ABC transporter genes in *Rubus occidentalis*


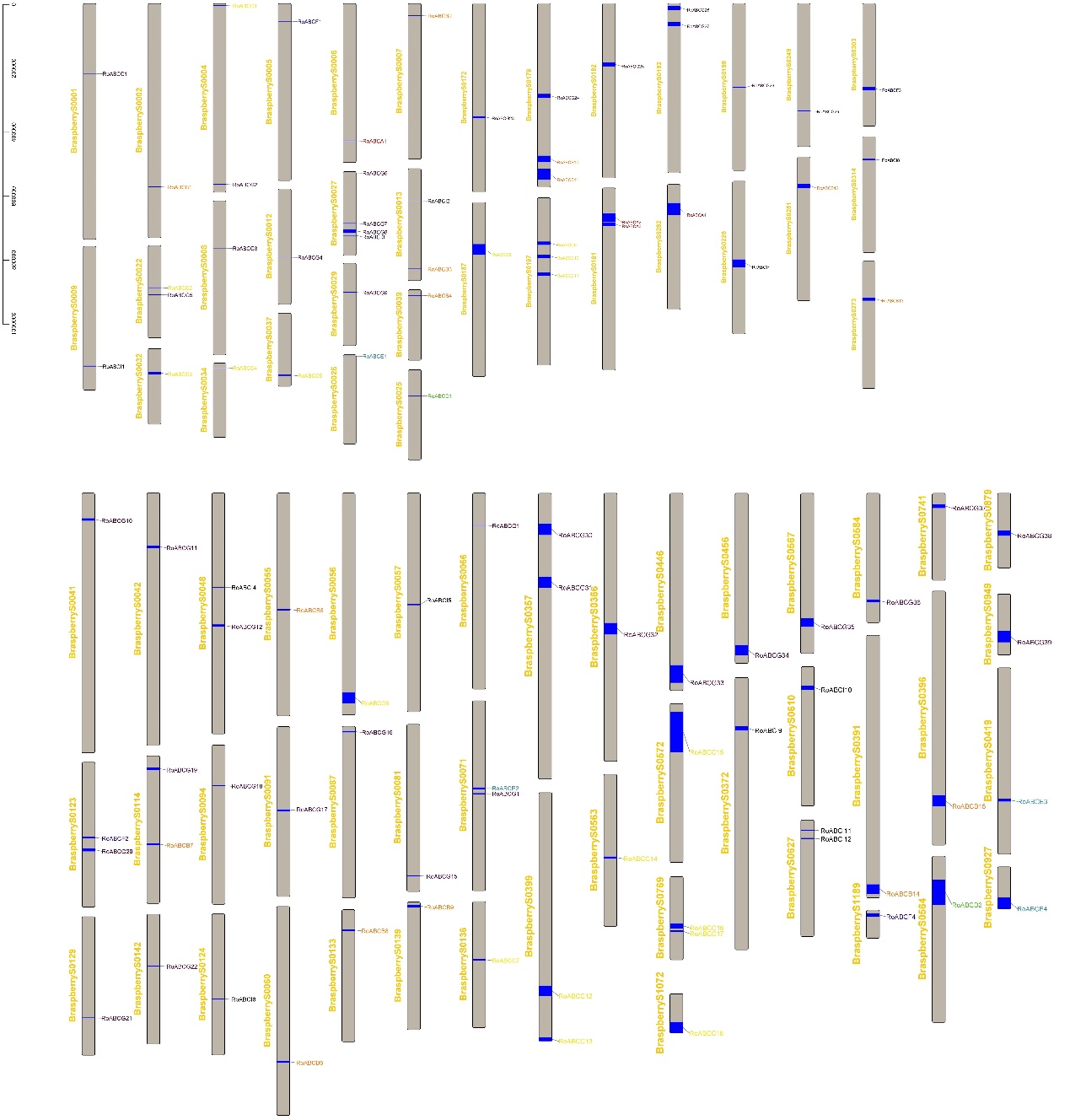


Supplemental Figure 3-8 Chromosomal location of ABC transporter genes in *Prunus mume*


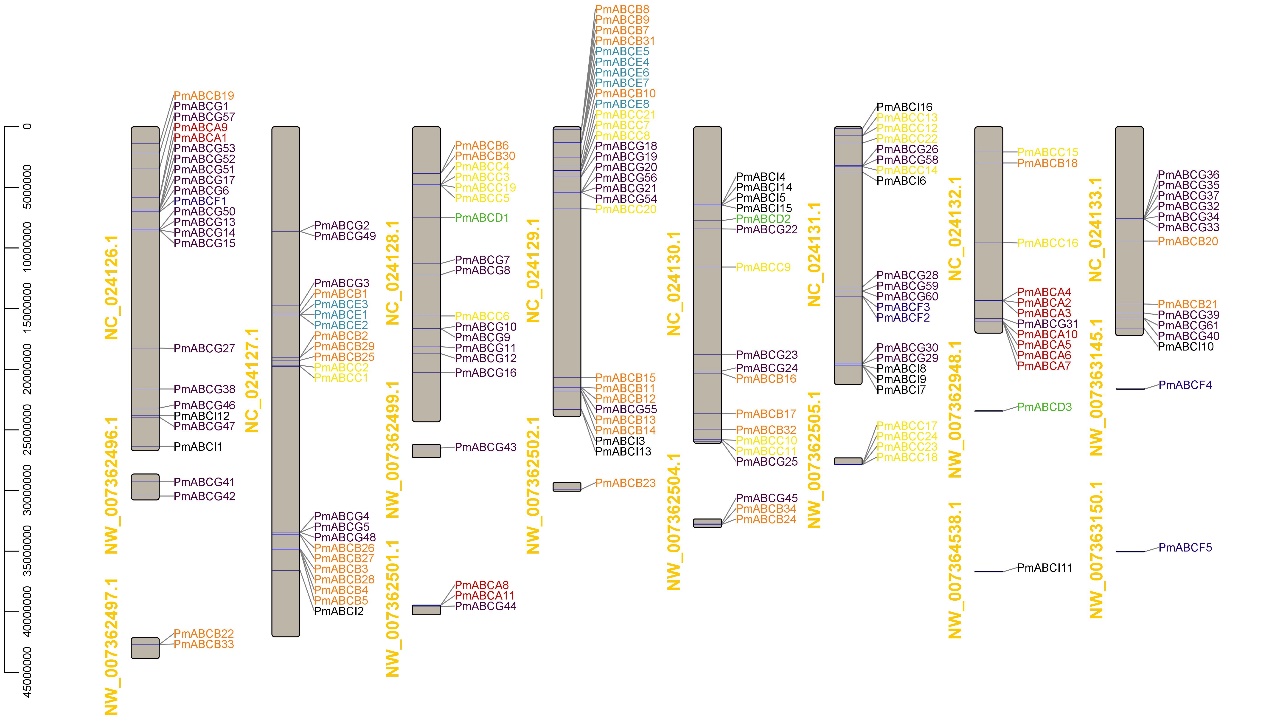


Supplemental Figure 3-9 Chromosomal location of ABC transporter genes in *Rosa chinensis*


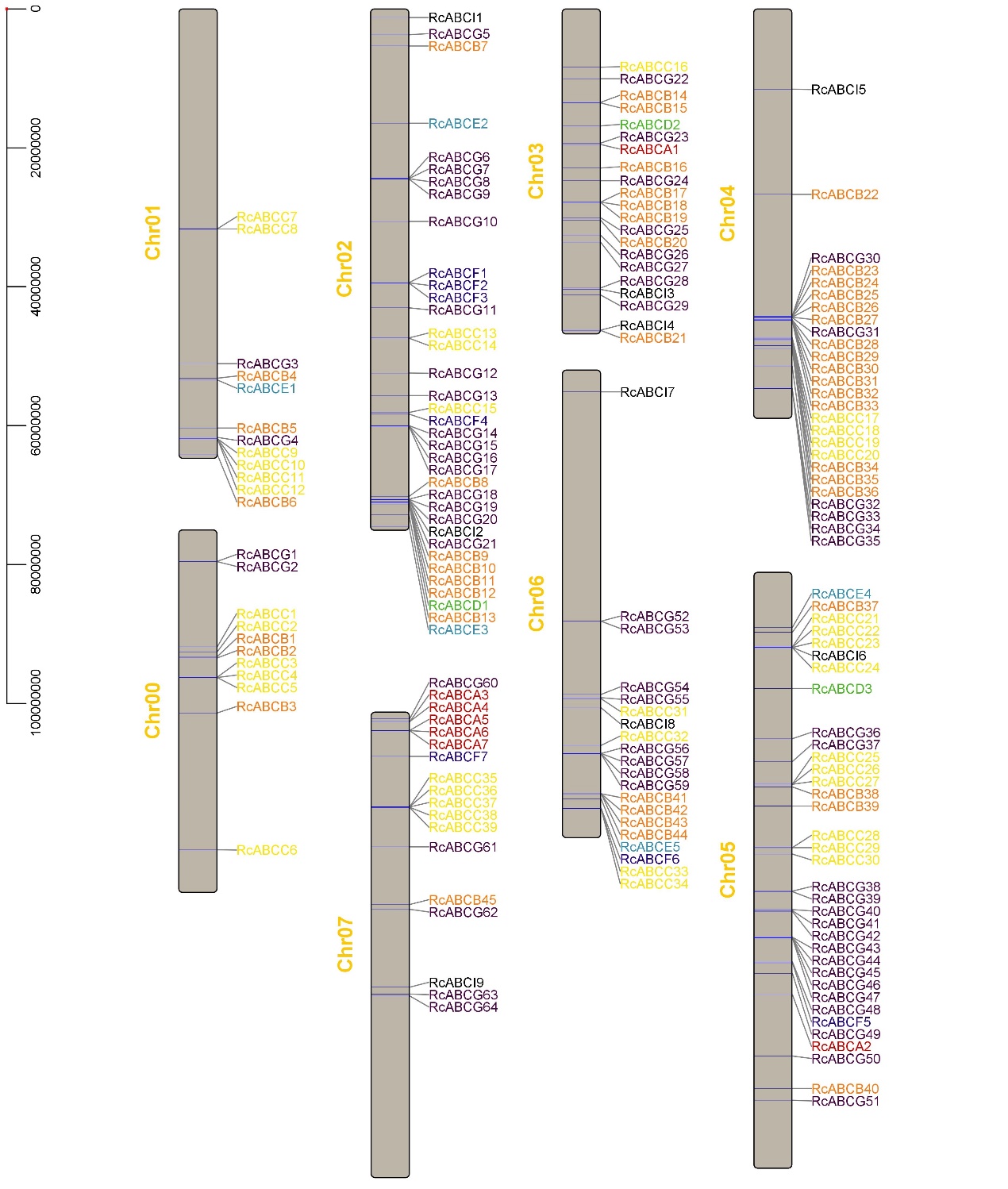

Supplement: Supplementary file 1 [file ijms-20-05783-s001.zip › Supplemental Figure 3.docx]
