# Supplementary material for: Genome-Wide Identification and Characterization of ABC Transporters in Nine Rosaceae Species Identifying MdABCG28 as a Possible Cytokinin Transporter linked to Dwarfing
Source: Int J Mol Sci. 2019 Nov 17;20(22):5783. doi: 10.3390/ijms20225783 (PMC6887749; doi:10.3390/ijms20225783)

Supplemental Figure 4. The identified motifs and gene structures of ABC transporter family members in nine Rosaceae species

Supplemental Figure 4-1 The identified motifs and gene structures of ABC transporter family members in *Malus domestica*


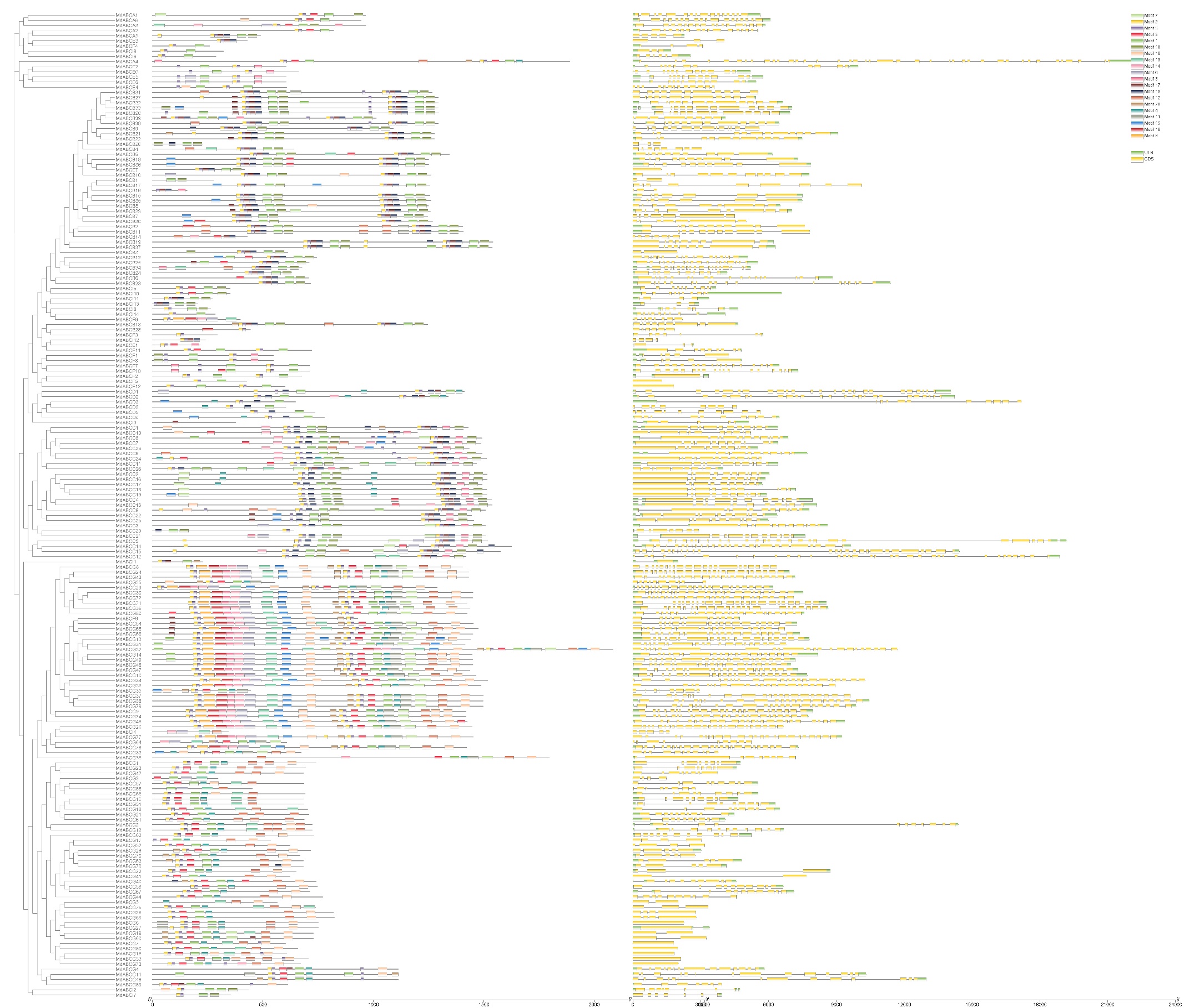


Supplemental Figure 4-2 The identified motifs and gene structures of ABC transporter family members in *Pyrus communis*


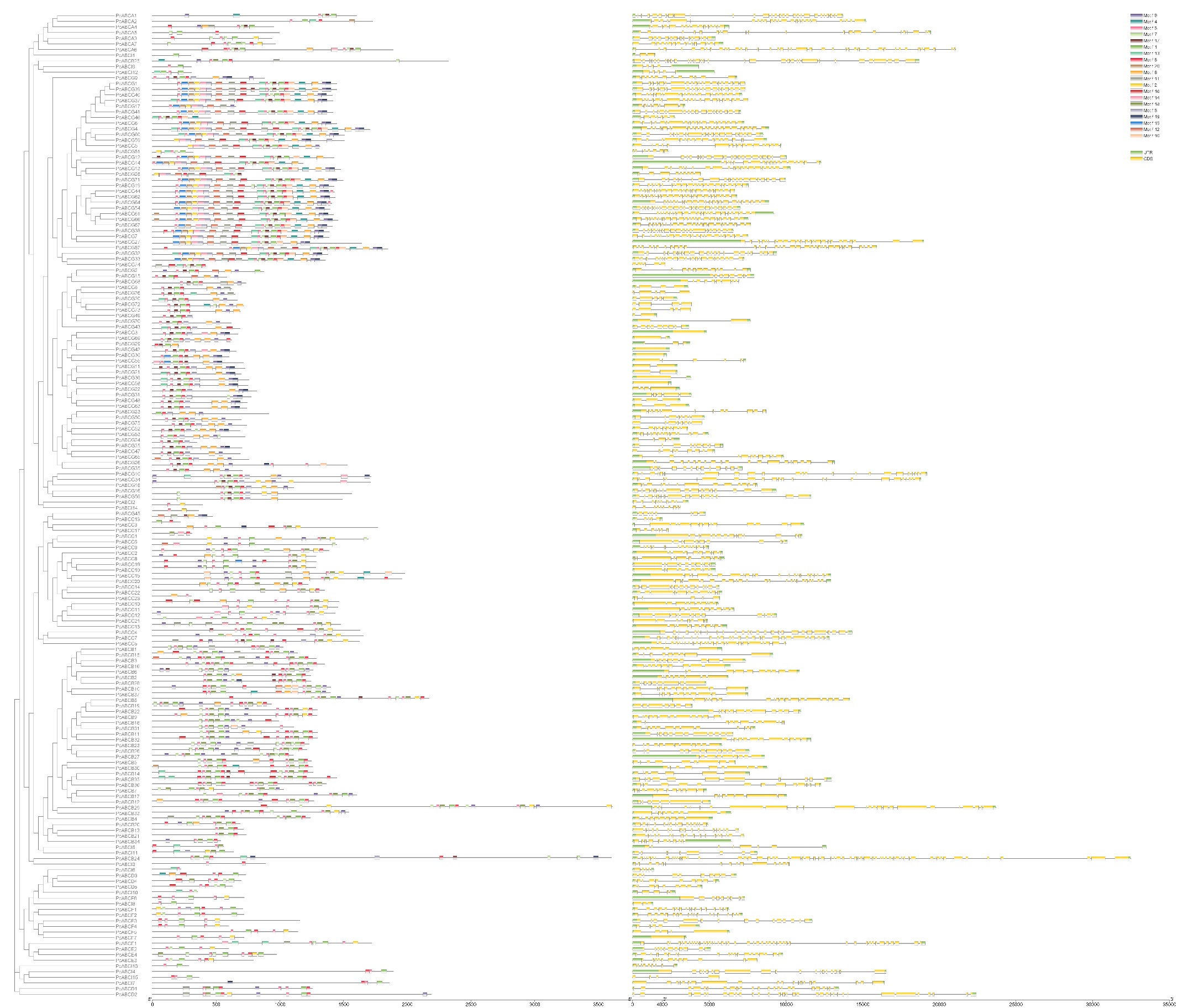


Supplemental Figure 4-3 The identified motifs and gene structures of ABC transporter family members in *Prunus persica*


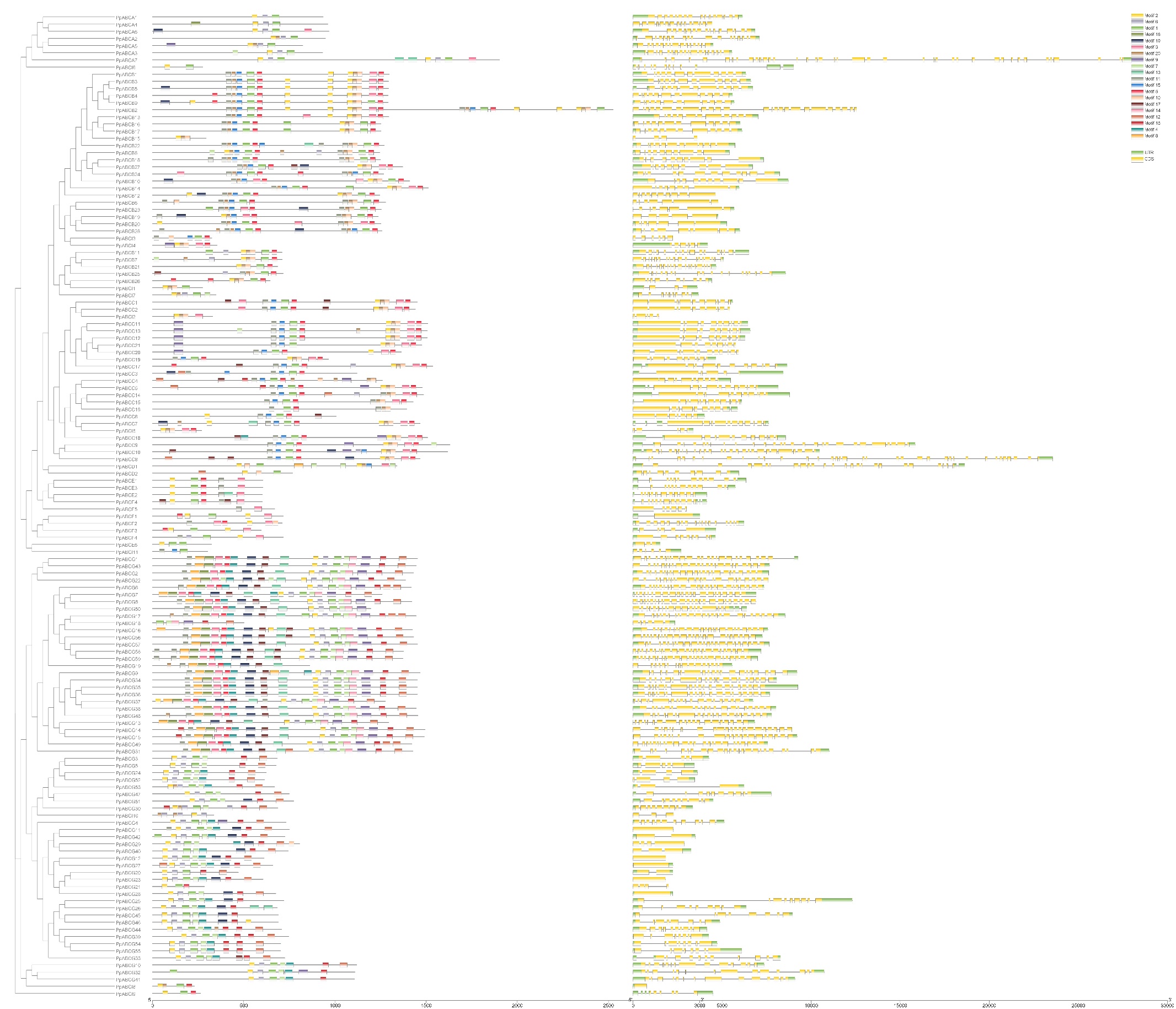


Supplemental Figure 4-4 The identified motifs and gene structures of ABC transporter family members in *Prunus avium*


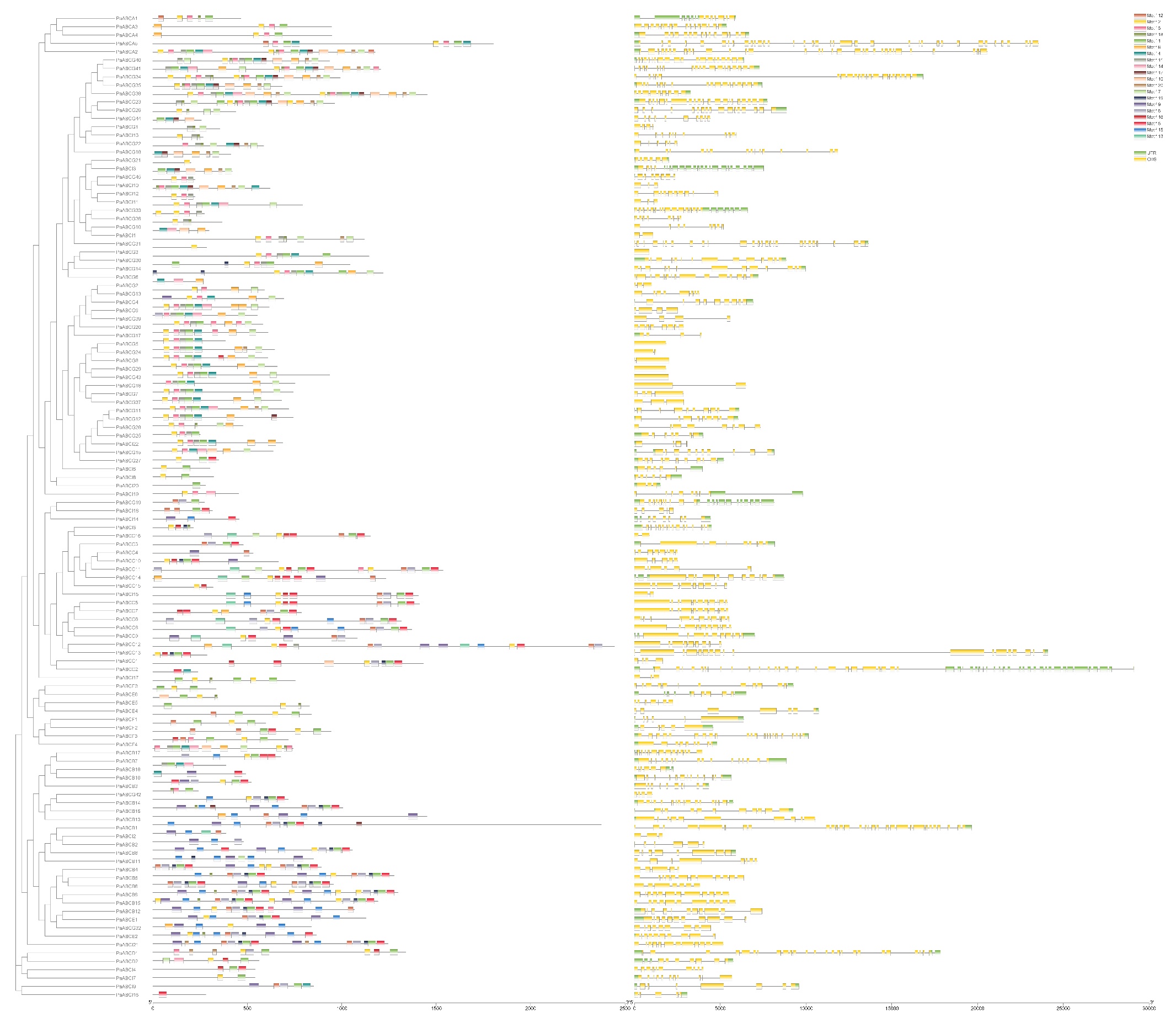


Supplemental Figure 4-5 The identified motifs and gene structures of ABC transporter family members in *Prunus dulcis*


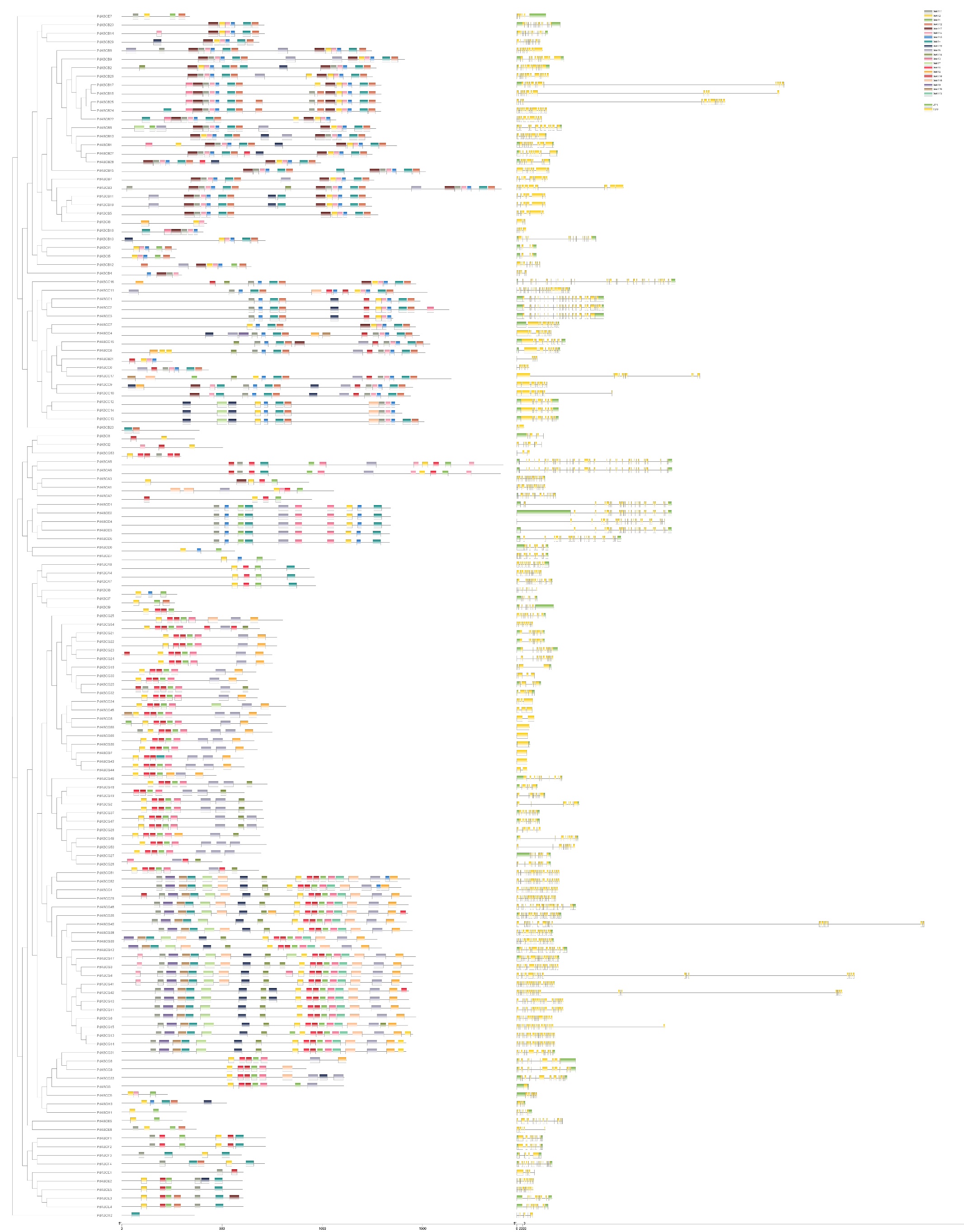


Supplemental Figure 4-6 The identified motifs and gene structures of ABC transporter family members in *Fragaria vesca*


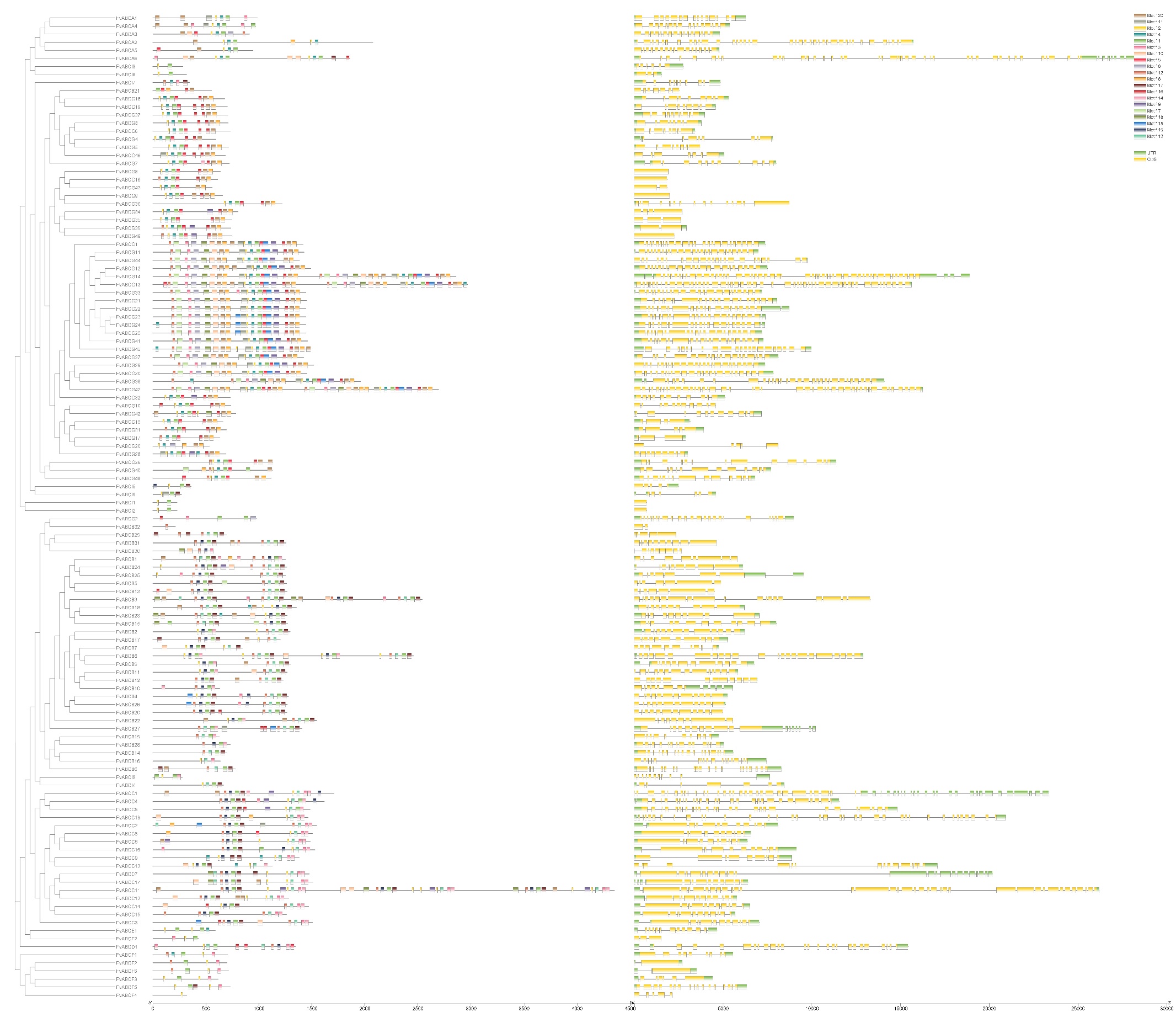


Supplemental Figure 4-7 The identified motifs and gene structures of ABC transporter family members in *Rubus occidentalis*


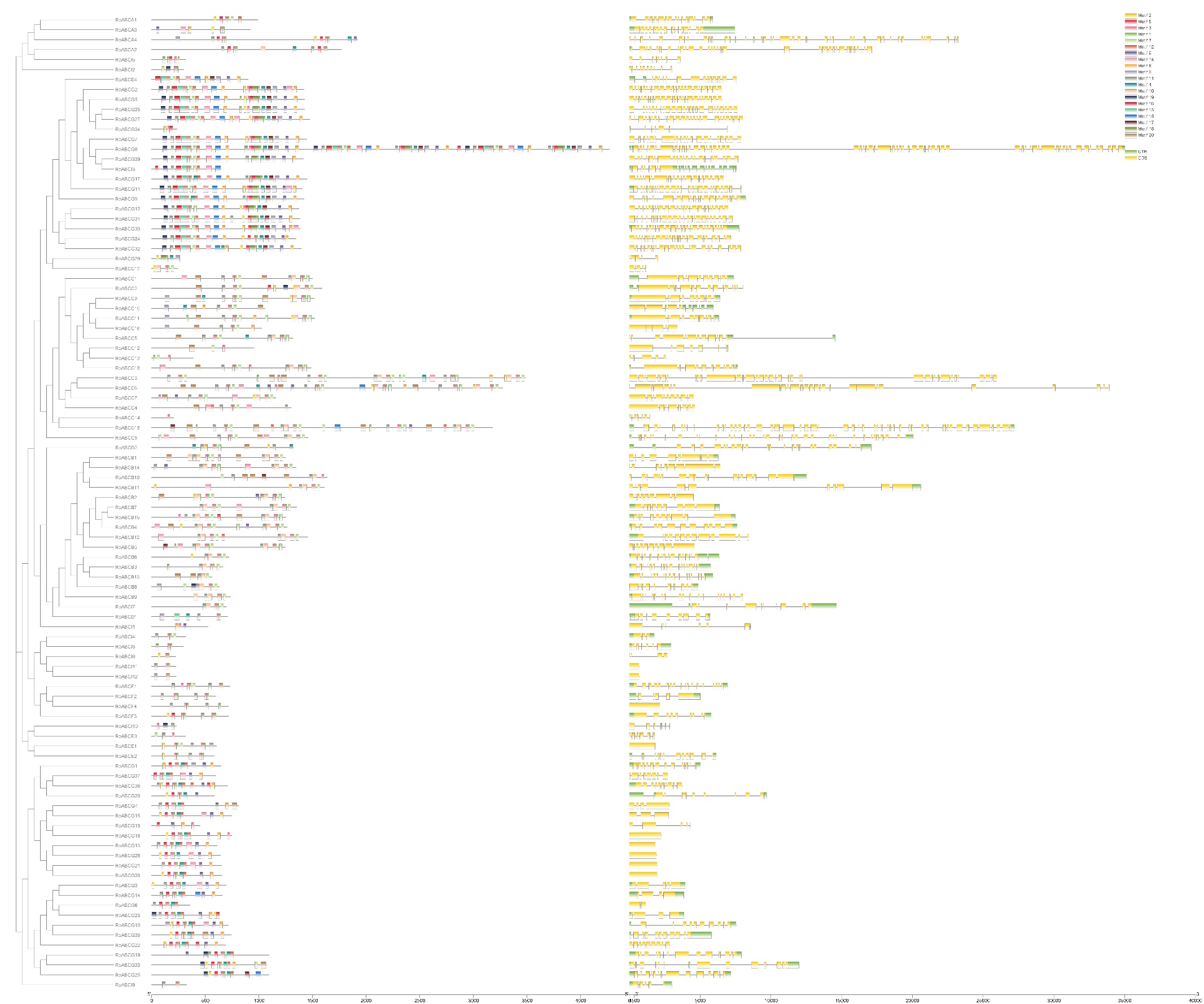


Supplemental Figure 4-8 The identified motifs and gene structures of ABC transporter family members in *Prunus mume*


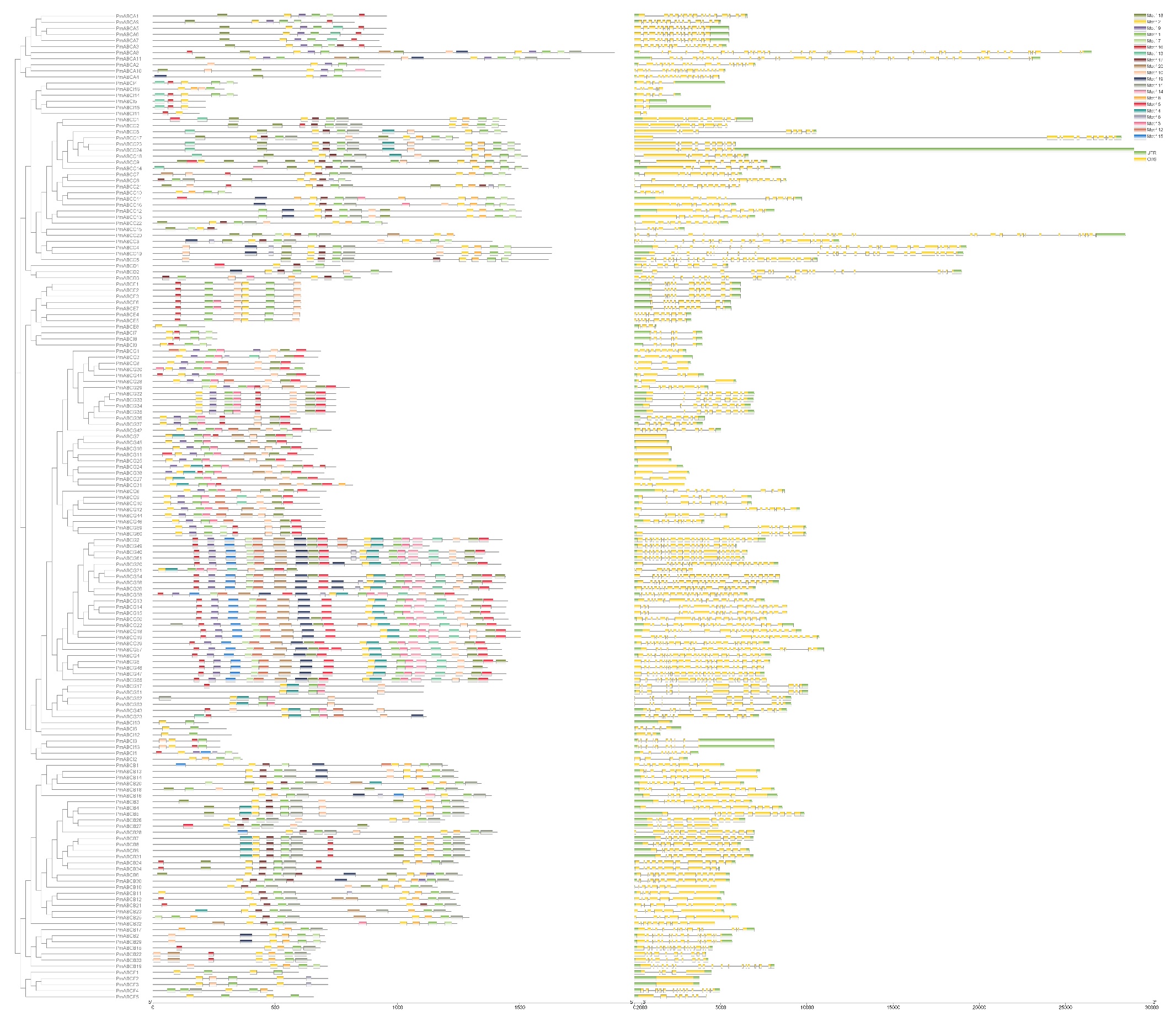


Supplemental Figure 4-9 The identified motifs and gene structures of ABC transporter family members in *Rosa chinensis*


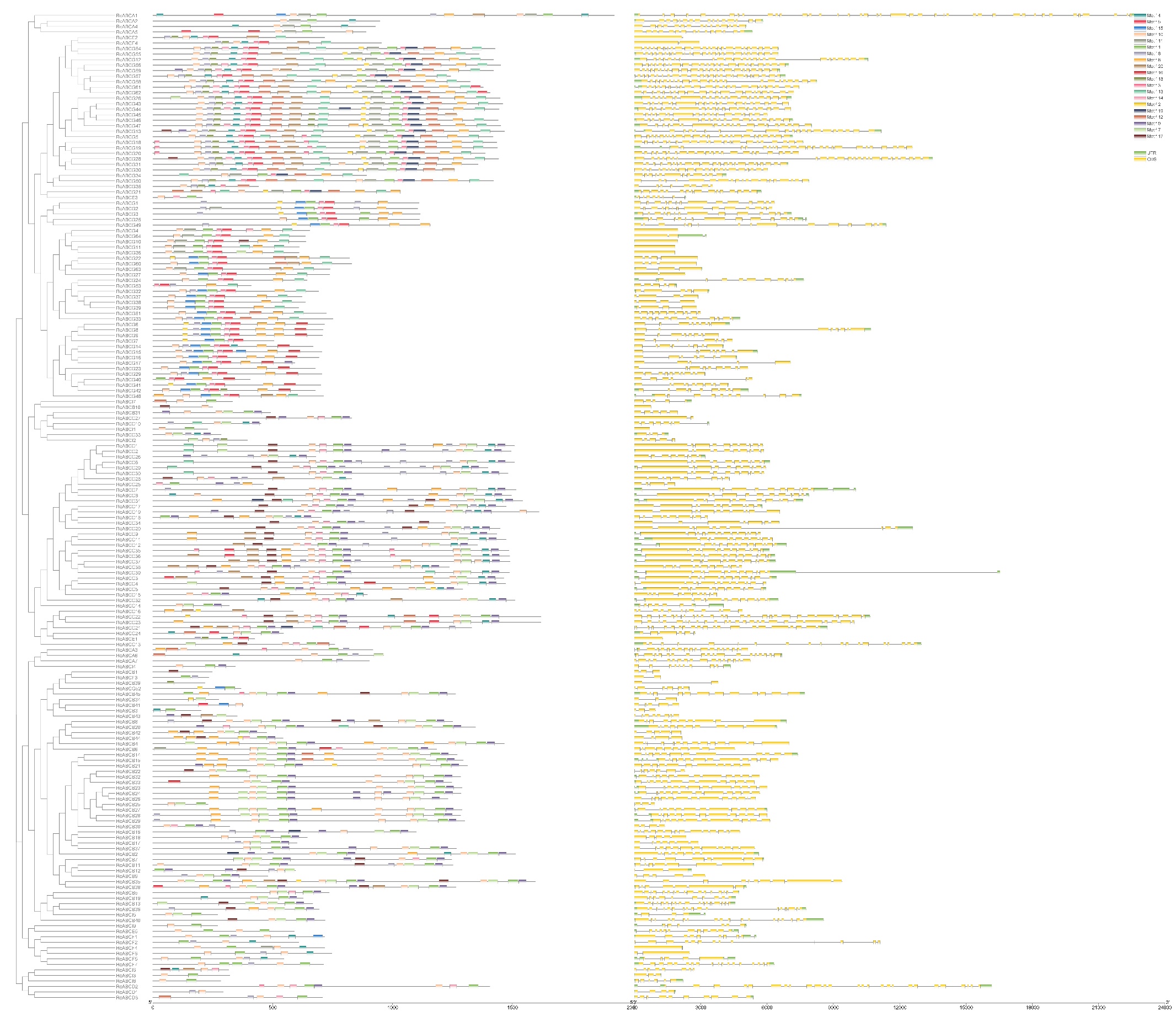

Supplement: Supplementary file 1 [file ijms-20-05783-s001.zip › Supplemental Figure 4.docx]
