# Supplementary material for: Genome-Wide Identification and Characterization of ABC Transporters in Nine Rosaceae Species Identifying MdABCG28 as a Possible Cytokinin Transporter linked to Dwarfing
Source: Int J Mol Sci. 2019 Nov 17;20(22):5783. doi: 10.3390/ijms20225783 (PMC6887749; doi:10.3390/ijms20225783)

Supplemental Figure 5. Tandem duplication and segmental duplication of ABC transporter family members in nine Rosaceae species

Supplemental Figure 5-1 Tandem duplication and segmental duplication of ABC transporter family members in *Malus domestica*


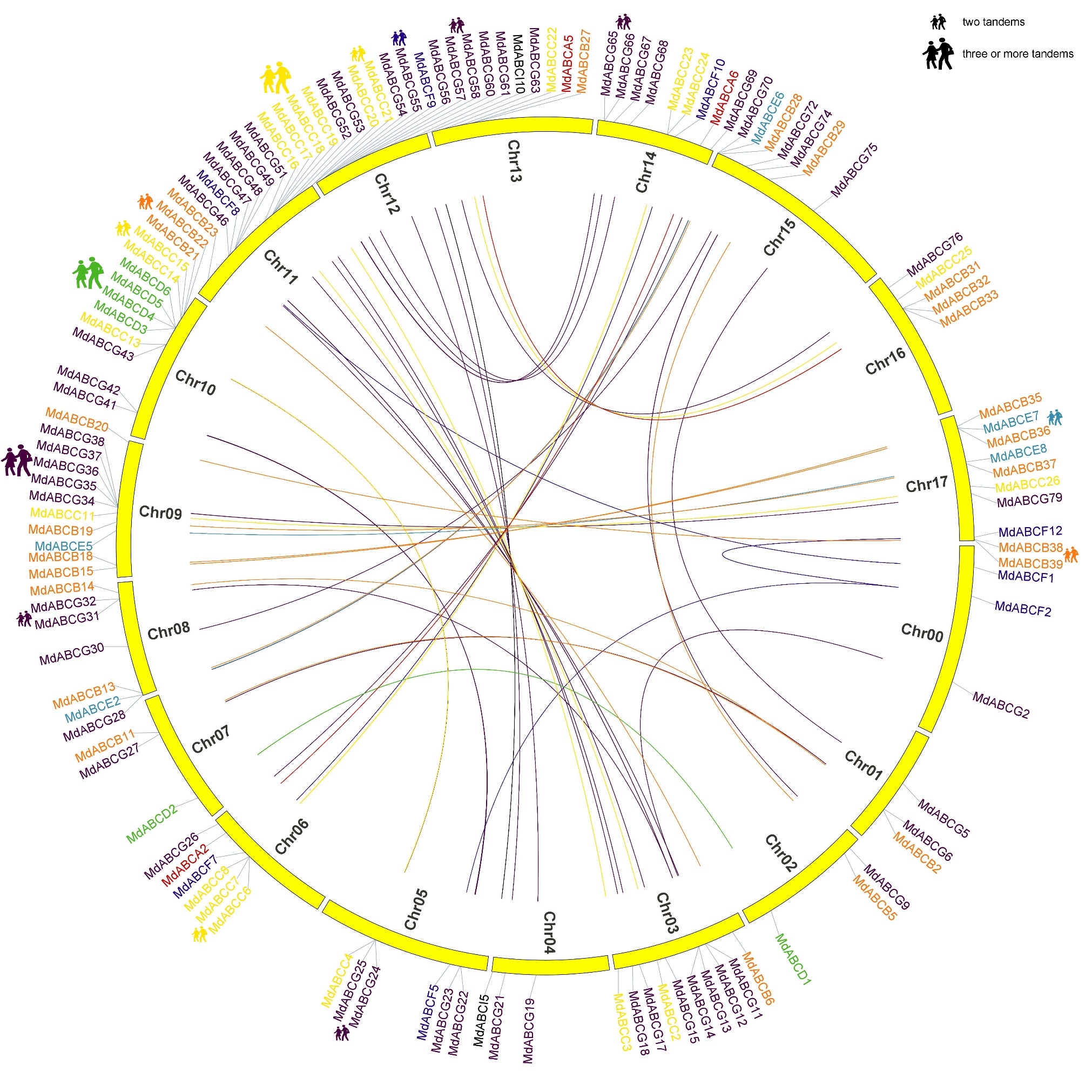


Supplemental Figure 5-2 Tandem duplication and segmental duplication of ABC transporter family members in *Pyrus communis*


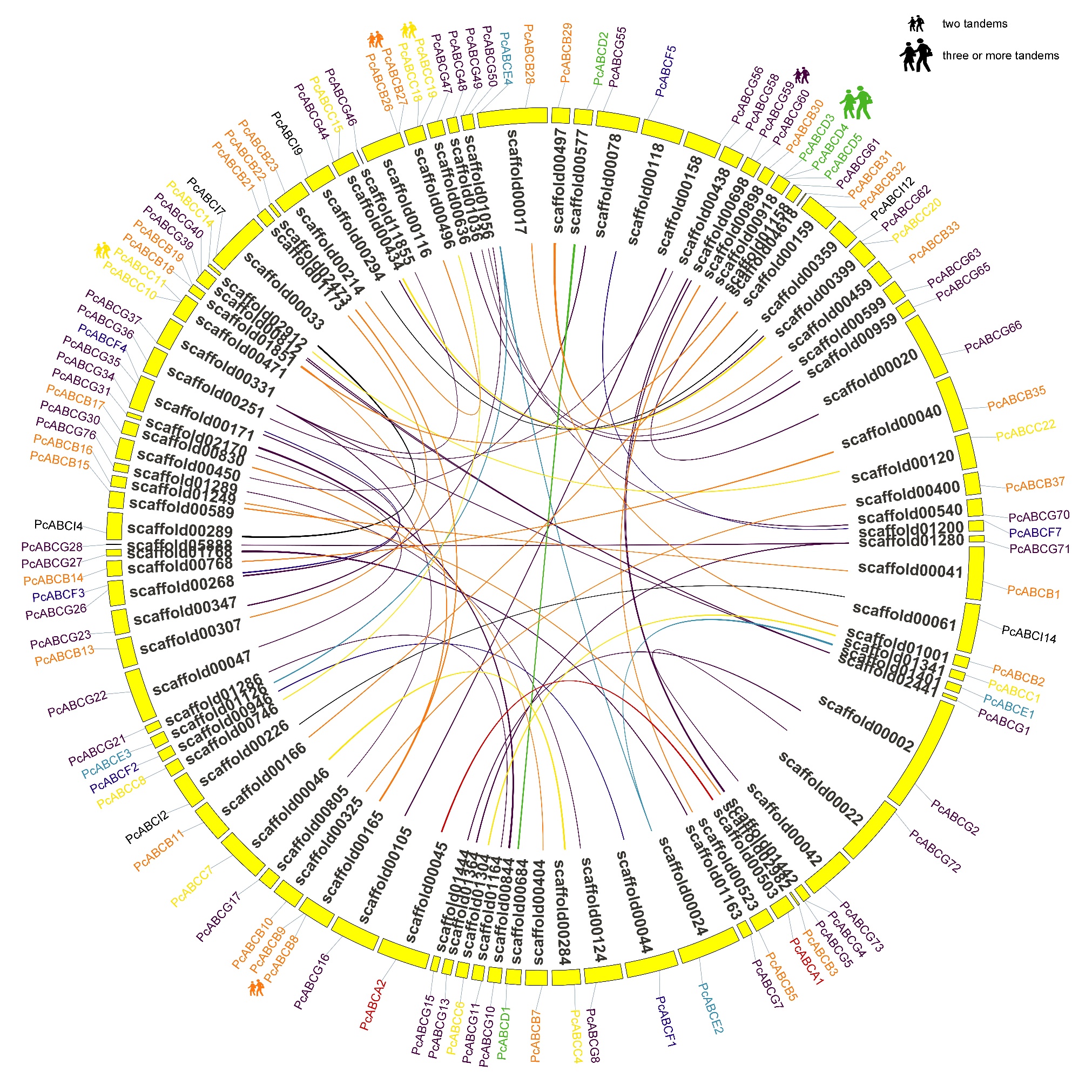


Supplemental Figure 5-3 Tandem duplication and segmental duplication of ABC transporter family members in *Prunus persica*


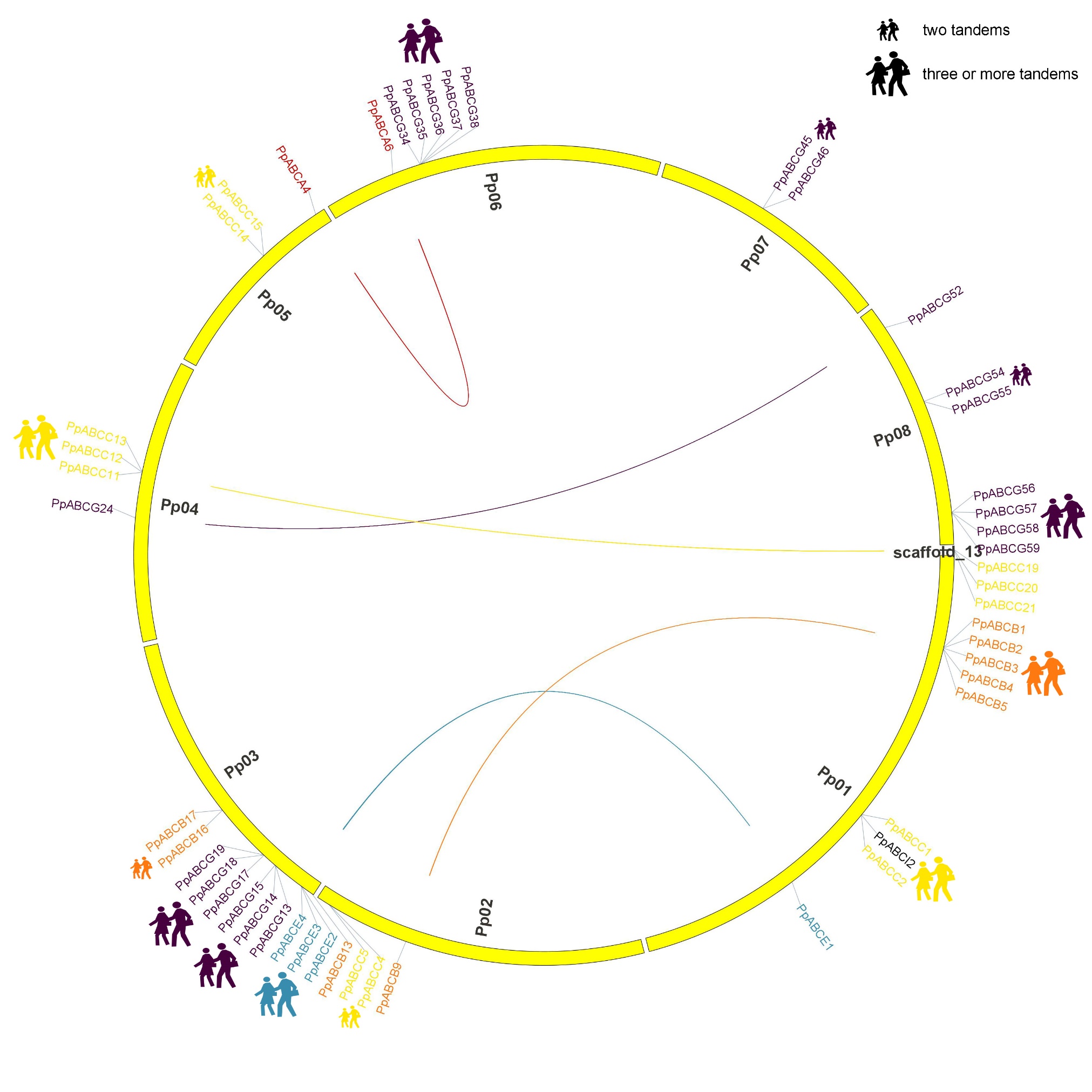


Supplemental Figure 5-4 Tandem duplication and segmental duplication of ABC transporter family members in *Prunus avium*


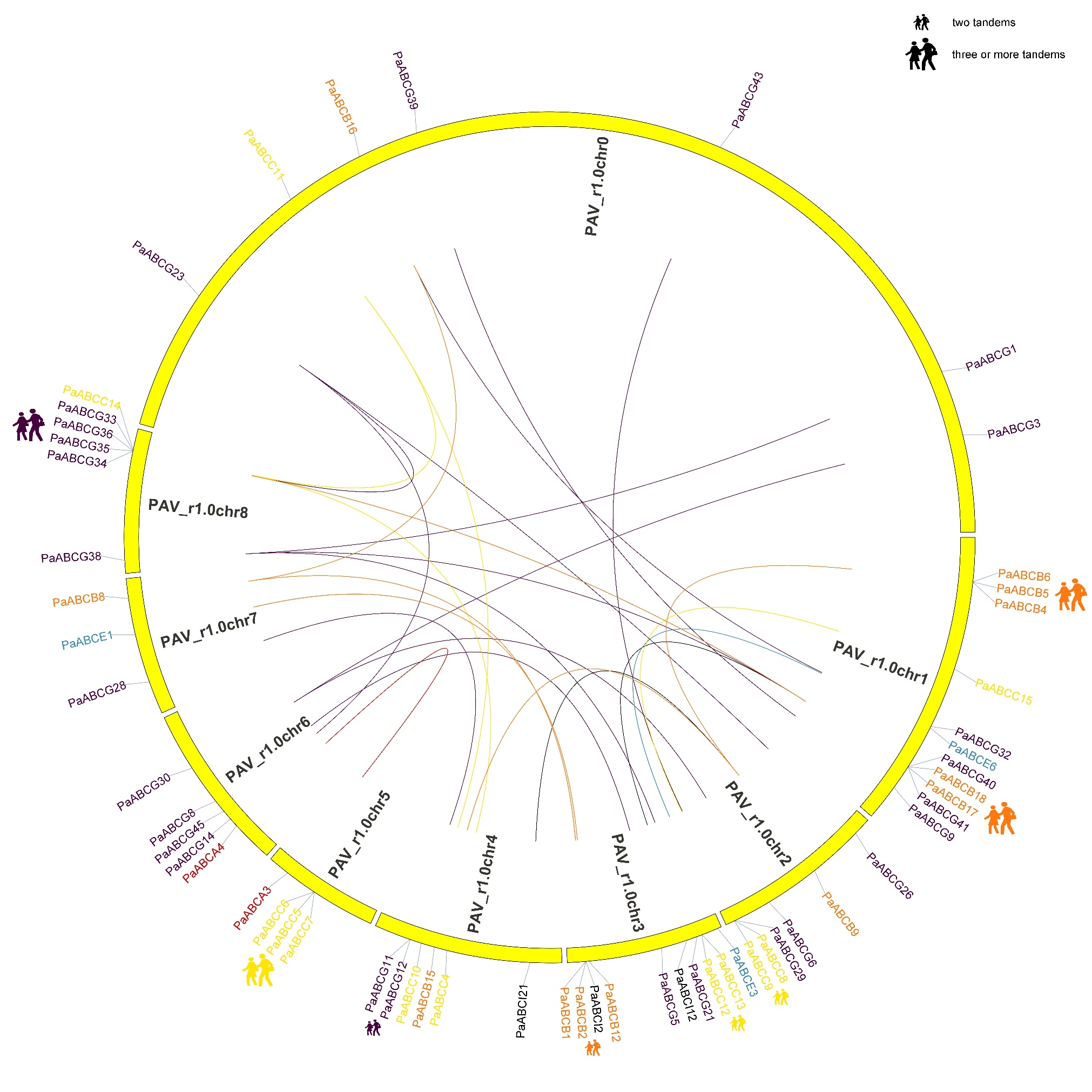


Supplemental Figure 5-5 Tandem duplication and segmental duplication of ABC transporter family members in *Prunus dulcis*


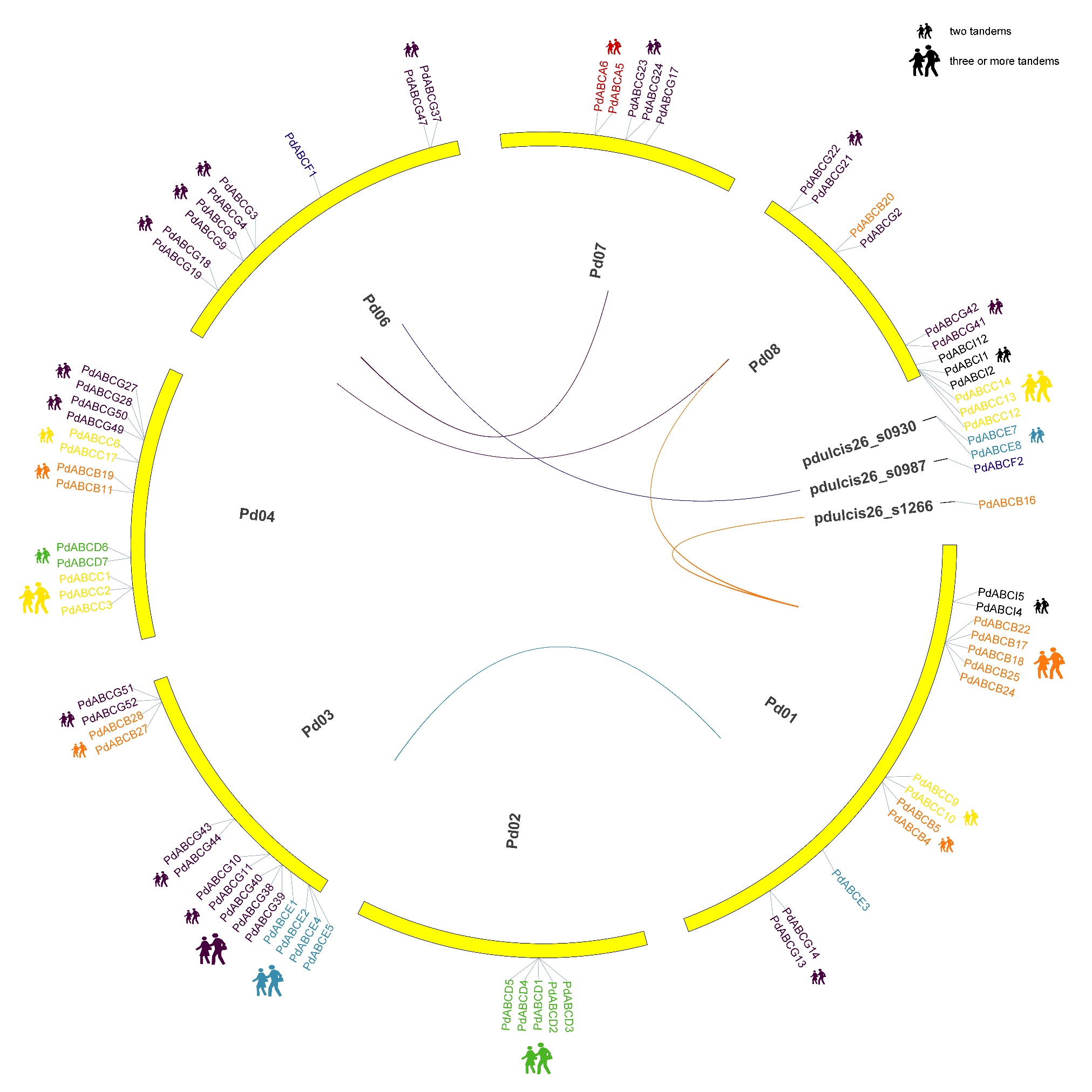


Supplemental Figure 5-6 Tandem duplication and segmental duplication of ABC transporter family members in *Fragaria vesca*


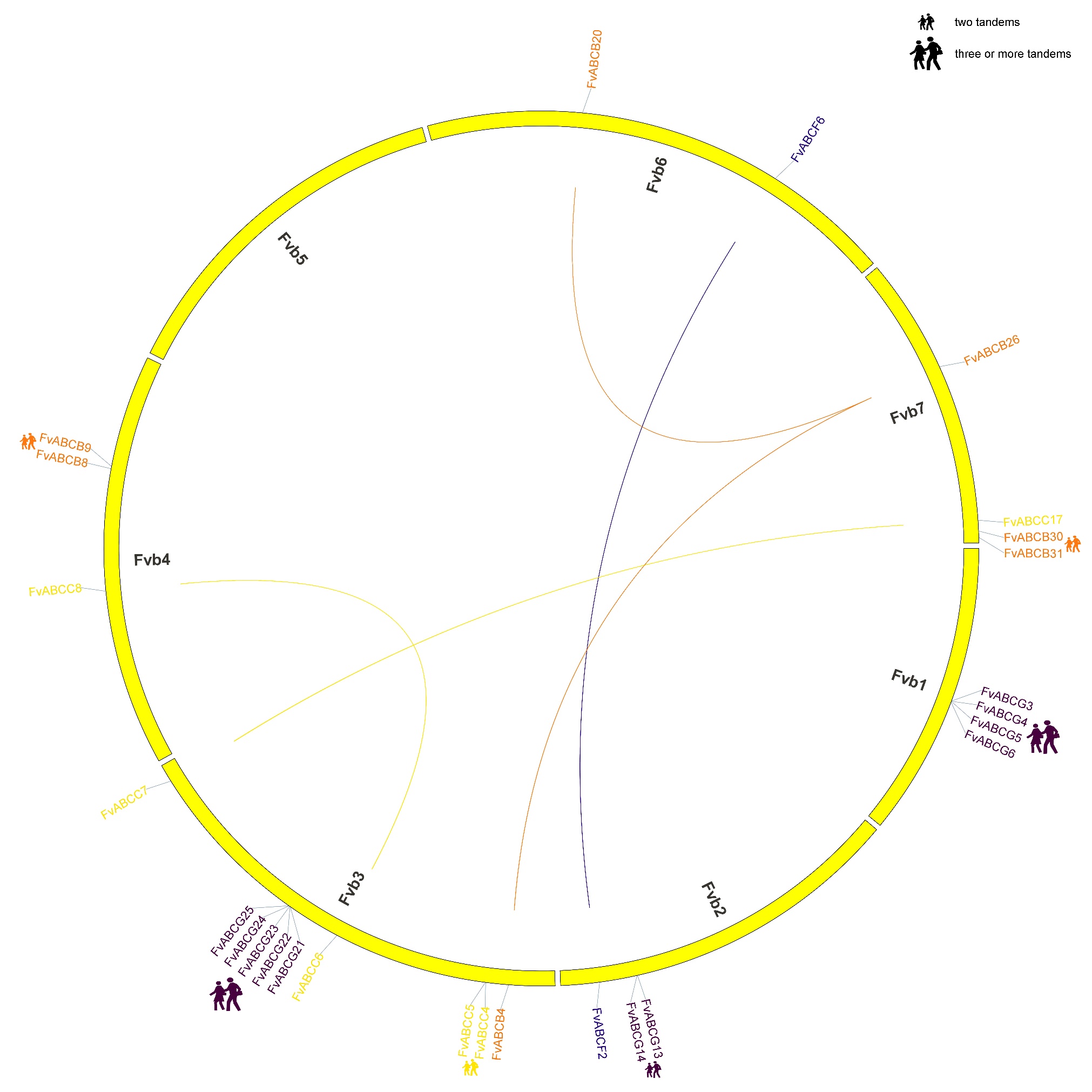


Supplemental Figure 5-7 Tandem duplication and segmental duplication of ABC transporter family members in *Rubus occidentalis*


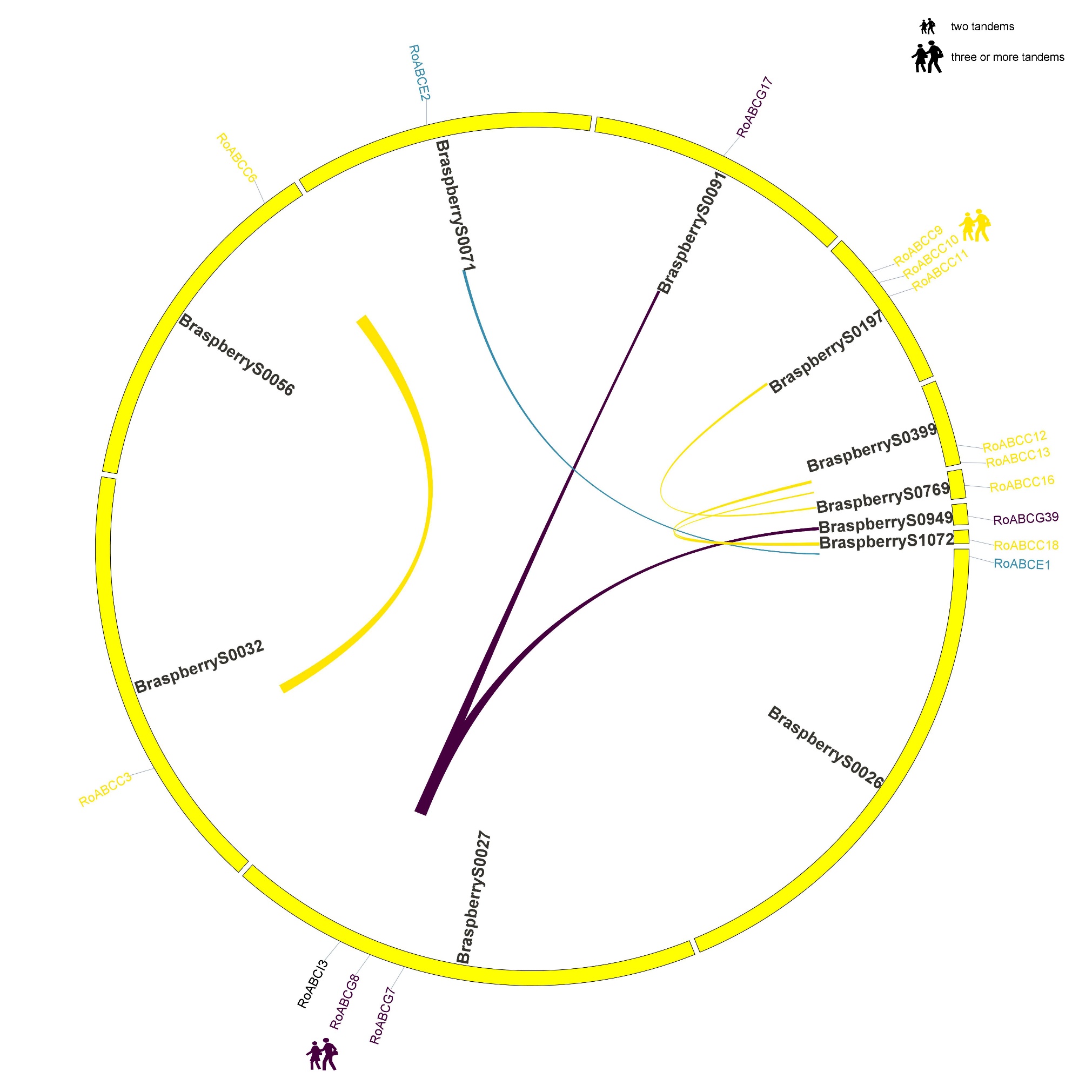


Supplemental Figure 5-8 Tandem duplication and segmental duplication of ABC transporter family members in *Prunus mume*


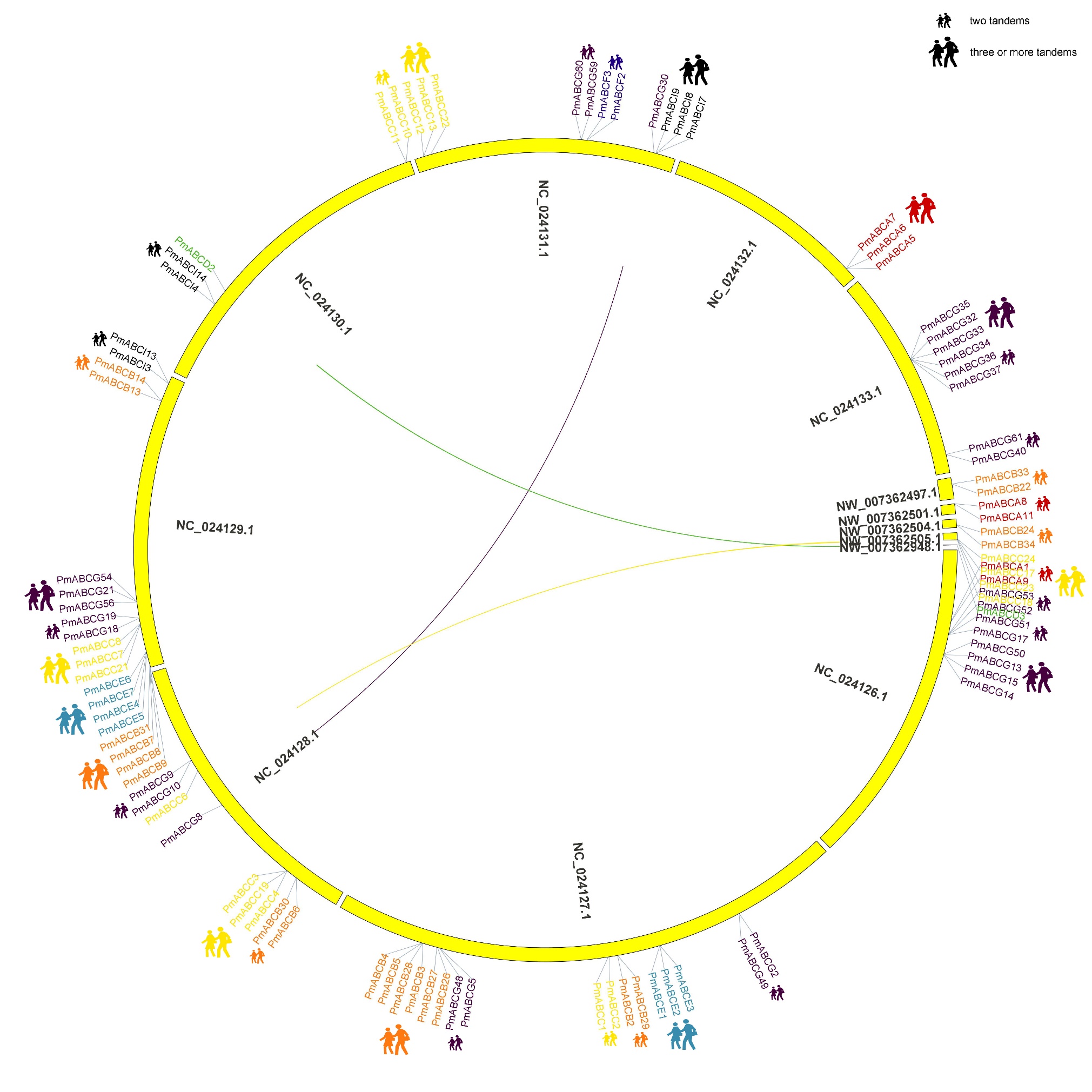


Supplemental Figure 5-9 Tandem duplication and segmental duplication of ABC transporter family members in *Rosa chinensis*


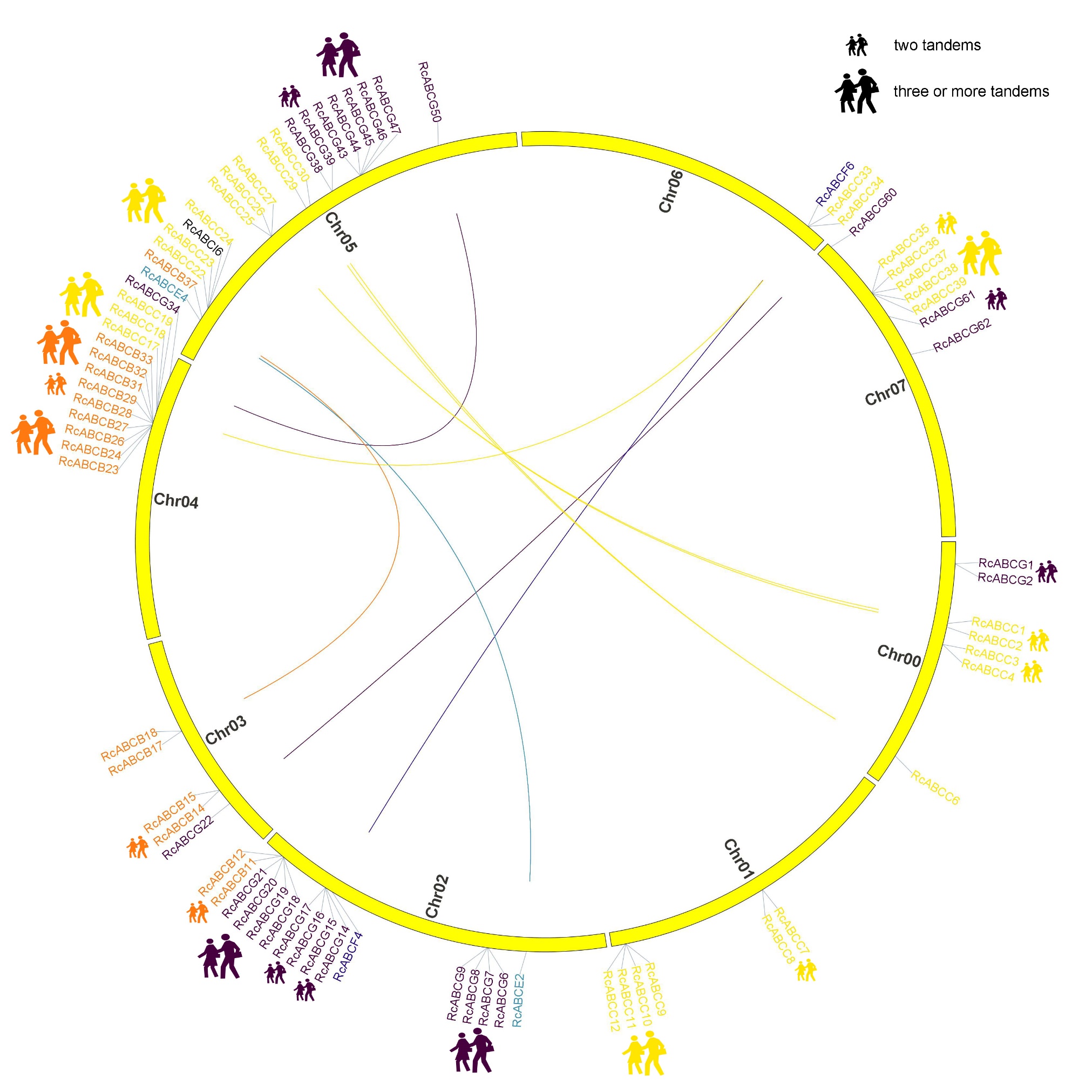

Supplement: Supplementary file 1 [file ijms-20-05783-s001.zip › Supplemental Figure 5.docx]
