# Supplementary material for: Genome-Wide Identification and Characterization of ABC Transporters in Nine Rosaceae Species Identifying MdABCG28 as a Possible Cytokinin Transporter linked to Dwarfing
Source: Int J Mol Sci. 2019 Nov 17;20(22):5783. doi: 10.3390/ijms20225783 (PMC6887749; doi:10.3390/ijms20225783)
Supplement: Supplementary file 1 [file ijms-20-05783-s001.zip › Supplemental Table 2.docx]

Supplemental Table 2. Motifs information of ABC transporter family members in nine Rosaceae species

Supplemental Table 2-1 Motifs information of ABC transporter family members in *Malus domestica*

| Motif | LOGO | Sequence | E-value | Sites | Width |
| --- | --- | --- | --- | --- | --- |
| 1 | 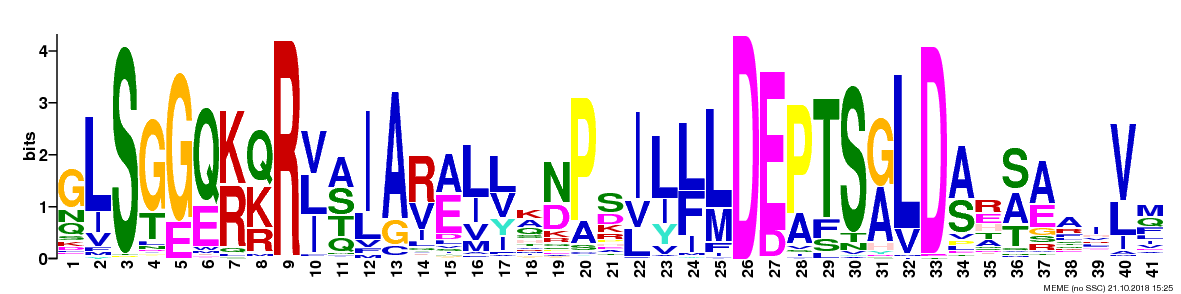 | GLSGGQKQRVAIARALLKBPSILLLDEPTSGLDARSAAIVM | 4.5e-1932 | 176 | 41 |
| 2 | 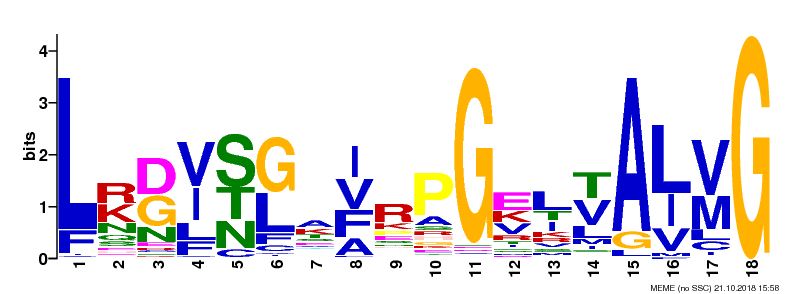 | LRDVSGAIRPGELTALVG | 5.6e-733 | 152 | 18 |
| 3 | 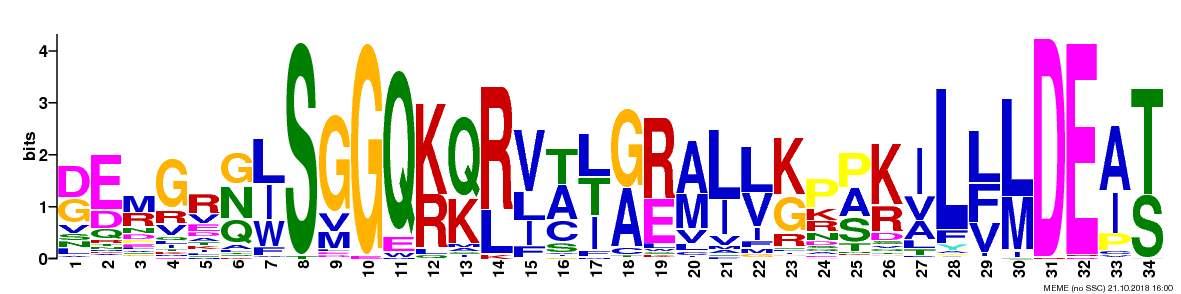 | DEMGRGLSGGQKQRVTLGRALLKPPKILLLDEAT | 4.8e-807 | 98 | 34 |
| 4 | 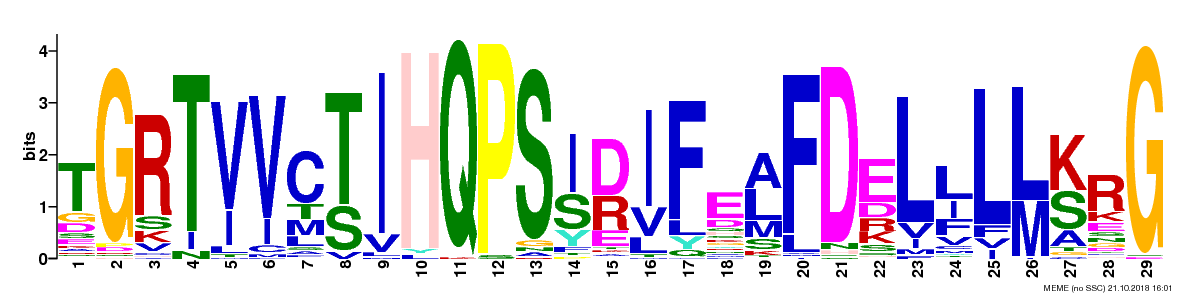 | TGRTVVCTIHQPSIDIFEAFDELLLLKRG | 1.4e-711 | 80 | 29 |
| 5 | 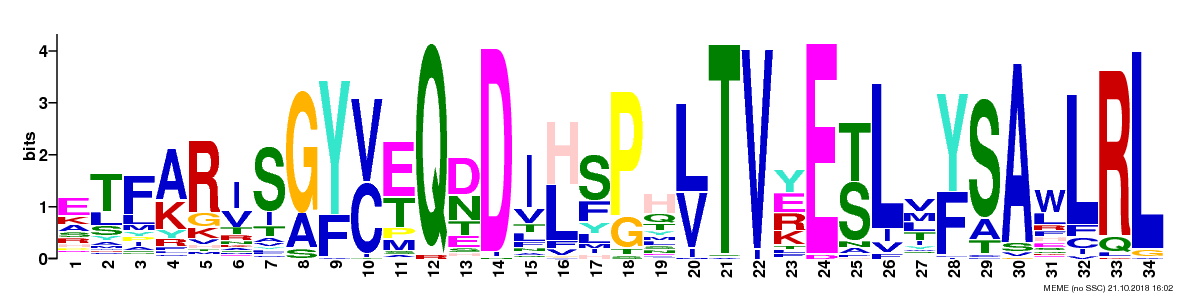 | ETFARISGYVEQBDIHSPHLTVYETLVYSAWLRL | 7.9e-743 | 81 | 34 |
| 6 | 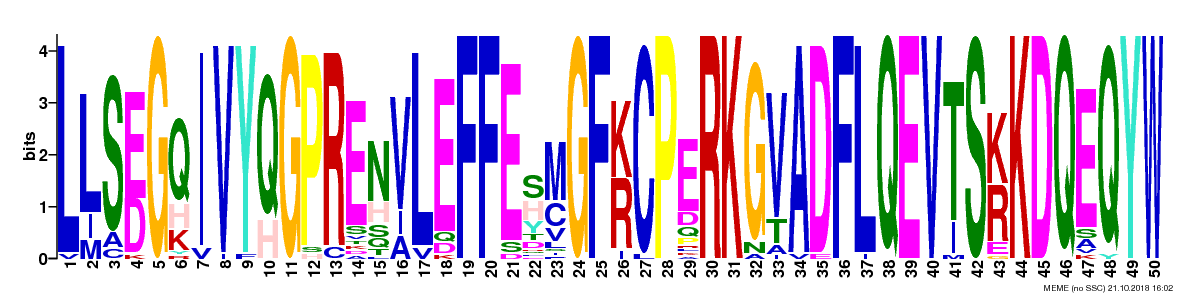 | LLSEGQIVYQGPRENVLEFFESMGFKCPERKGVADFLQEVTSKKDQEQYW | 3.4e-701 | 33 | 50 |
| 7 | 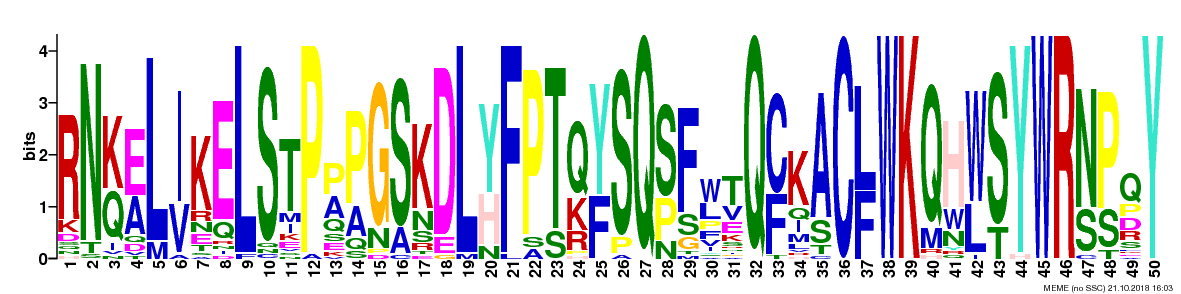 | RNKELIKELSTPPPGSKDLYFPTQYSQSFWTQCKACLWKQHWSYWRNPQY | 3.5e-693 | 33 | 50 |
| 8 | 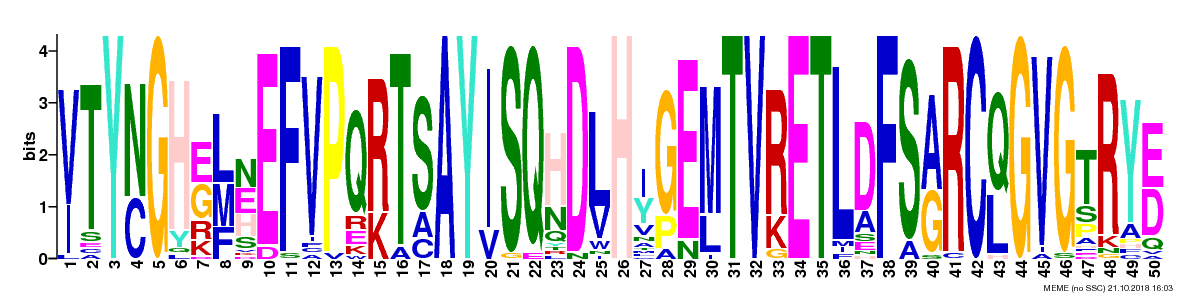 | VTYNGHELNEFVPQRTSAYISQHDLHIGEMTVRETLDFSARCQGVGTRYE | 4.8e-638 | 31 | 50 |
| 9 | 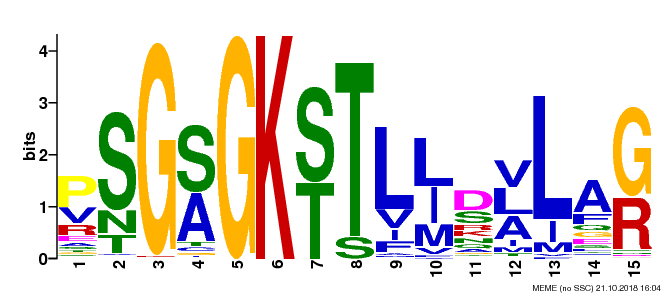 | PSGSGKSTLJDVLAG | 1.1e-631 | 182 | 15 |
| 10 | 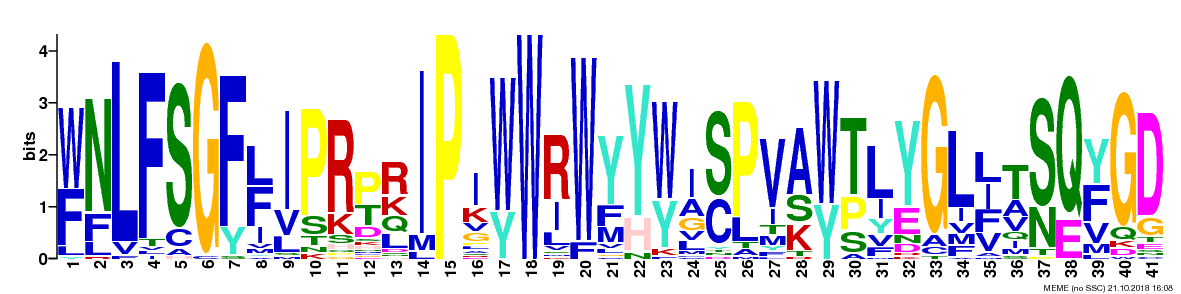 | WNLFSGFLIPRPRIPIWWRWYYWISPVAWTLYGLLTSQYGD | 5.4e-611 | 45 | 41 |
| 11 | 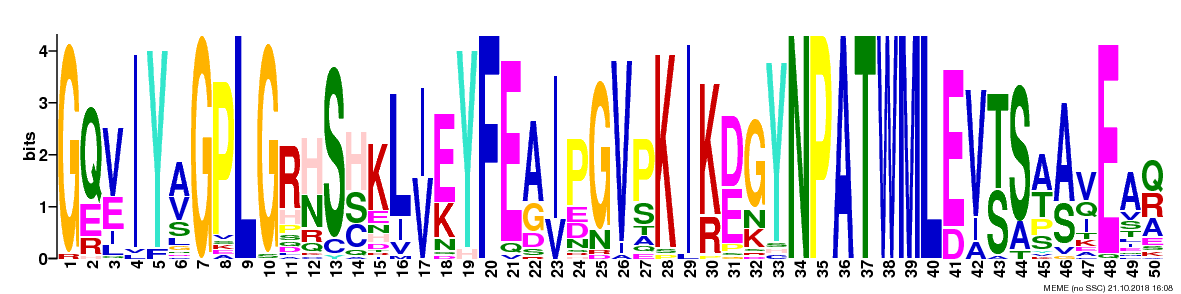 | GQVIYAGPLGRHSHKLIEYFEAIPGVPKIKDGYNPATWMLEVTSAAVEAQ | 2.8e-665 | 36 | 50 |
| 12 | 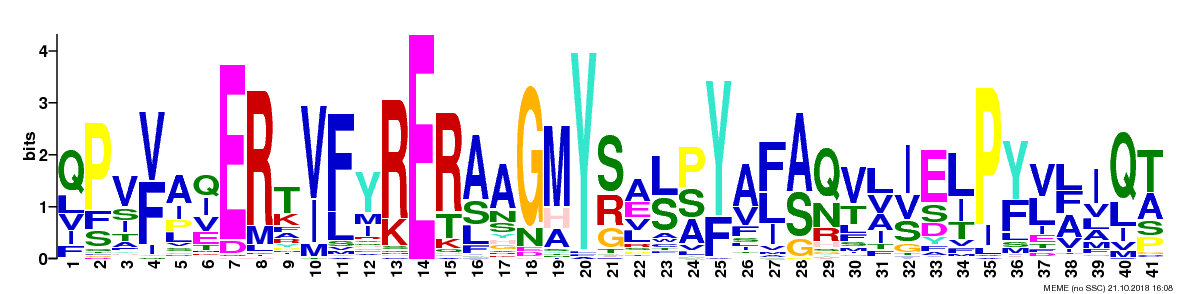 | QPVVAQERTVFYRERAAGMYSALPYAFAQVLIEJPYVLIQT | 2.8e-642 | 70 | 41 |
| 13 | 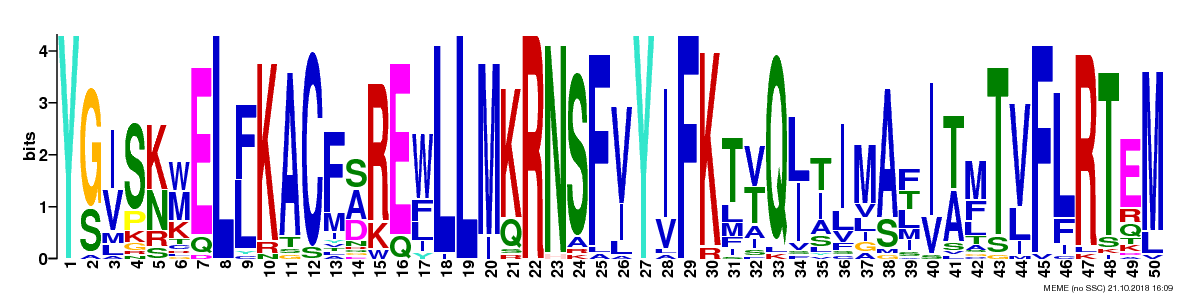 | YGISKMELFKACFSREWLLMKRNSFVYIFKTVQJTIMAFITMTVFLRTEM | 2.3e-530 | 33 | 50 |
| 14 | 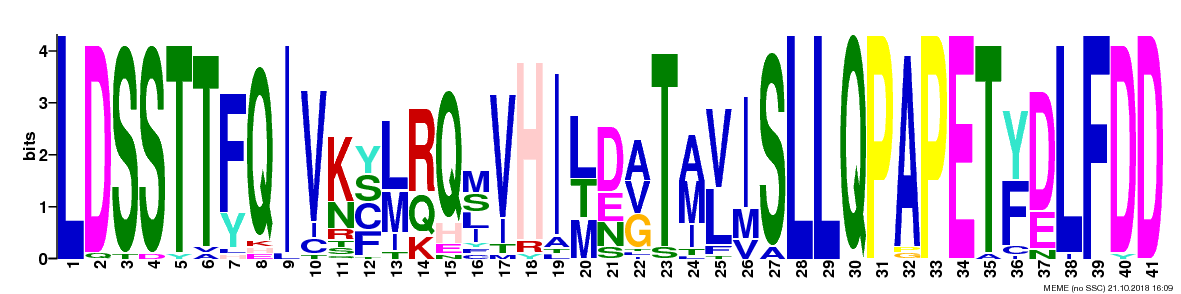 | LDSSTTFQIVKYLRQMVHILDATAVISLLQPAPETYDLFDD | 2.3e-470 | 32 | 41 |
| 15 | 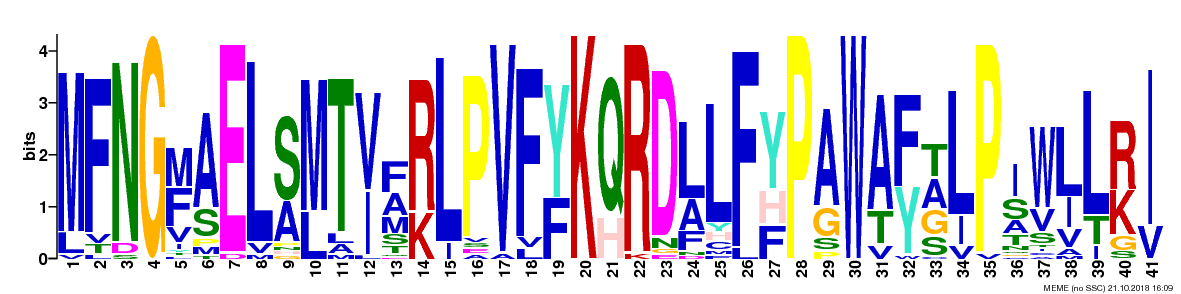 | MFNGMAELSMTVFRLPVFYKQRDLLFYPAWAFTLPIWLLRI | 5.1e-476 | 34 | 41 |
| 16 | 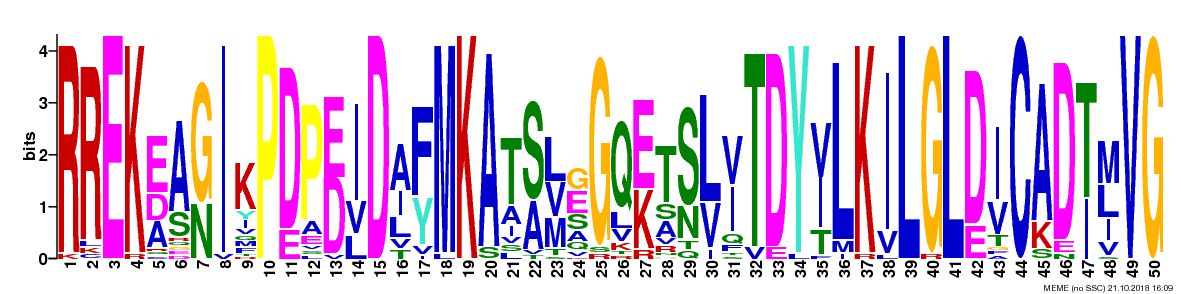 | RREKEAGIKPDPEIDAFMKATSLEGQETSLVTDYVLKILGLDICADTMVG | 9.1e-522 | 32 | 50 |
| 17 | 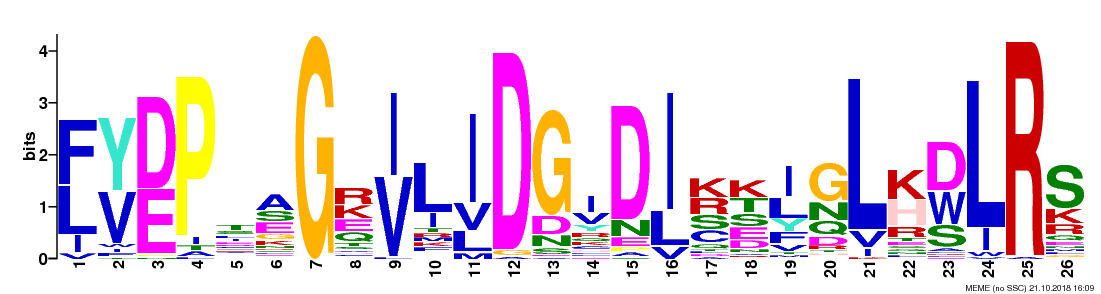 | FYDPTAGRILIDGIDIKKJGLKDLRS | 2.6e-336 | 62 | 26 |
| 18 | 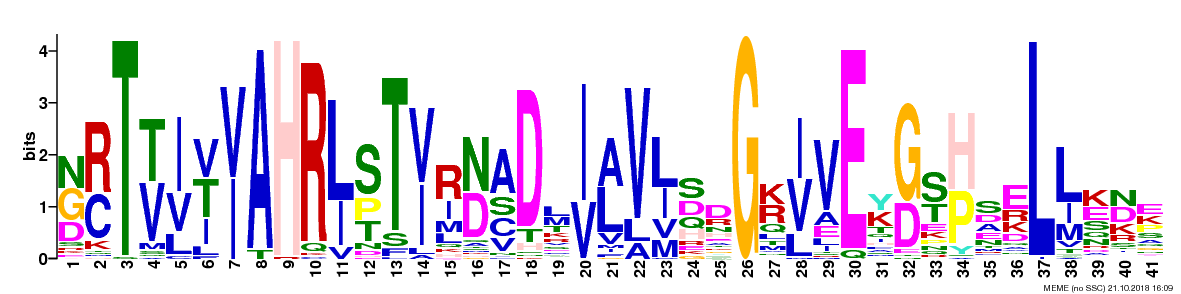 | NRTTIVVAHRLSTVRBADLIAVJSDGKIVEYGSHSELLKNE | 2.8e-435 | 60 | 41 |
| 19 | 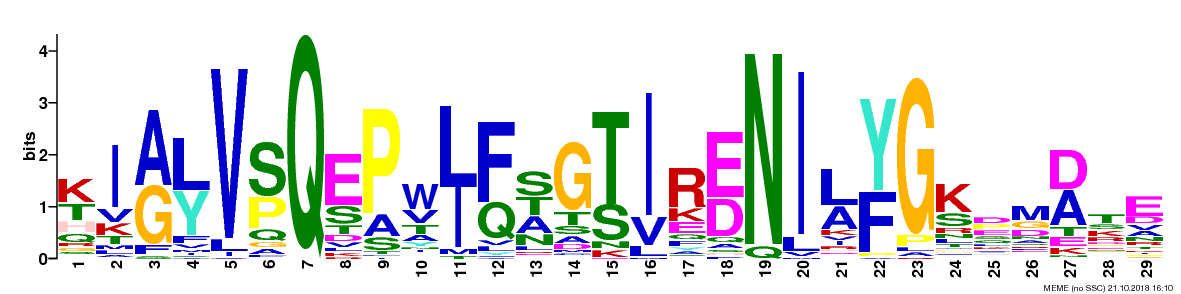 | KIALVSQEPWJFSGTIRENILYGKDMDTE | 4.3e-329 | 73 | 29 |
| 20 | 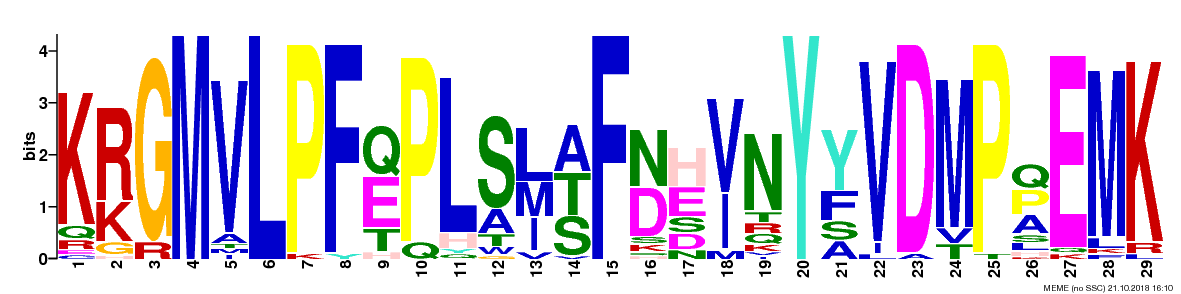 | KRGMVLPFZPLSLAFBHVNYYVDMPPEMK | 1.9e-314 | 34 | 29 |

Supplemental Table 2-2 Motifs information of ABC transporter family members in *Pyrus communis*

| Motif | LOGO | Sequence | E-value | Sites | Width |
| --- | --- | --- | --- | --- | --- |
| 1 | 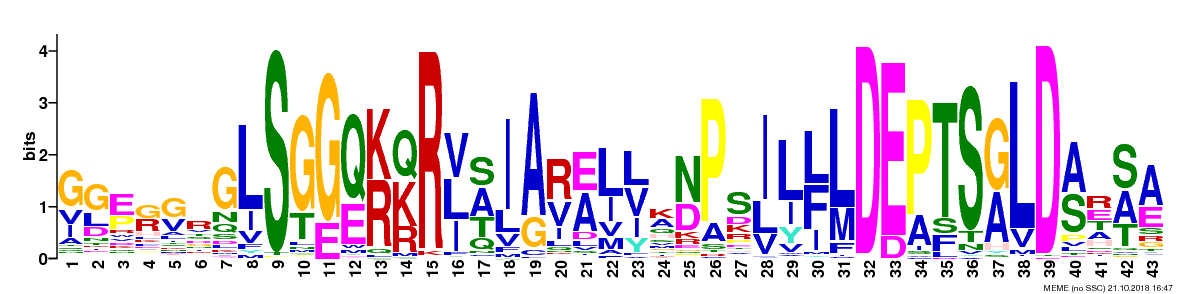 | GGEGGRGLSGGQKQRVSIAREJLKBPSILLLDEPTSGLDARSA | 2.9e-1621 | 158 | 43 |
| 2 | 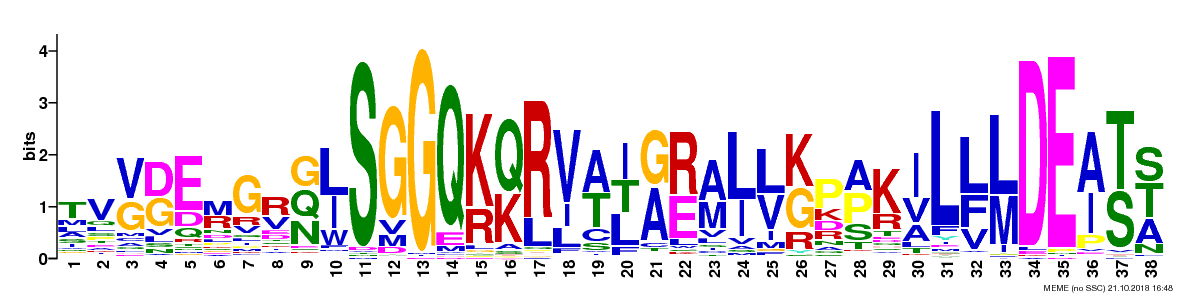 | TVVDEMGRGJSGGQKQRVAIGRAJLKPPKILLLDEATT | 1.1e-718 | 88 | 38 |
| 3 | 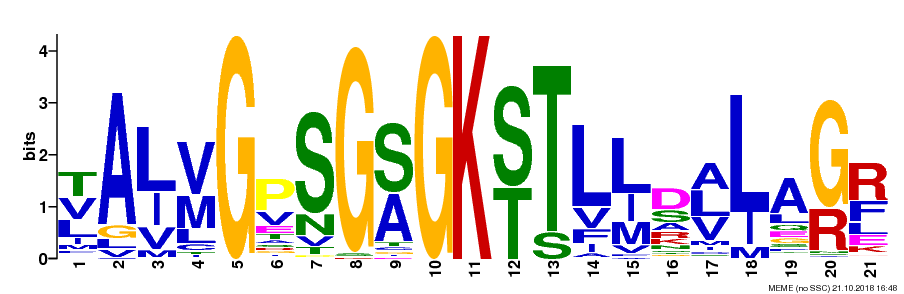 | TAJVGPSGSGKSTLJDALAGR | 1.9e-709 | 163 | 21 |
| 4 | 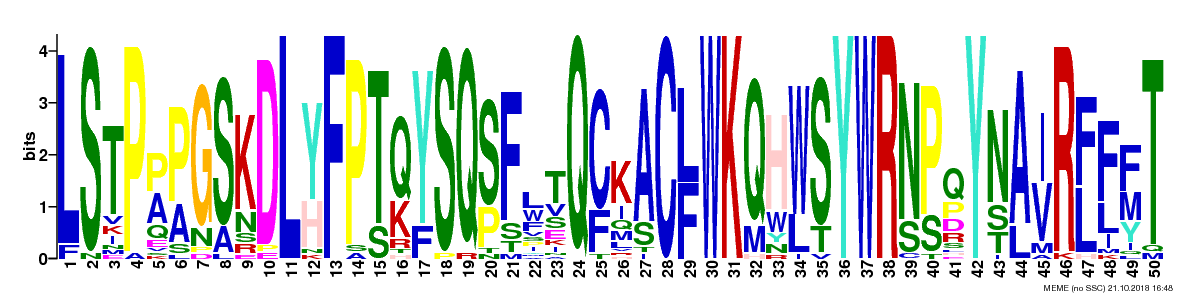 | LSTPPPGSKDLYFPTQYSQSFLTQCKACLWKQHWSYWRNPQYNAIRFFFT | 2.6e-622 | 27 | 50 |
| 5 | 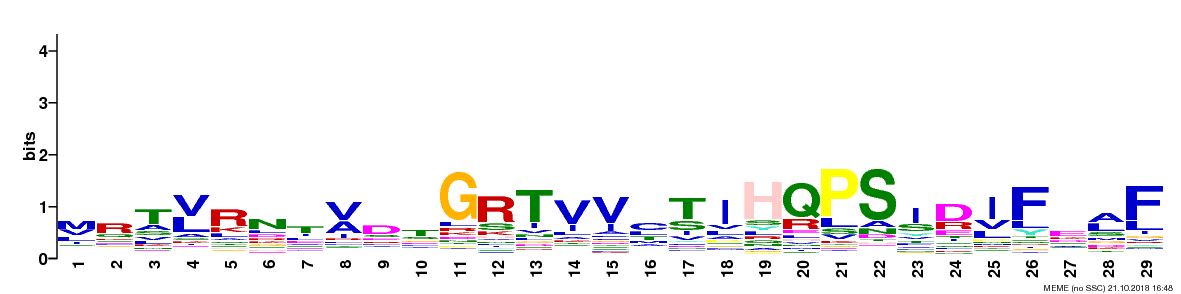 | MRTVRNTVDTGRTVVCTIHQPSIDIFEAF | 4.4e-519 | 171 | 29 |
| 6 | 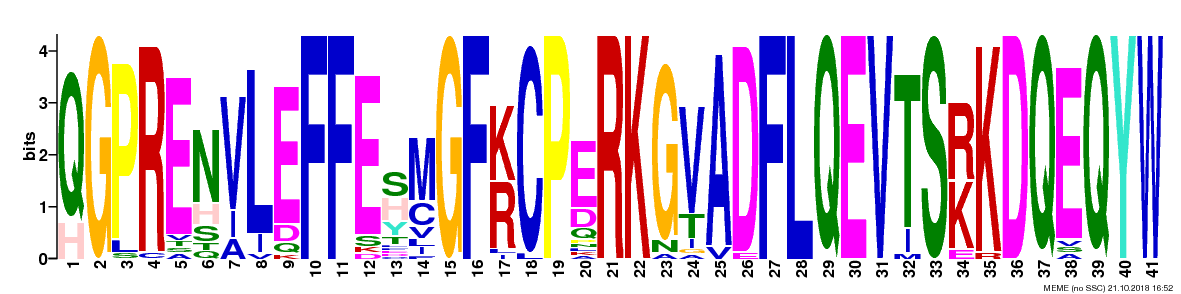 | QGPRENVLEFFESMGFKCPERKGVADFLQEVTSRKDQEQYW | 1.7e-604 | 30 | 41 |
| 7 | 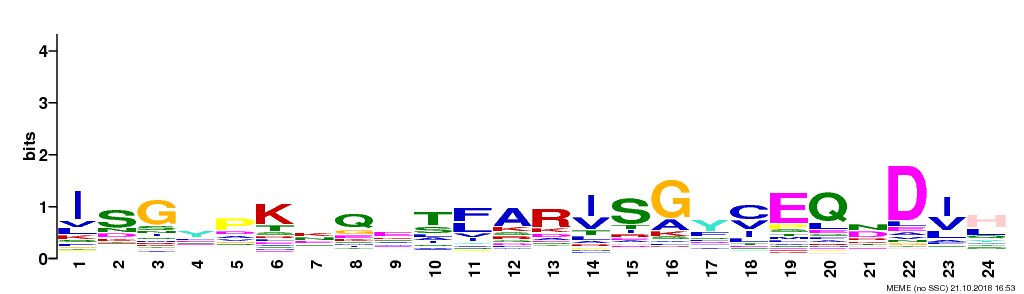 | ISGYPKKQETFARISGYCEQBDIH | 1.7e-457 | 160 | 24 |
| 8 | 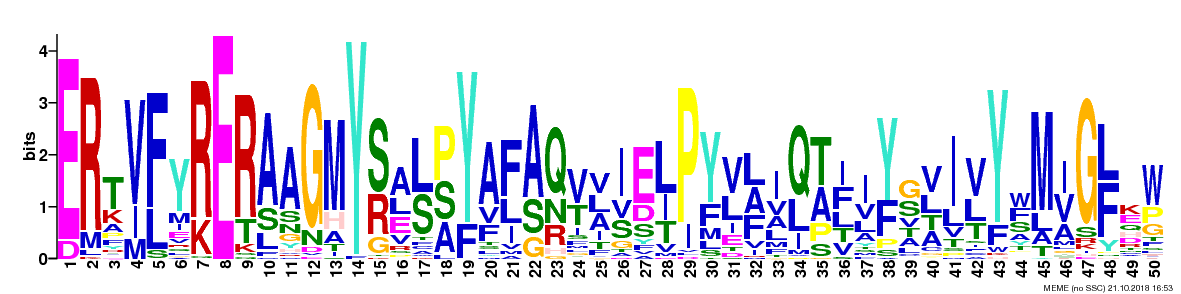 | ERTVFYRERAAGMYSALPYAFAQVVIEJPYVLIQTIIYGVIVYWMIGLKW | 3.9e-689 | 53 | 50 |
| 9 | 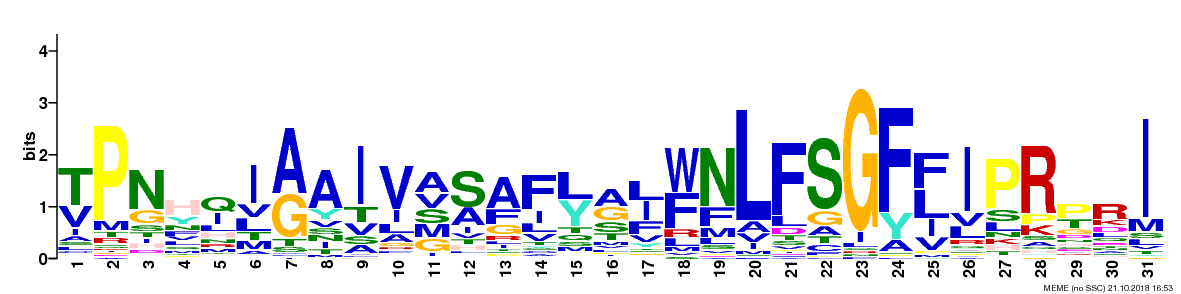 | TPNHQIAAIVASAFLALWNLFSGFFIPRPRI | 7.2e-461 | 62 | 31 |
| 10 | 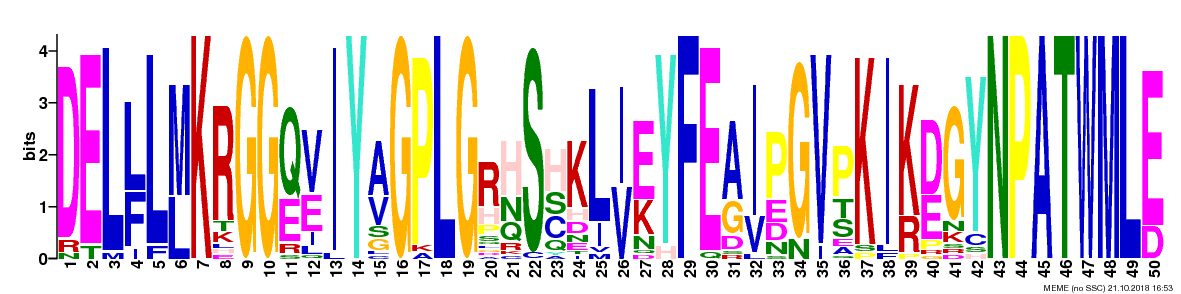 | DELLLMKRGGZVIYAGPLGRHSHKLIEYFEAIPGVPKIKDGYNPATWMLE | 4.2e-637 | 28 | 50 |
| 11 | 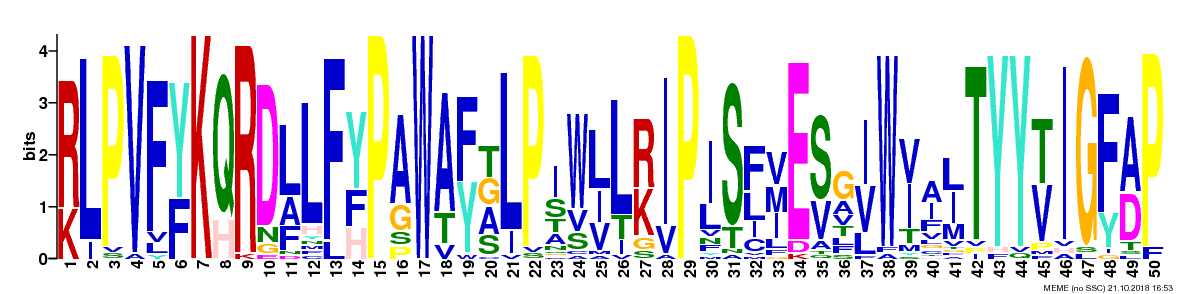 | RLPVFYKQRDLLFYPAWAFTLPIWJLRIPISFVESGIWIAJTYYTIGFAP | 1.6e-611 | 32 | 50 |
| 12 | 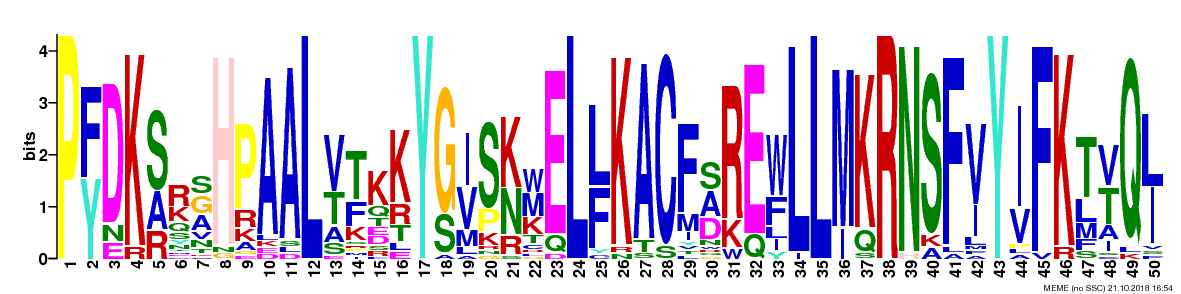 | PFDKSRSHPAALVTKKYGISKMELLKACFSREWLLMKRNSFVYIFKTVQJ | 2.5e-558 | 30 | 50 |
| 13 | 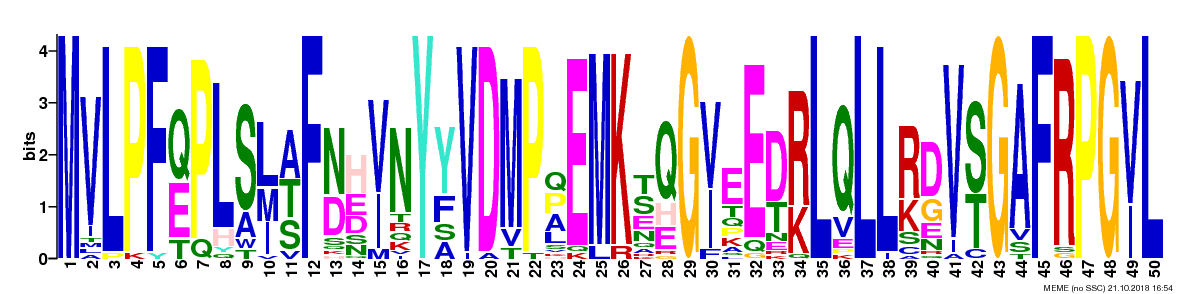 | MVLPFZPLSLAFBHVNYYVDMPPEMKSQGVEEDRLQLLRDVSGAFRPGVL | 9.6e-561 | 29 | 50 |
| 14 | 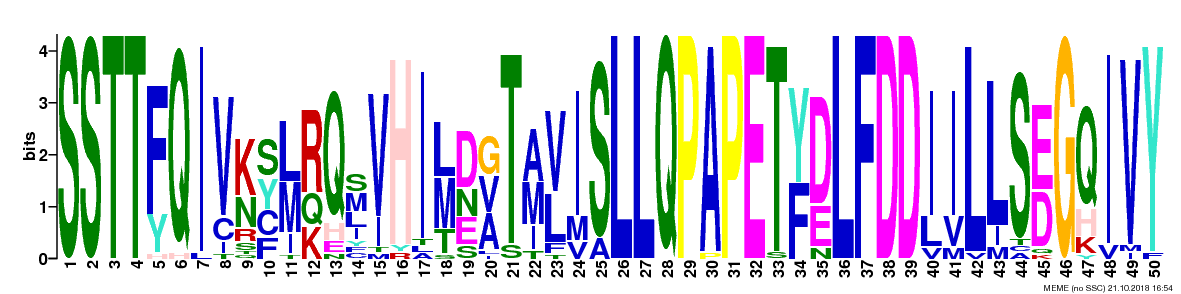 | SSTTFQIVKSLRQMVHILDGTAVISLLQPAPETYDLFDDIILLSEGQIVY | 3.7e-532 | 27 | 50 |
| 15 | 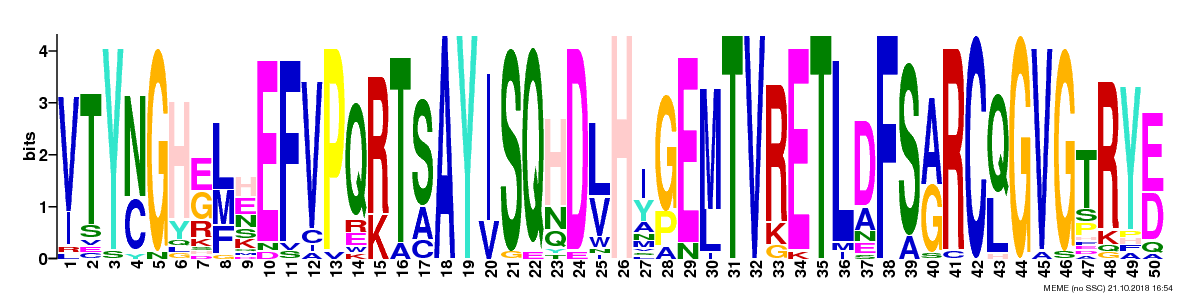 | VTYNGHELHEFVPQRTSAYISQHDLHIGEMTVRETLDFSARCQGVGTRYE | 1.2e-478 | 24 | 50 |
| 16 | 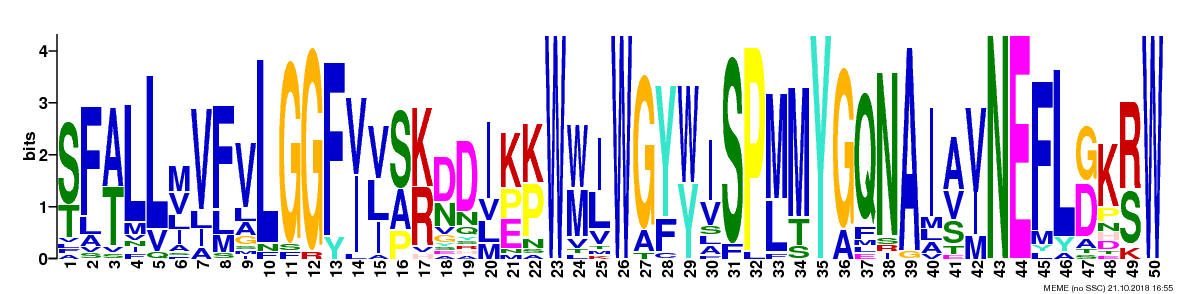 | SFALLMVFVLGGFVVSKBDIKPWWIWGYWISPMMYGQNAIAVNEFLGKRW | 3.9e-503 | 25 | 50 |
| 17 | 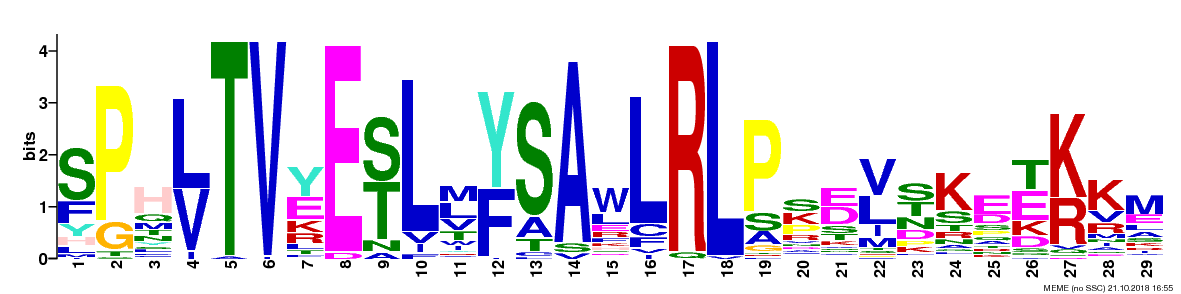 | SPHLTVYESLLYSAWLRLPSEVSKEEKKM | 2.0e-368 | 59 | 29 |
| 18 | 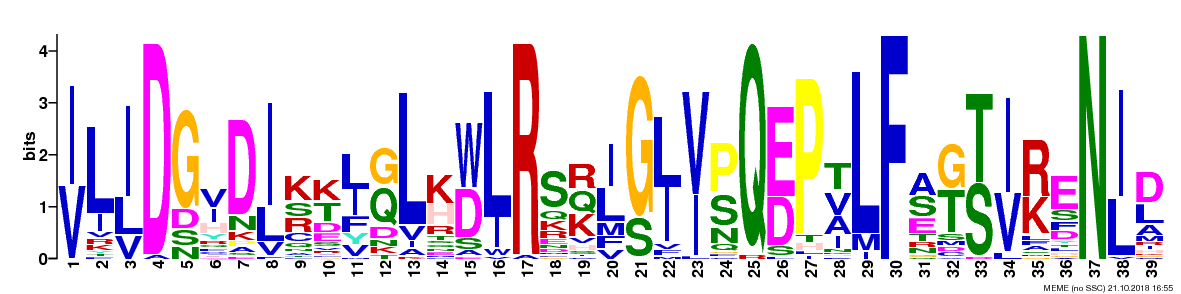 | ILIDGVDIKKJGLKWLRSRJGJVPQEPTLFAGTIRENJD | 5.6e-384 | 44 | 39 |
| 19 | 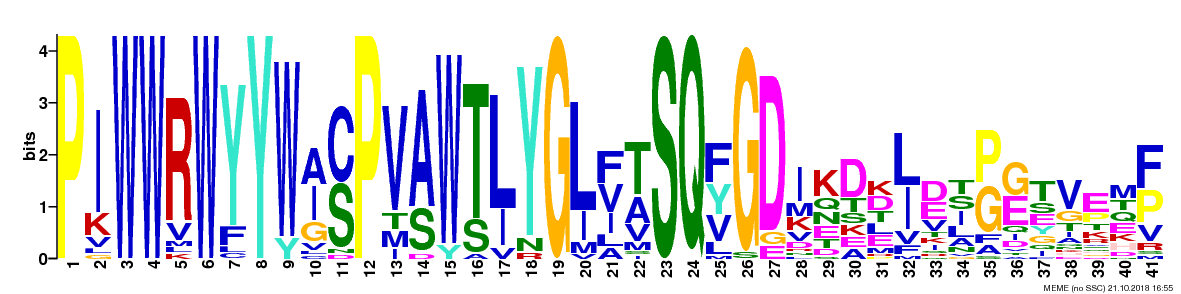 | PIWWRWYYWACPVAWTLYGLFTSQFGDIKDKJDIPETVEMF | 1.5e-379 | 26 | 41 |
| 20 | 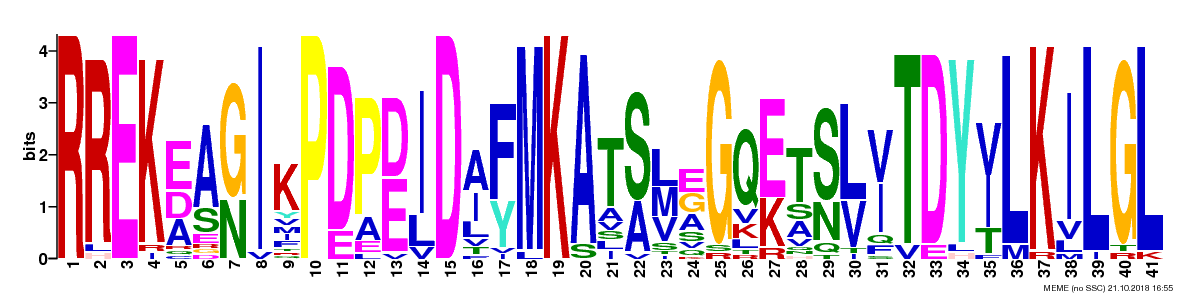 | RREKEAGIKPDPEIDAFMKATSLEGQETSLVTDYVLKILGL | 2.5e-344 | 26 | 41 |

Supplemental Table 2-3 Motifs information of ABC transporter family members in *Prunus persica*

| Motif | LOGO | Sequence | E-value | Sites | Width |
| --- | --- | --- | --- | --- | --- |
| 1 | 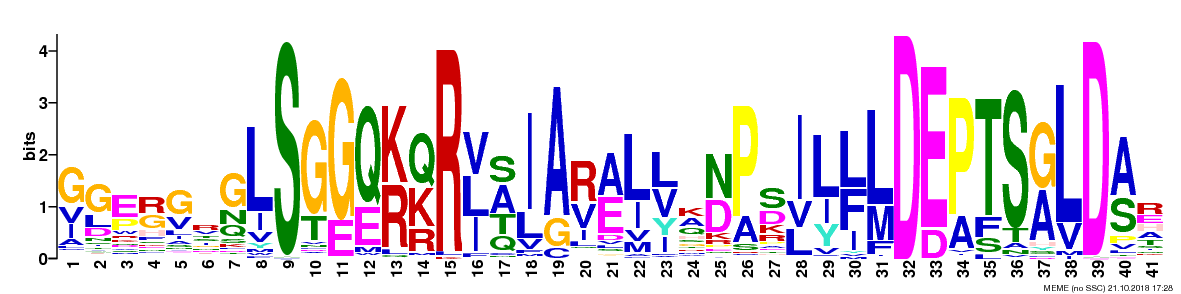 | GGERGRGLSGGQKQRVSIARALLKBPSILLLDEPTSGLDAR | 1.8e-1857 | 134 | 41 |
| 2 | 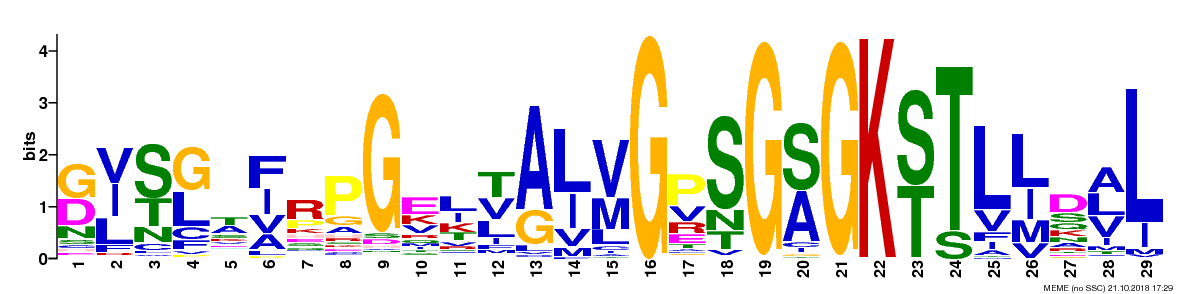 | GVSGTFRPGELTAJVGPSGSGKSTLJDAL | 4.5e-1098 | 136 | 29 |
| 3 | 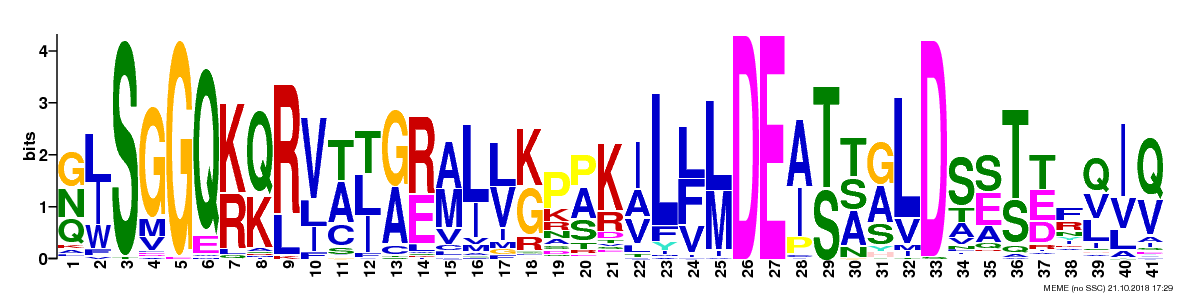 | GJSGGQKQRVATGRALLKPPKILLLDEATTGLDSSTTFQIQ | 2.3e-1011 | 74 | 41 |
| 4 | 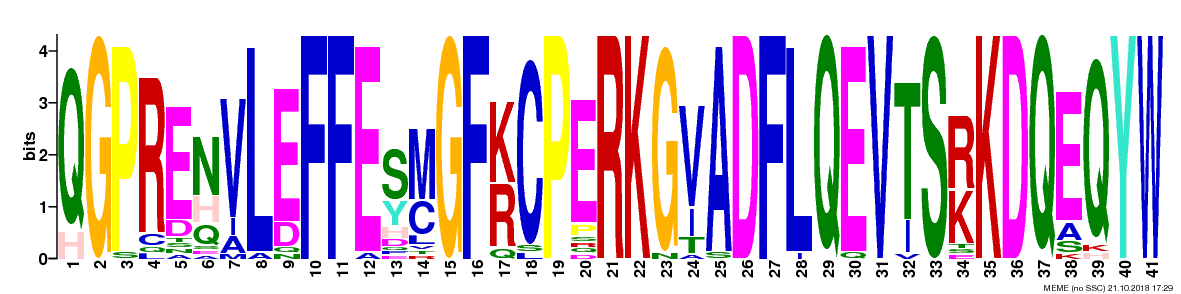 | QGPRENVLEFFESMGFKCPERKGVADFLQEVTSRKDQEQYW | 7.4e-535 | 27 | 41 |
| 5 | 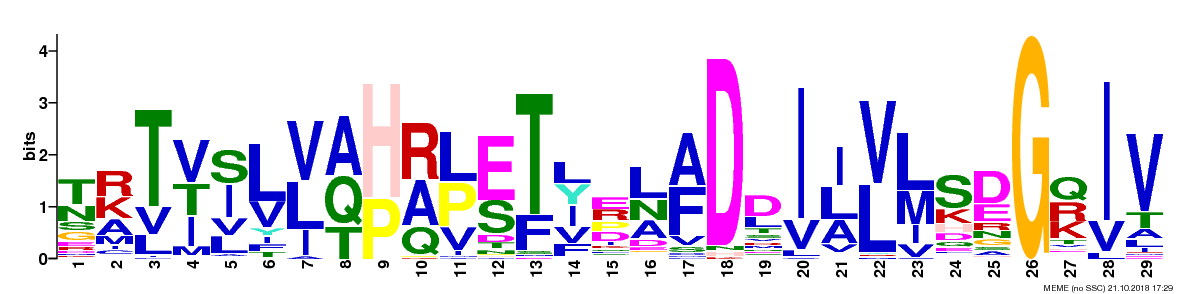 | TRTVILVAHRLETLELADDIIVLSDGQIV | 1.7e-582 | 74 | 29 |
| 6 | 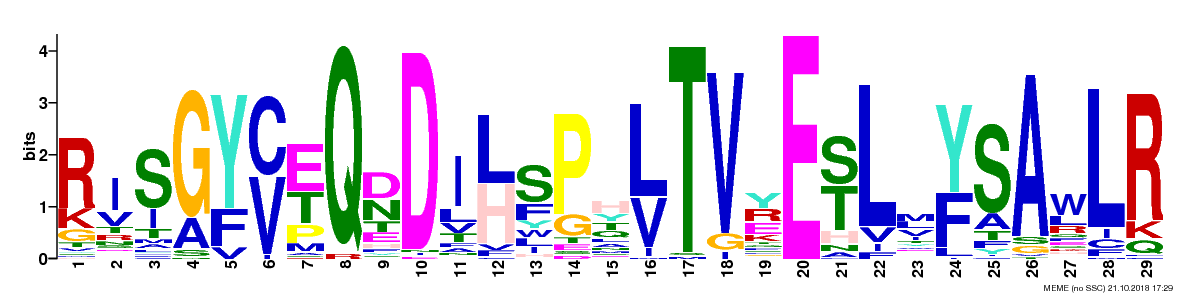 | RISGYCEQBDILSPHLTVYETLMYSAWLR | 1.4e-524 | 65 | 29 |
| 7 | 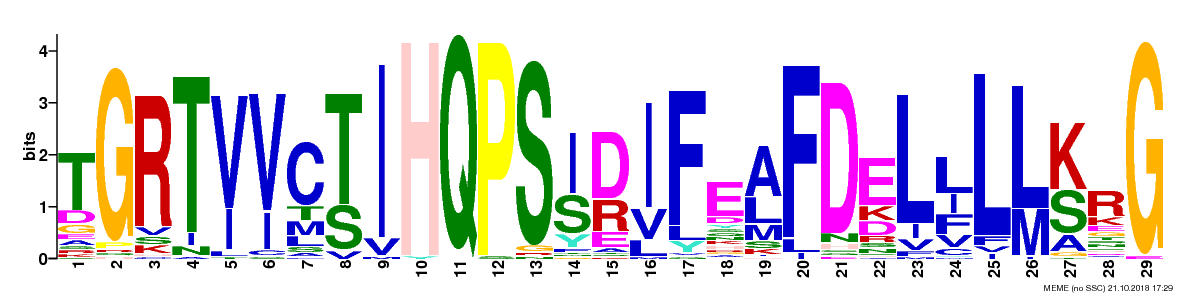 | TGRTVVCTIHQPSIDIFEAFDELLLLKRG | 1.3e-520 | 55 | 29 |
| 8 | 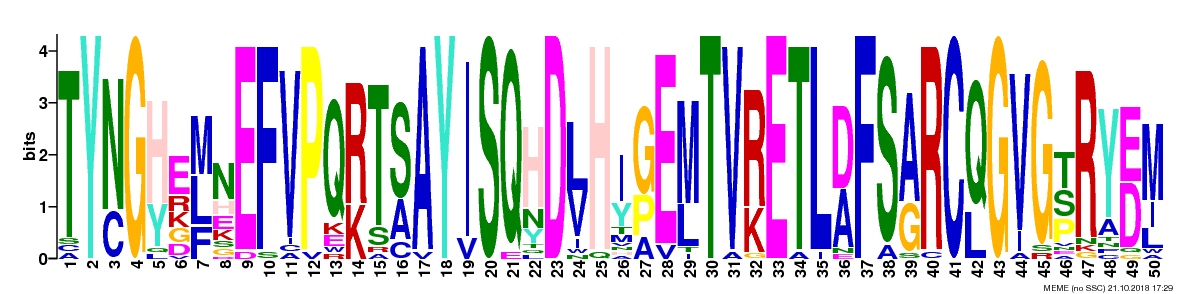 | TYNGHEMNEFVPQRTSAYISQHDLHIGEMTVRETLDFSARCQGVGTRYEM | 1.6e-530 | 26 | 50 |
| 9 | 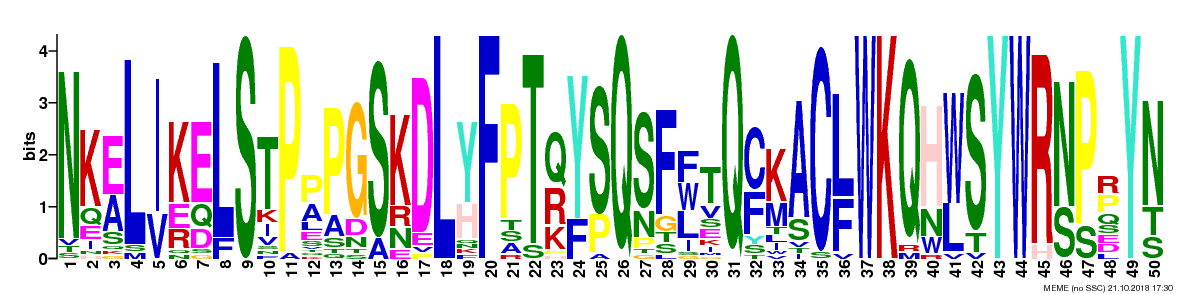 | NKELIKELSTPPPGSKDLYFPTQYSQSFFTQCKACLWKQHWSYWRNPRYN | 3.4e-530 | 27 | 50 |
| 10 | 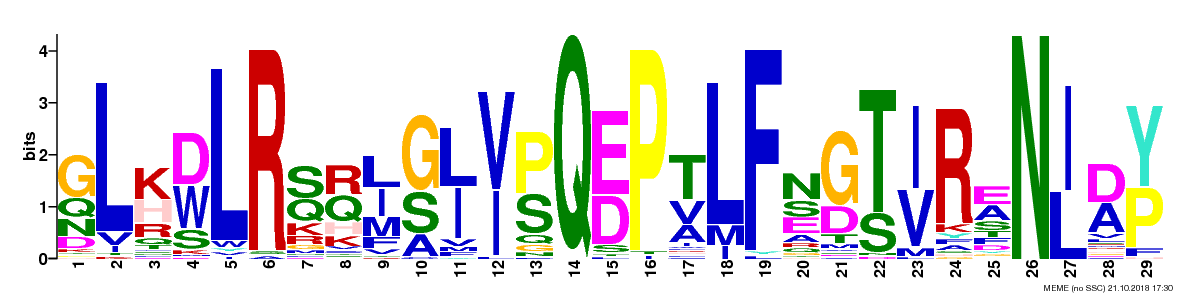 | GLKDLRSRLGJVPQEPTLFNGTIRENJDY | 4.1e-510 | 51 | 29 |
| 11 | 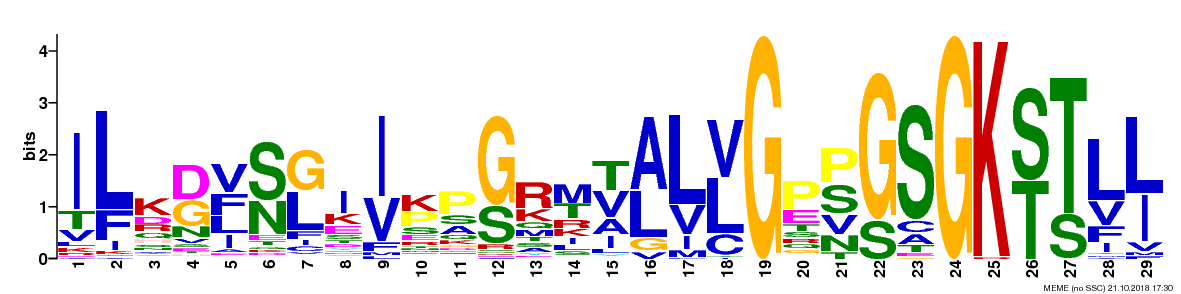 | ILKDVSGIIKPGRMTALVGPPGSGKSTLJ | 7.5e-514 | 72 | 29 |
| 12 | 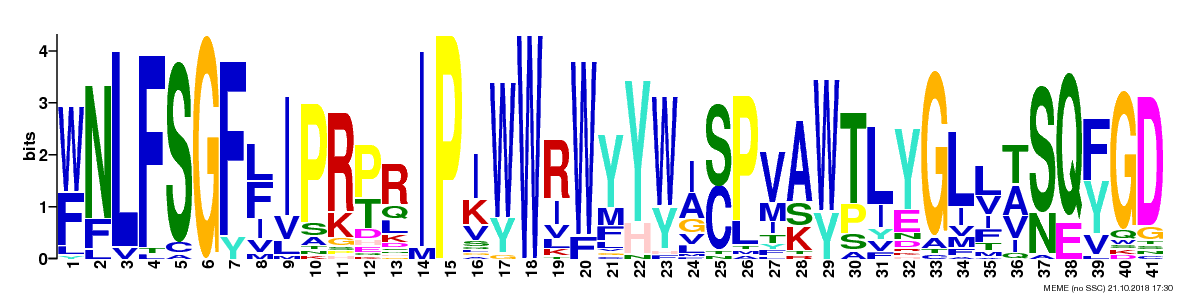 | WNLFSGFLIPRPRIPIWWRWYYWISPVAWTLYGLLTSQFGD | 4.0e-509 | 34 | 41 |
| 13 | 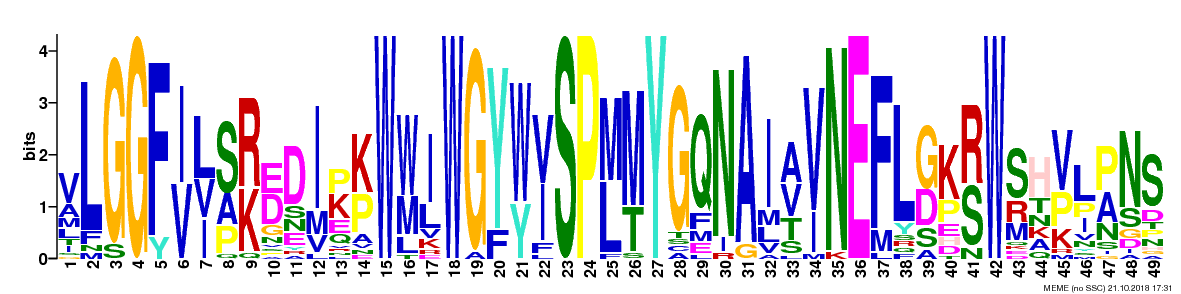 | VLGGFILSREDIKKWWIWGYWVSPMMYGQNAIAVNEFLGKRWSHVLPNS | 1.3e-436 | 25 | 49 |
| 14 | 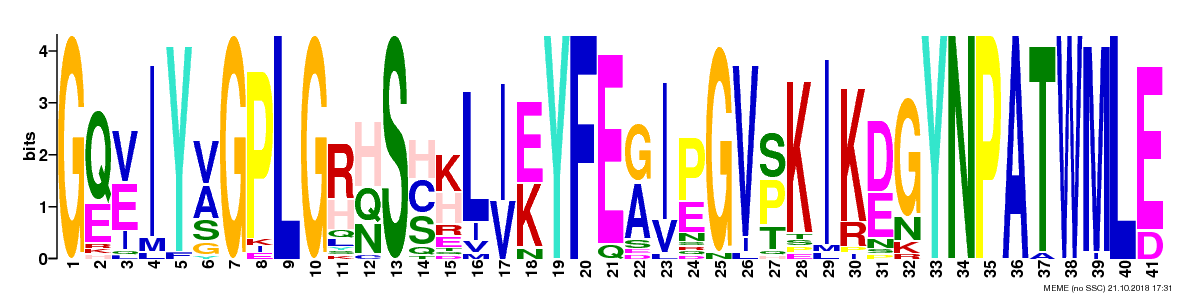 | GQVIYVGPLGRHSHKLIEYFEGIPGVPKIKDGYNPATWMLE | 5.5e-433 | 27 | 41 |
| 15 | 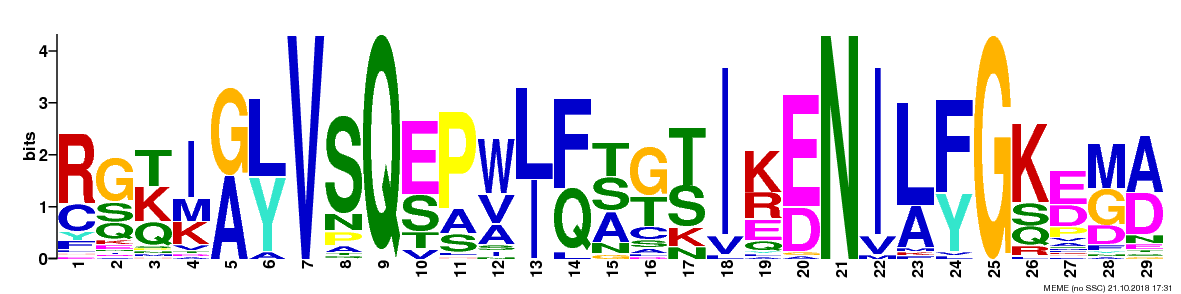 | RGTIGLVSQEPWJFSGTIKENILFGKEMA | 6.0e-413 | 41 | 29 |
| 16 | 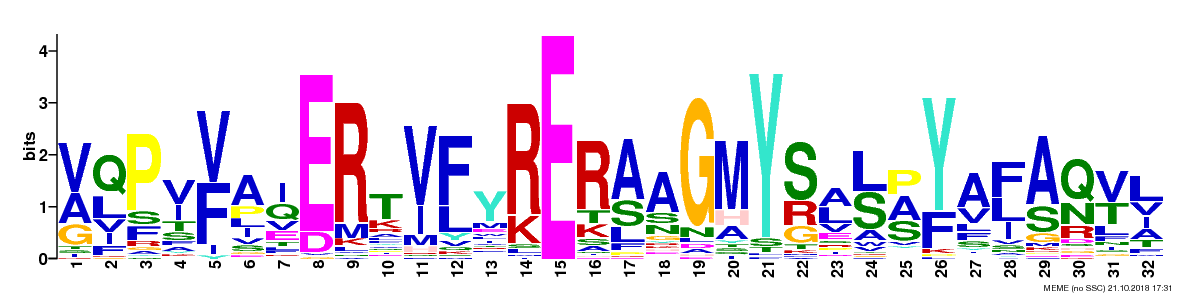 | VQPVVAIERTVFYRERAAGMYSALAYAFAQVL | 4.5e-349 | 60 | 32 |
| 17 | 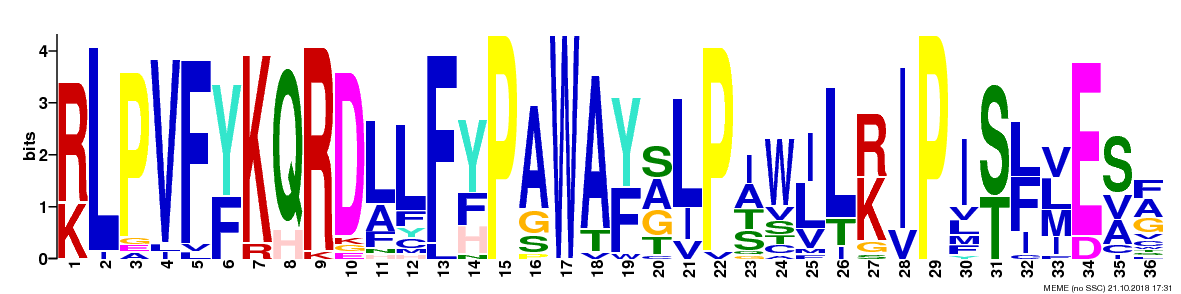 | RLPVFYKQRDLLFYPAWAYALPIWJLKIPISLLESA | 8.80E-306 | 25 | 36 |
| 18 | 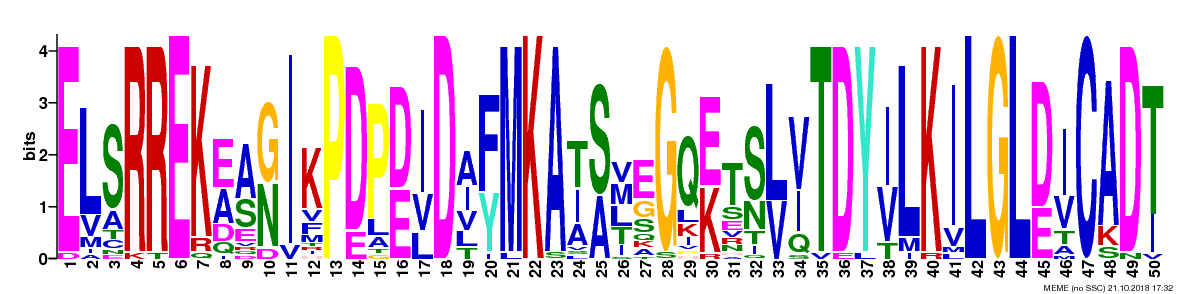 | ELSRREKEAGIKPDPDIDAFMKATSVEGQETSLVTDYILKILGLDICADT | 2.2e-452 | 27 | 50 |
| 19 | 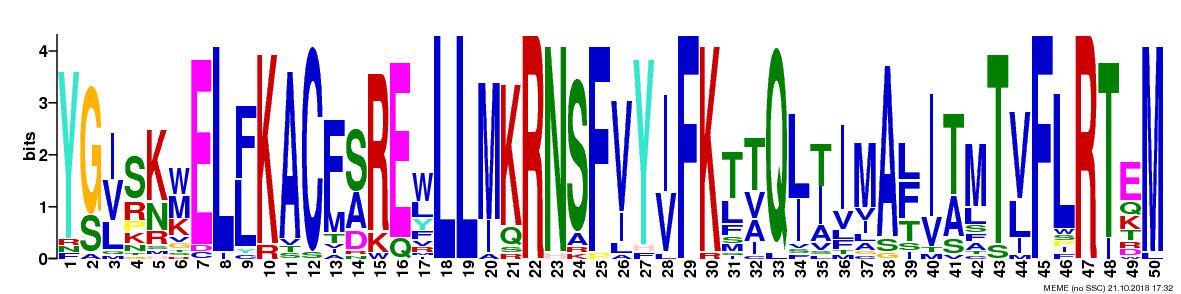 | YGISKWELFKACFSREWLLMKRNSFVYIFKTTQLTIMALITMTVFLRTEM | 4.3e-429 | 27 | 50 |
| 20 | 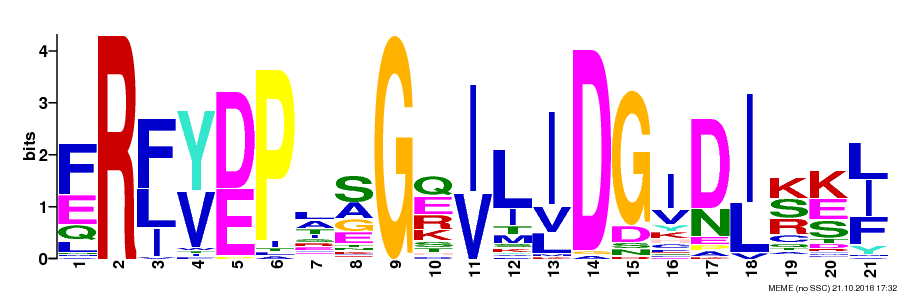 | FRFYDPASGQILIDGIDIKKL | 3.00E-268 | 50 | 21 |

Supplemental Table 2-4 Motifs information of ABC transporter family members in *Prunus avium*

| Motif | LOGO | Sequence | E-value | Sites | Width |
| --- | --- | --- | --- | --- | --- |
| 1 | 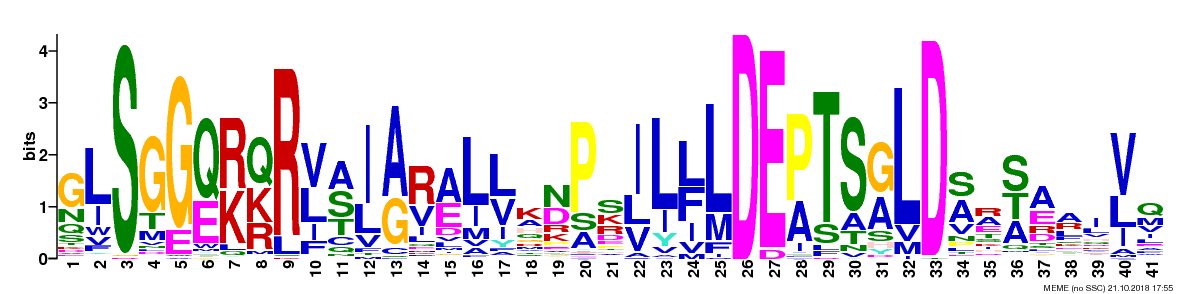 | GLSGGZRQRVAIARALLKBPSILLLDEPTSGLDSRSAAJVQ | 1.8e-1346 | 80 | 41 |
| 2 | 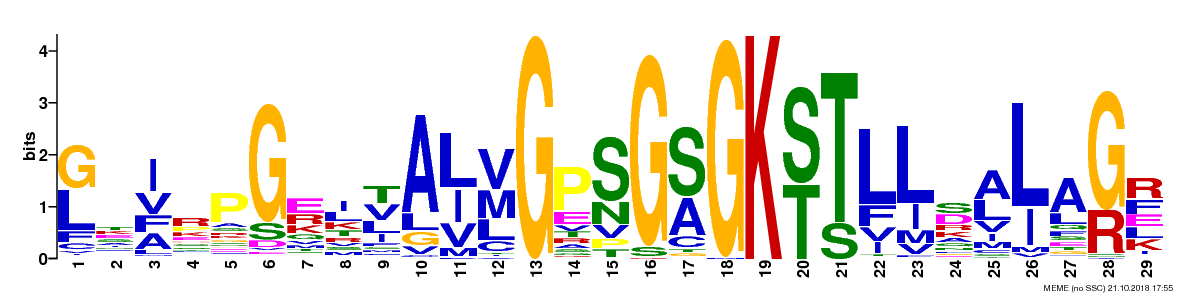 | GKIRPGEITAJVGPSGSGKSTLLSALAGR | 2.5e-1044 | 96 | 29 |
| 3 | 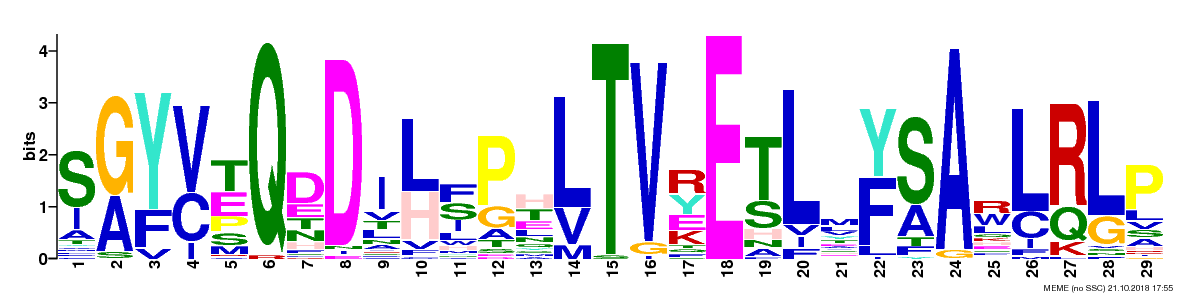 | SGYVTQDDILFPHLTVRETLMYSARLRLP | 4.1e-538 | 44 | 29 |
| 4 | 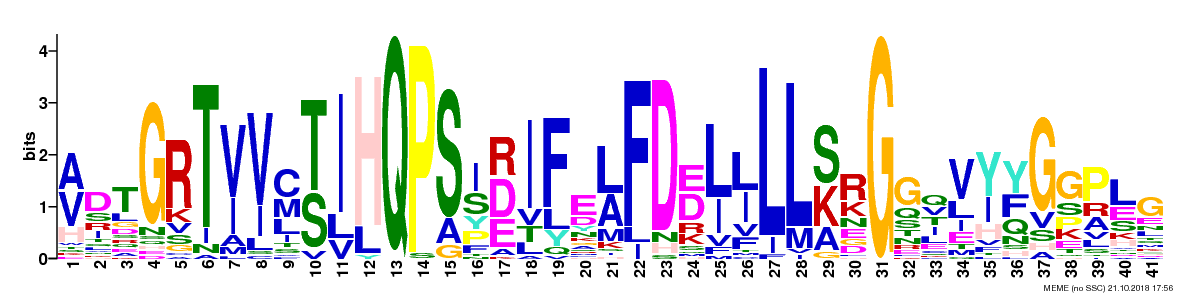 | ADTGRTVVCTIHQPSIDIFELFDELJLLSRGGQVYYGGPLG | 4.7e-467 | 33 | 41 |
| 5 | 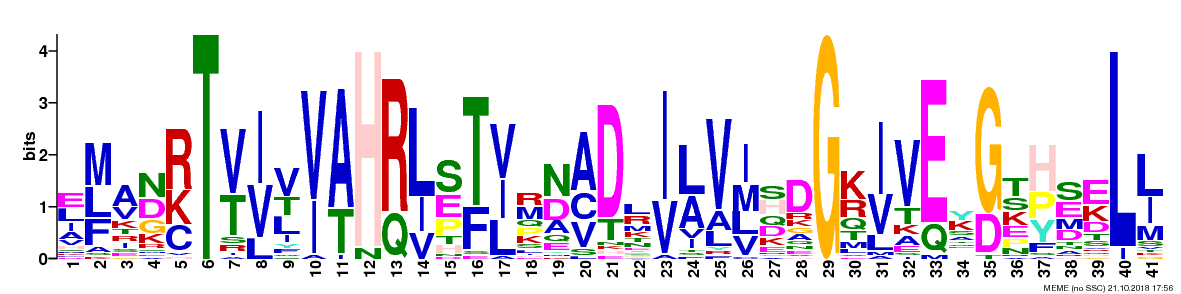 | EMANRTVIVVAHRLSTVRBADLILVISDGKIVEKGTHSELJ | 7.0e-472 | 34 | 41 |
| 6 | 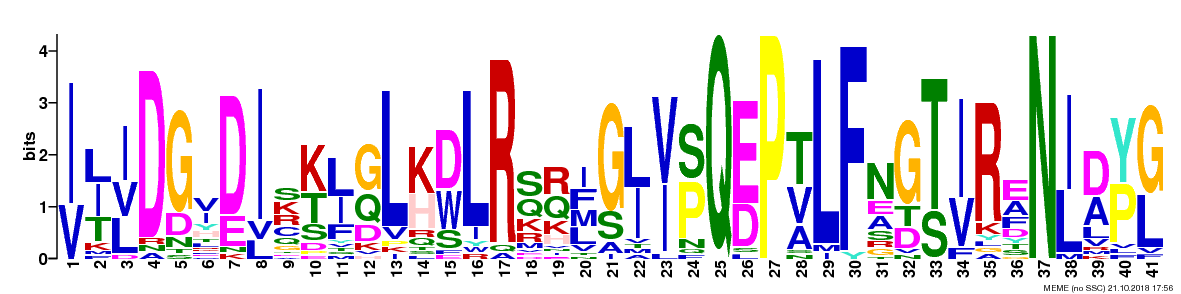 | ILIDGVDIKKJGLKDLRSRIGJVPQEPTLFNGTIRENJDYG | 5.6e-427 | 26 | 41 |
| 7 | 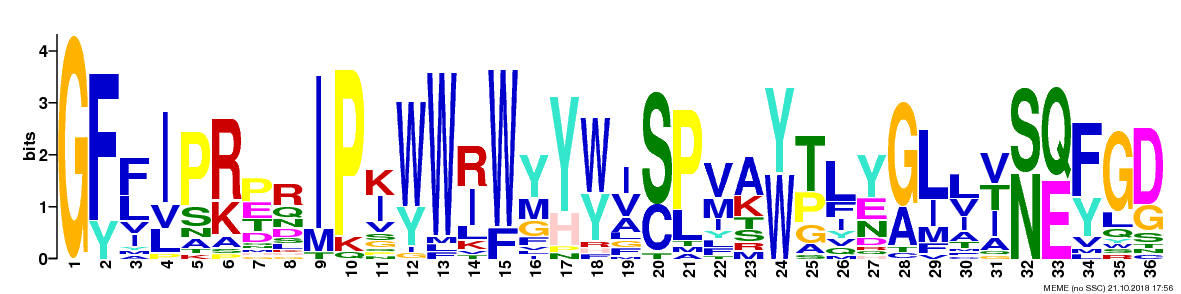 | GFFIPRPRIPKWWRWYYWISPVAYTLYGLLVNZFGD | 1.7e-361 | 24 | 36 |
| 8 | 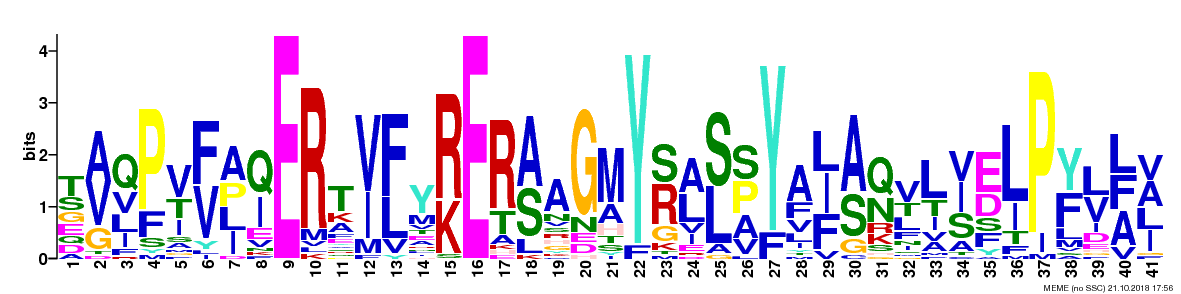 | TAQPVFAQERTVFYRERAAGMYRASSYALAQVLVELPYLLV | 4.7e-347 | 28 | 41 |
| 9 | 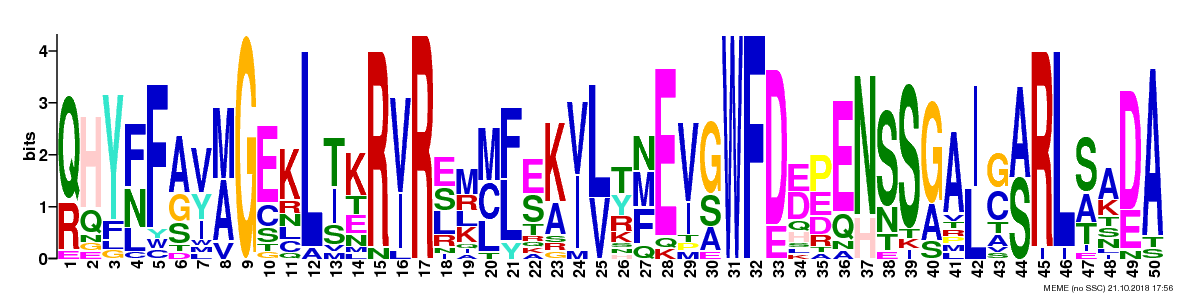 | QHYFFAVMGEKLTKRVREMMFEKILTFEVGWFDEPENSSGAJGARLSADA | 6.8e-341 | 17 | 50 |
| 10 | 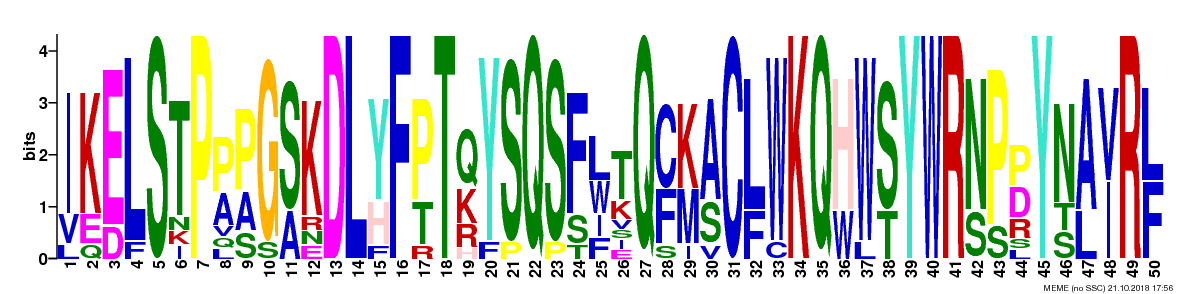 | IKELSTPPPGSKDLYFPTQYSQSFLTQCKACLWKQHWSYWRNPPYNAVRL | 7.0e-331 | 11 | 50 |
| 11 | 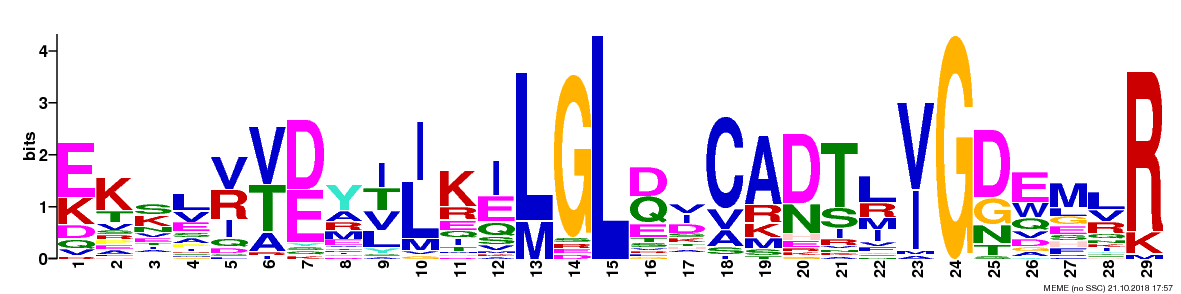 | EKKLVVDYIJKILGLDDCADTLVGDEMLR | 6.30E-249 | 34 | 29 |
| 12 | 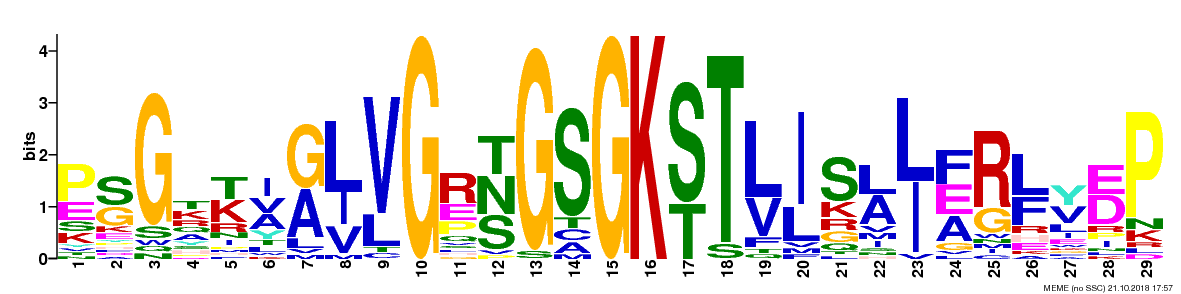 | PSGKKIGLVGRTGSGKSTLISLJFRLYEP | 8.00E-186 | 25 | 29 |
| 13 | 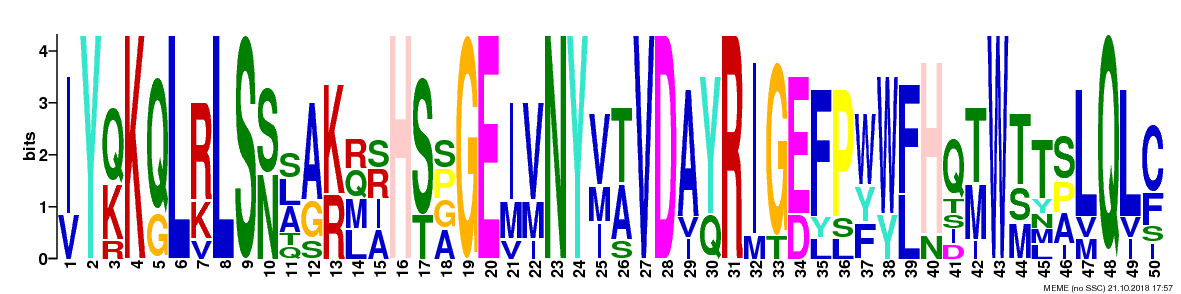 | IYQKQLRLSNAAKLAHSAGEIVNYVTVDAYRIGEFPWWFHQTWTTSLQLC | 2.00E-166 | 8 | 50 |
| 14 | 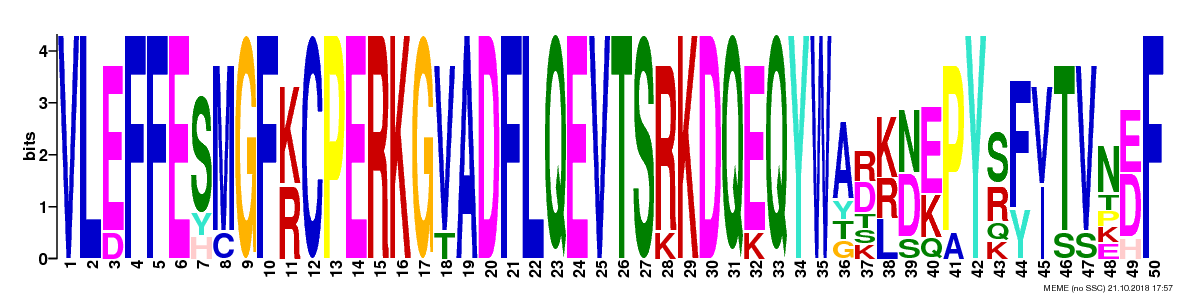 | VLEFFESMGFKCPERKGVADFLQEVTSRKDQEQYWARKBEPYSFVTVNDF | 9.90E-189 | 7 | 50 |
| 15 | 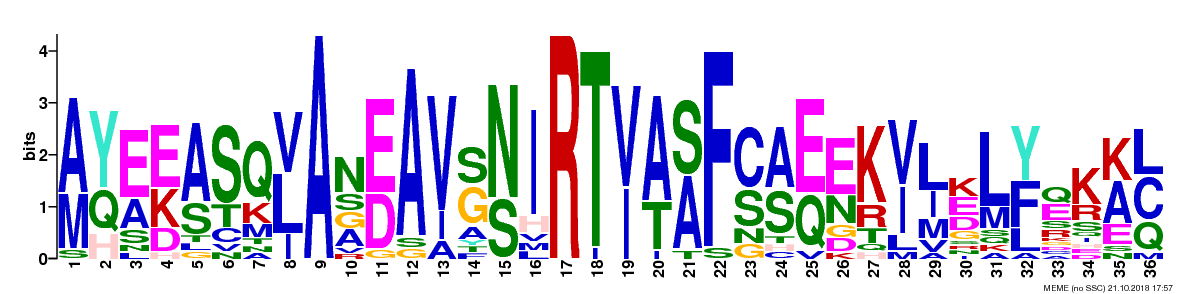 | AYEEASQVANEAVSNIRTVAAFCAZEKVJKLYEKKL | 1.40E-183 | 17 | 36 |
| 16 | 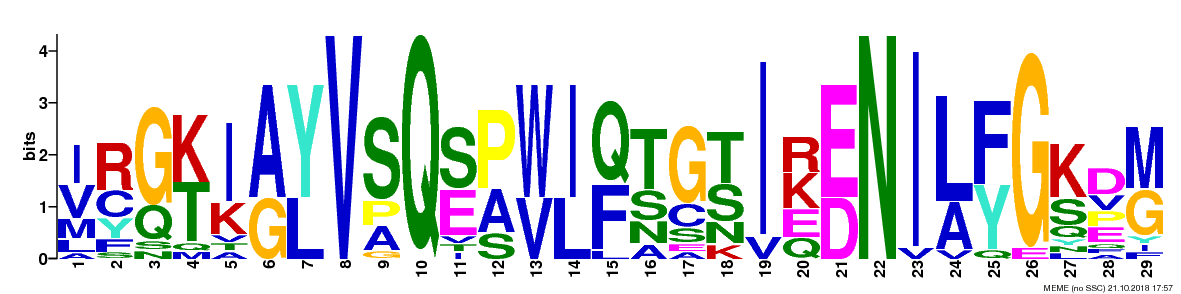 | IRGKIAYVSQSPWJQTGTIKENILFGKDM | 1.10E-185 | 17 | 29 |
| 17 | 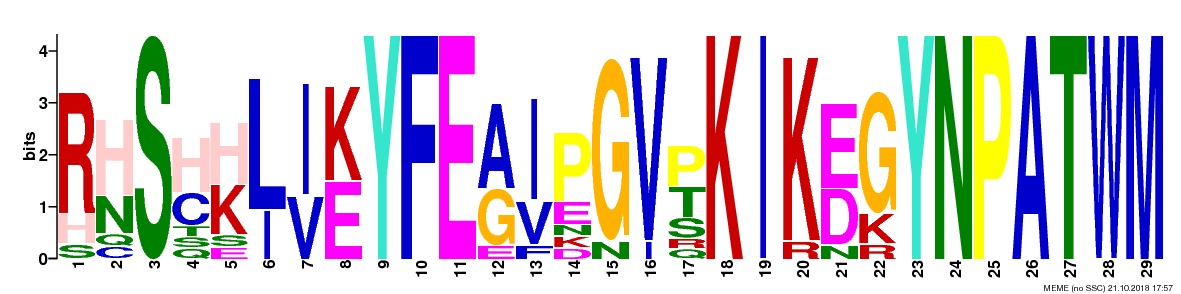 | RHSHHLIKYFEAIPGVPKIKEGYNPATWM | 1.40E-176 | 11 | 29 |
| 18 | 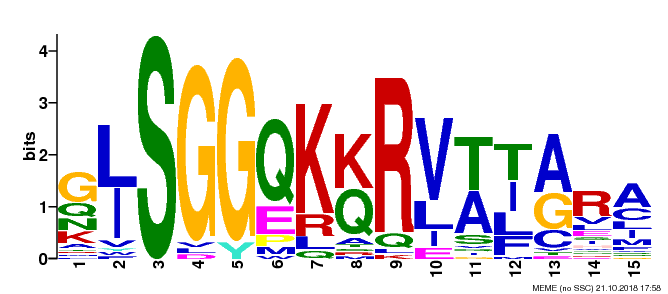 | GJSGGQKKRVTTARA | 2.80E-118 | 34 | 15 |
| 19 | 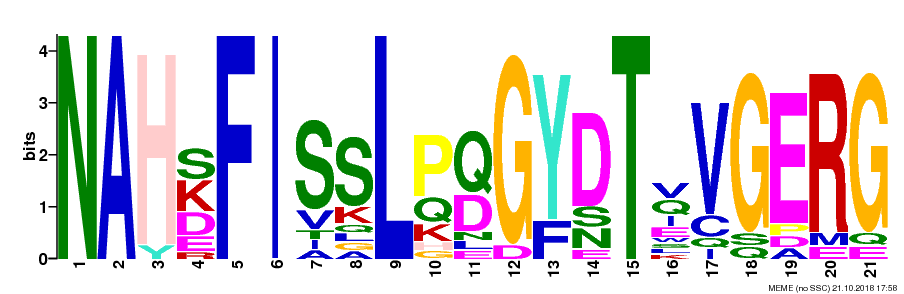 | NAHKFISSLPQGYDTVVGERG | 2.30E-116 | 14 | 21 |
| 20 | 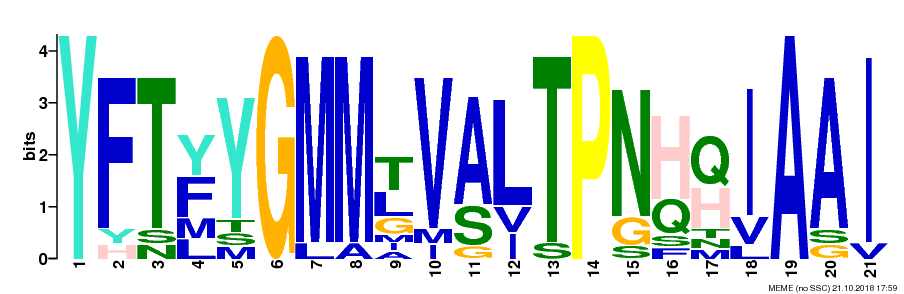 | YFTYYGMMTVALTPNHQIAAI | 2.80E-106 | 12 | 21 |

Supplemental Table 2-5 Motifs information of ABC transporter family members in *Prunus dulcis*

| Motif | LOGO | Sequence | E-value | Sites | Width |
| --- | --- | --- | --- | --- | --- |
| 1 | 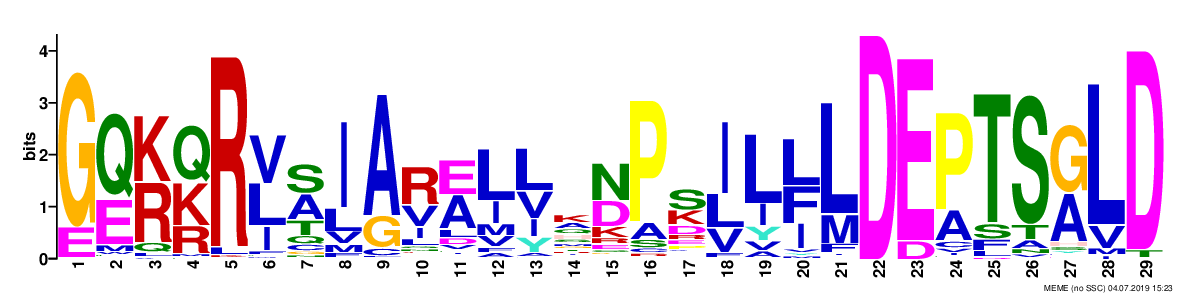 | GZKQRVSIARELLKBPSILLLDEPTSGLD | 1.5e-1402 | 128 | 29 |
| 2 | 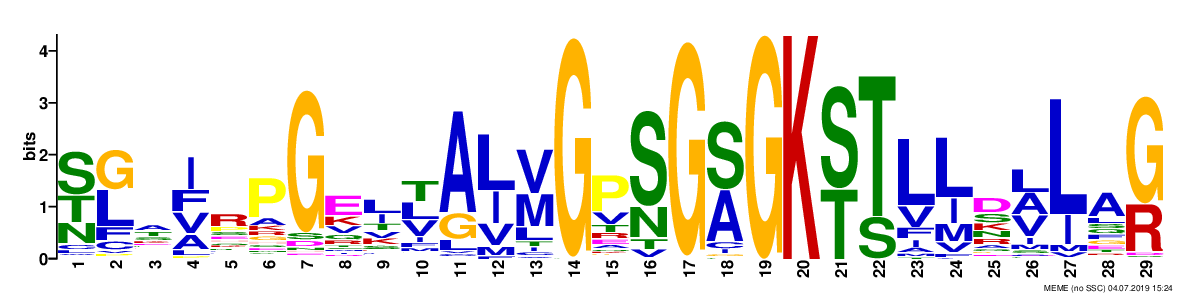 | SGAIRPGELTAJVGPSGSGKSTLLDLLAG | 3.9e-1150 | 133 | 29 |
| 3 | 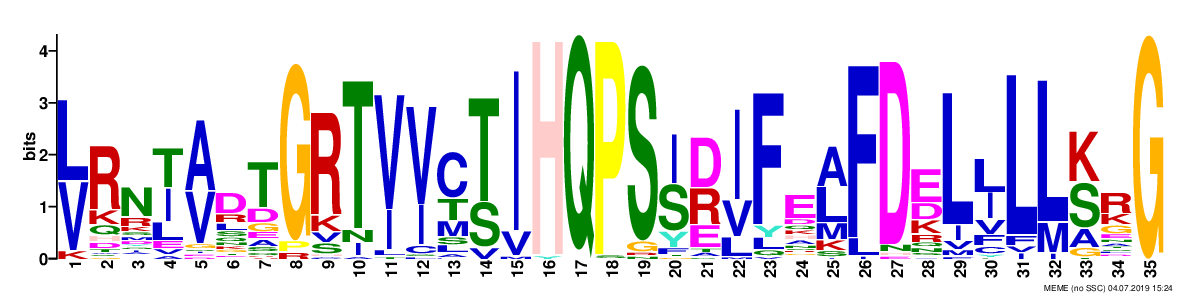 | LRNTADTGRTVVCTIHQPSIDIFEAFDELJLLKRG | 1.1e-644 | 53 | 35 |
| 4 | 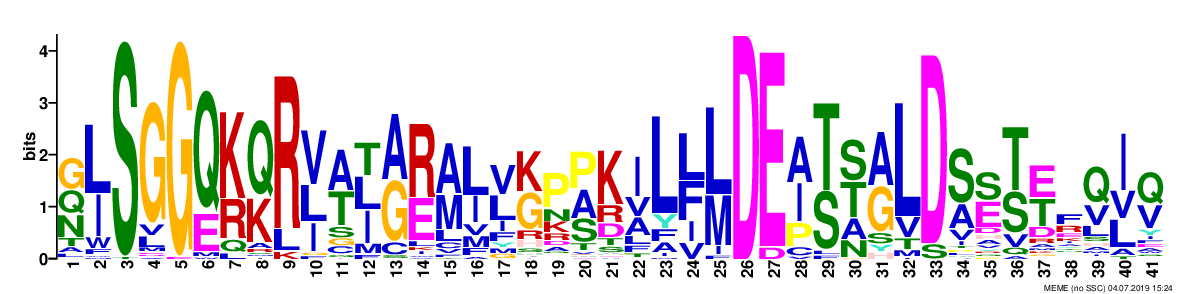 | GJSGGQKQRVALARAJVKPPKILLLDEATSALDSSTEFQIQ | 3.7e-759 | 66 | 41 |
| 5 | 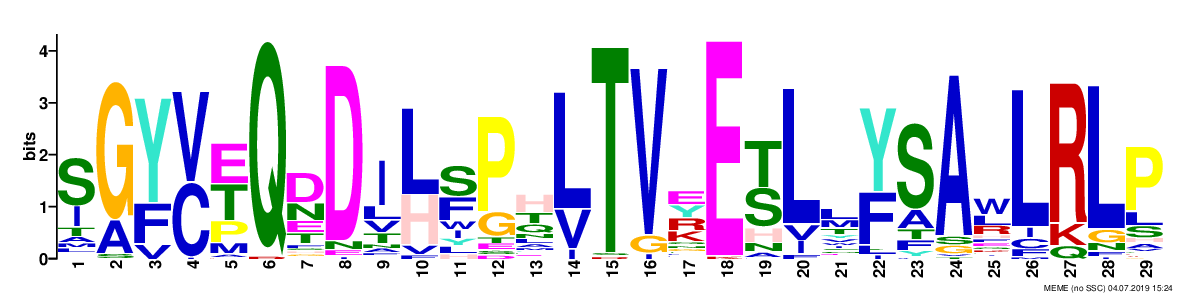 | SGYVEQBDILSPHLTVEETLLYSAWLRLP | 4.6e-612 | 63 | 29 |
| 6 | 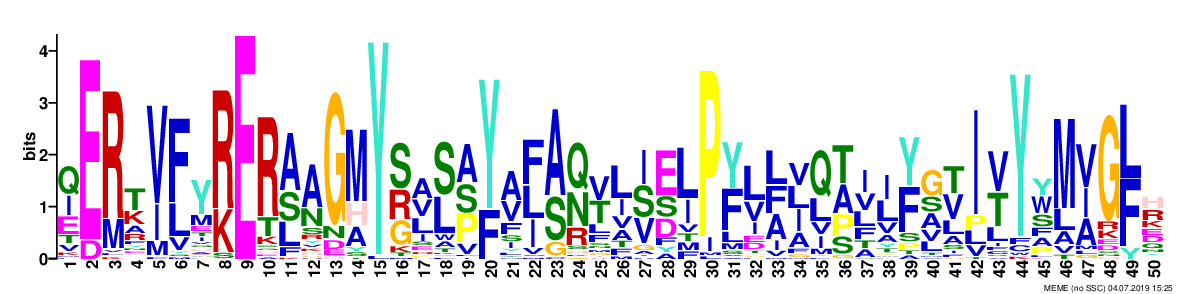 | QERTVFYRERAAGMYSASAYAFAQVJIELPYLLVQTIIYGTIVYYMVGLH | 6.7e-713 | 50 | 50 |
| 7 | 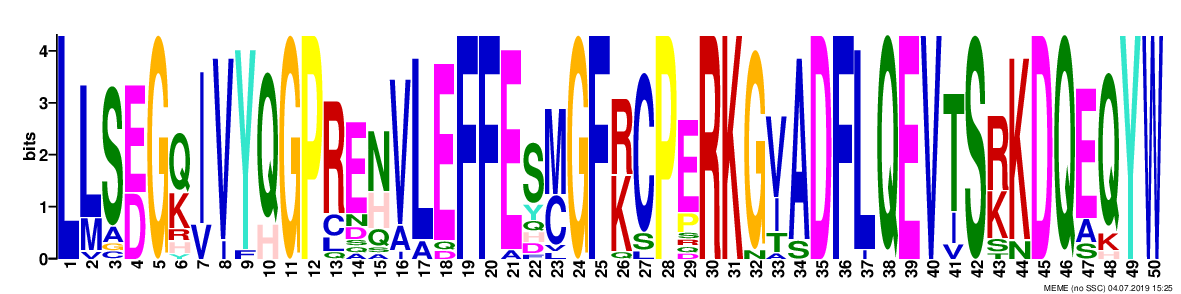 | LLSEGQIVYQGPRENVLEFFESMGFKCPERKGVADFLQEVTSRKDQEQYW | 2.7e-480 | 21 | 50 |
| 8 | 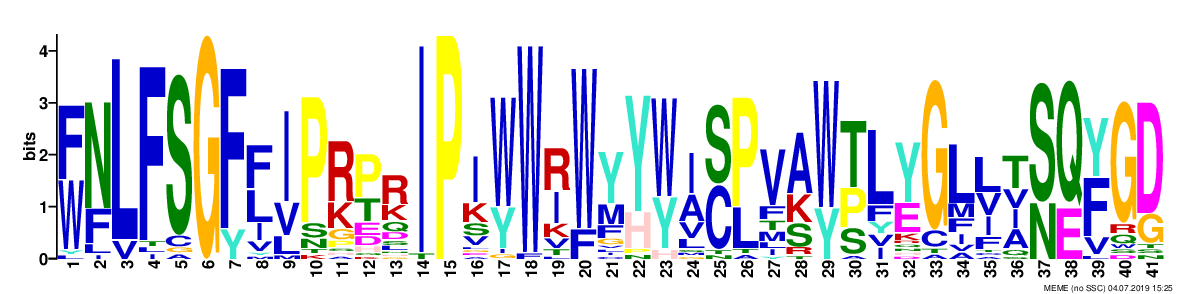 | FNLFSGFFIPRPRIPIWWRWYYWISPVAWTLYGLLTSQYGD | 1.0e-461 | 31 | 41 |
| 9 | 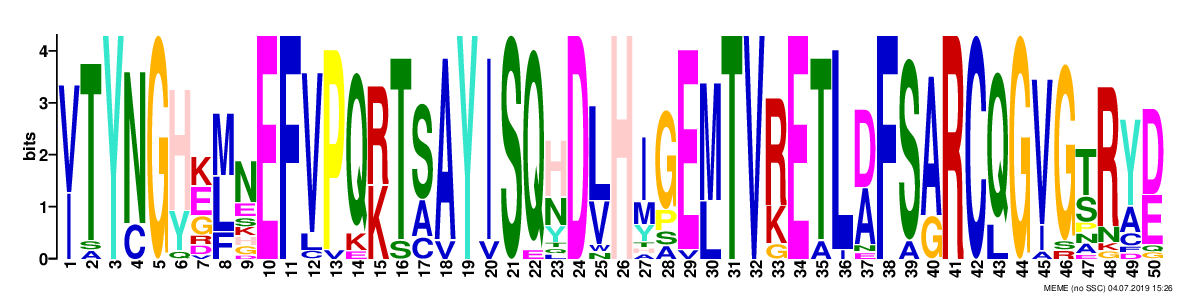 | VTYNGHEMNEFVPQRTSAYISQHDLHIGEMTVRETLDFSARCQGVGTRYD | 1.2e-434 | 21 | 50 |
| 10 | 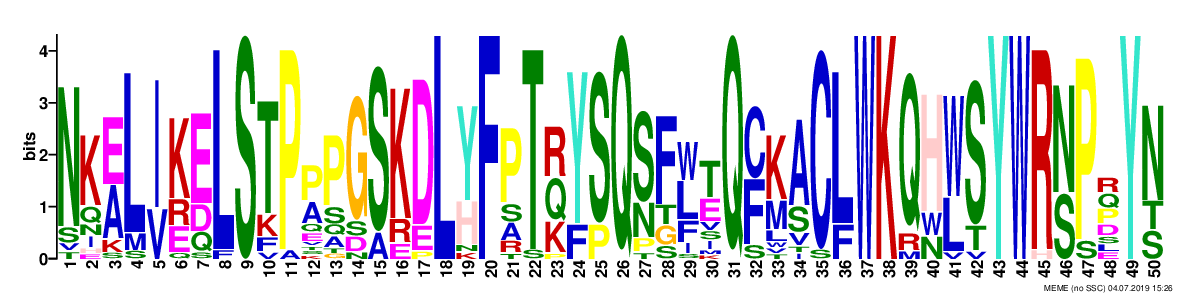 | NKELIKELSTPPPGSKDLYFPTRYSQSFLTQCKACLWKQHWSYWRNPQYN | 6.3e-409 | 21 | 50 |
| 11 | 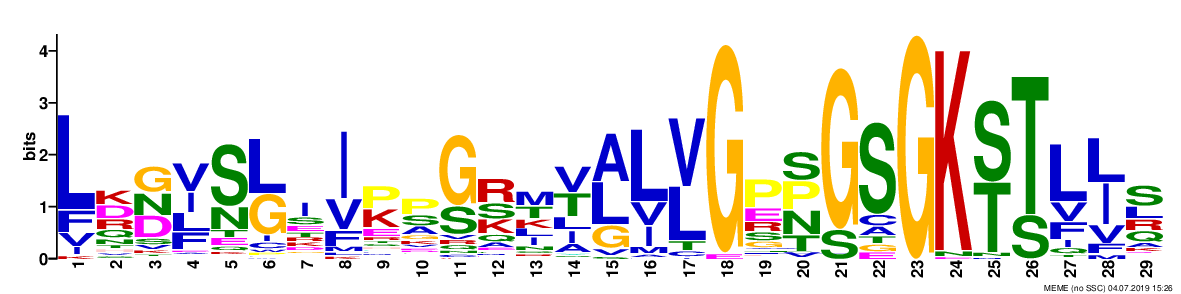 | LKGVSLIIPPGRMVALVGPSGSGKSTLJS | 3.9e-403 | 70 | 29 |
| 12 | 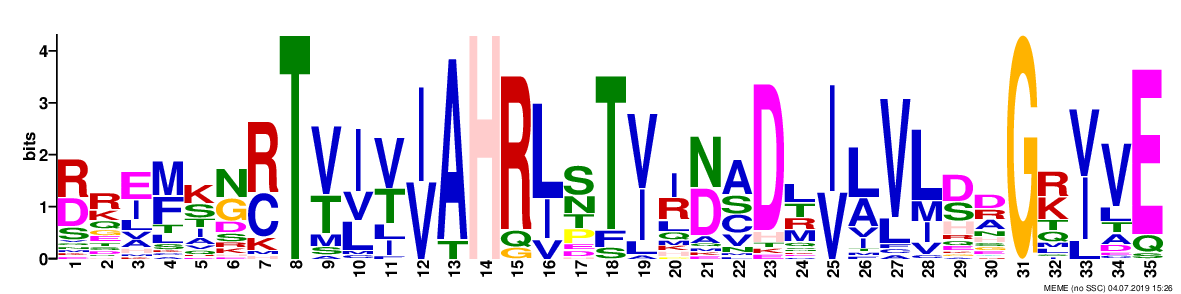 | RREMKNRTVIVIAHRLSTVIBADLILVLDDGRVVE | 1.5e-378 | 31 | 35 |
| 13 | 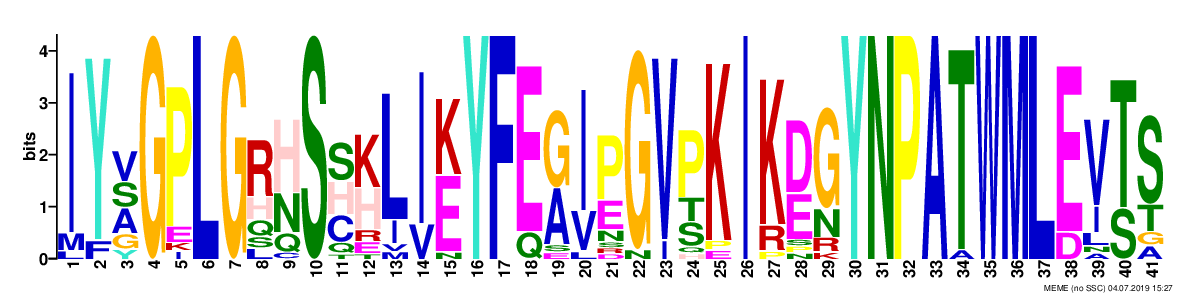 | IYVGPLGRHSSKLIEYFEGIPGVPKIKDGYNPATWMLEVTS | 2.7e-324 | 21 | 41 |
| 14 | 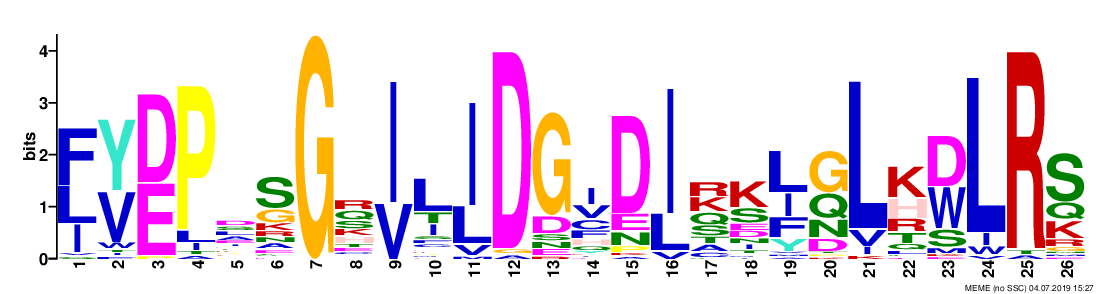 | FYDPDSGQILIDGIDIKKJGLKDLRS | 4.0e-320 | 41 | 26 |
| 15 | 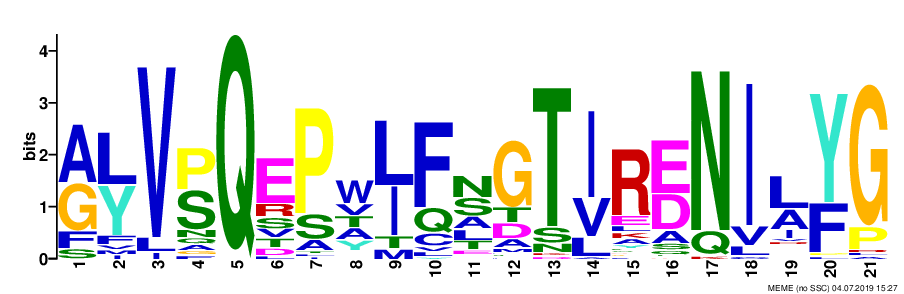 | ALVPQEPWJFNGTIRENILYG | 6.90E-286 | 53 | 21 |
| 16 | 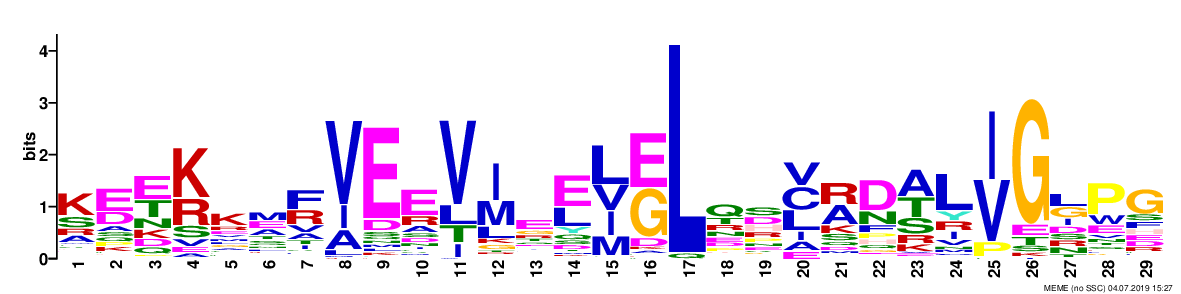 | KEEKKMFVEEVIEELELQSVRDALIGLPG | 2.30E-265 | 69 | 29 |
| 17 | 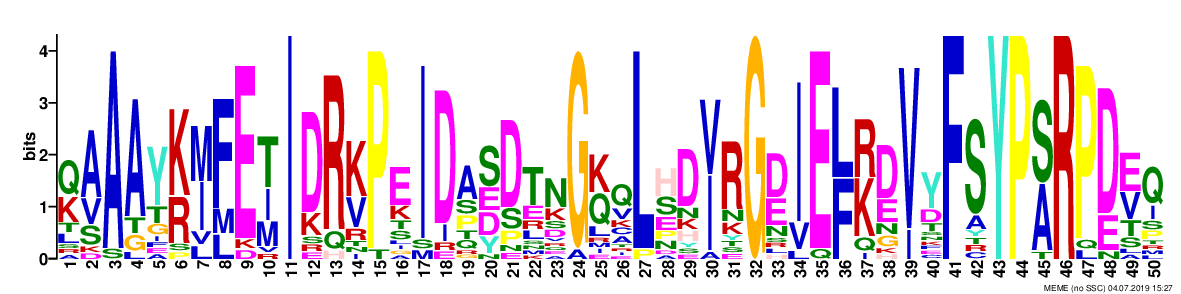 | QAAAYKMFETIDRKPEIDASDTNGKQLHDVRGDIELKDVYFSYPSRPDEQ | 8.00E-254 | 19 | 50 |
| 18 | 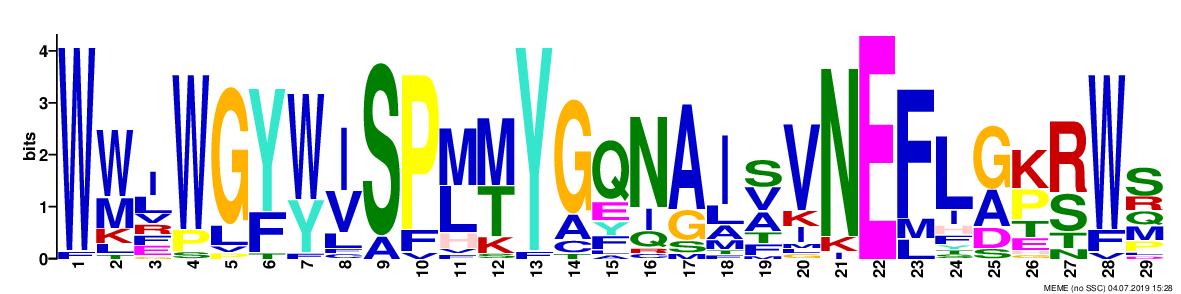 | WWIWGYWISPMMYGQNAISVNEFLGKRWS | 1.20E-240 | 25 | 29 |
| 19 | 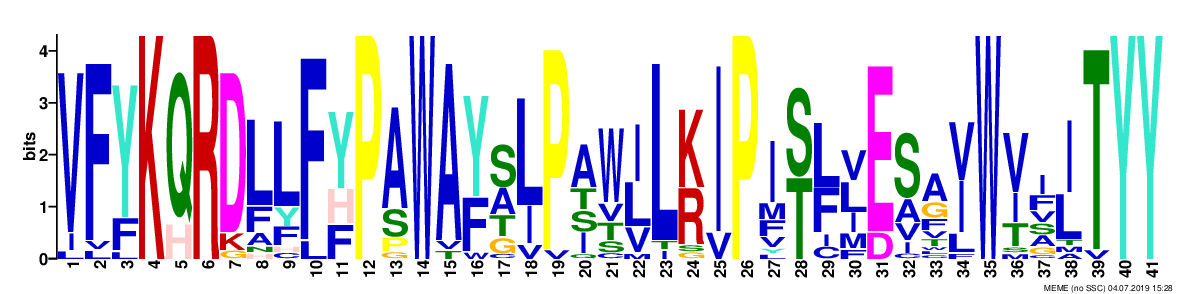 | VFYKQRDLLFYPAWAYSLPAWJLKIPISLLESAVWVIJTYY | 8.80E-278 | 21 | 41 |
| 20 | 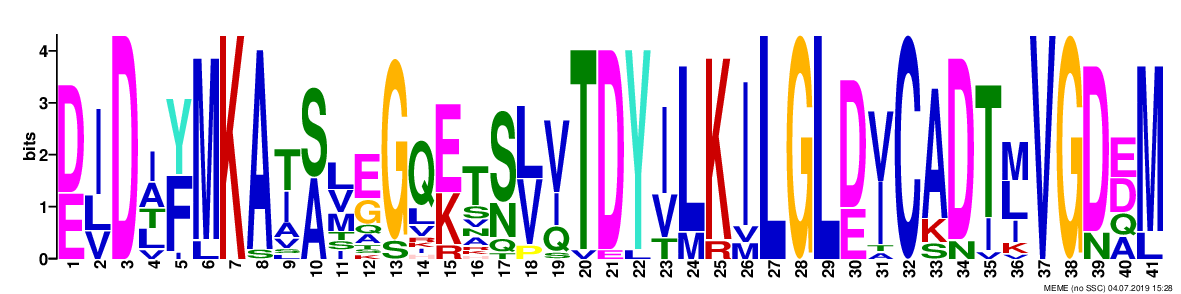 | DIDIYMKATSLEGQETSLVTDYILKILGLDVCADTLVGDEM | 1.00E-267 | 21 | 41 |

Supplemental Table 2-6 Motifs information of ABC transporter family members in *Fragaria vesca*

| Motif | LOGO | Sequence | E-value | Sites | Width |
| --- | --- | --- | --- | --- | --- |
| 1 |  | ERGRGLSGGQKQRVSIARAJLKBPSILLLDEPTSGLDAR | 1.1e-1558 | 116 | 39 |
| 2 |  | AJVGPSGSGKSTLJD | 1.7e-603 | 118 | 15 |
| 3 |  | JSGGQKQRVAIARAJLKPPKILLLDEATS | 5.0e-586 | 63 | 29 |
| 4 |  | SGYVEQDDILSPHLTVEETLVYSAWLRLP | 6.4e-542 | 51 | 29 |
| 5 |  | TVRNTADTGRTVVCTIHQPSIDIFELFDELLLL | 3.1e-535 | 47 | 33 |
| 6 |  | QPAPETYDLFDDIILLSEGQIVYQGPRENVLEFFEYMGFKCPERKGVADF | 1.4e-553 | 20 | 50 |
| 7 |  | VTYNGHELBEFVPQRTSAYISQHDLHIGEMTVRETLDFSARCQGVGTRYE | 1.5e-473 | 19 | 50 |
| 8 |  | WNLFSGFLIPRPRIPIWWRWYYWISPVAWTLYGLJTSQFGD | 6.4e-465 | 27 | 41 |
| 9 |  | LIKELSTPPPGSKDLYFPTRYSQSFLTQCKACLWKQHWSYWRNPQYNAIR | 2.7e-442 | 19 | 50 |
| 10 |  | KYMGALFFSLINIMFNGMAELAMTVFRLPVFYKQRDLLFYPAWAYALPI | 3.0e-372 | 16 | 49 |
| 11 |  | RGMVLPFQPLSLAFDHVNYYVDMPAEMKTQGIEEDRLQLLRDVSGAFRPG | 9.0e-373 | 16 | 50 |
| 12 |  | ILKDVSLIIKPGRTVALVGPSGS | 2.1e-367 | 60 | 23 |
| 13 |  | LKWLRSQIGJVPQEPTLFSGTIRENJDYG | 2.4e-410 | 46 | 29 |
| 14 |  | ERIVQEAJDREMVGRTTIVVAHRLSTVRBADMI | 1.3e-428 | 44 | 33 |
| 15 |  | KRGGQVIYAGPLGRHSHKLIEYFEAIPGVPKIKDGYNPATWMLEVTSAAV | 9.1e-413 | 19 | 50 |
| 16 |  | ERTVFYRERAAGMYSVSSYAFAQVLIEJP | 6.6e-320 | 41 | 29 |
| 17 |  | AMVGRTTVVVAHRLSTJPAADLIAVMKNGRIAEKGSHEELJ | 3.1e-376 | 38 | 41 |
| 18 |  | PFDKSKSHPAALTKKKYGISKWELFKACFSREWLLMKRNSFVYIFKTVQJ | 1.1e-375 | 19 | 50 |
| 19 |  | RGKIALVSQEPWJFSGTIRENILFGKEMA | 1.5e-341 | 40 | 29 |
| 20 |  | MIGFZWTVEKFFWFLYFMFFTFLYFTMYGMMVVAJTPNHQI | 6.60E-277 | 24 | 41 |

Supplemental Table 2-7 Motifs information of ABC transporter family members in *Rubus occidentalis*

| Motif | LOGO | Sequence | E-value | Sites | Width |
| --- | --- | --- | --- | --- | --- |
| 1 |  | LKBPSILLLDEPTSGLDS | 6.6e-669 | 89 | 18 |
| 2 |  | TFRPGELTALVGPSGSGKTTLJ | 4.7e-759 | 89 | 22 |
| 3 |  | GERGRGJSGGZKKRVAJARAJ | 3.4e-662 | 97 | 21 |
| 4 |  | GRTVVCTIHQPSIDIFEAFDELJLLKRGGQVYYG | 9.7e-578 | 39 | 34 |
| 5 |  | GYVEQBDILSPHLTVEETLLFSAWLRLPG | 4.1e-564 | 43 | 29 |
| 6 |  | STPPPGSKDLYFPTQYSQSFLTQFKACLWKQHWSYWRNPQY | 8.0e-375 | 15 | 41 |
| 7 |  | STKTVILVAHRLETLPLADDIJVLSEG | 7.1e-365 | 47 | 27 |
| 8 |  | FNLFSGFLIPRPRIPIWWRWYYWISPVAW | 2.1e-354 | 28 | 29 |
| 9 |  | ERTVFYRERAAGMYSLSSYAFAQVLIELP | 3.9e-332 | 32 | 29 |
| 10 |  | LFRFYDPAAGRILIDGIDIRSLGLKDLRSKJGJVPQEPTLF | 1.7e-439 | 28 | 41 |
| 11 |  | ILRBVSLIIKPGRMTALLGPPGSGKSTLL | 5.6e-366 | 44 | 29 |
| 12 |  | PRENVLEFFESMGFKCPERKGVADFLQEV | 1.50E-273 | 23 | 29 |
| 13 |  | RREKEAGIKPDPDIDAYMKAISVEGQKTSLVTDYILKILGLDICADTLVG | 1.6e-468 | 17 | 50 |
| 14 |  | YGISKWELFKACFSREWLLMKRNSFVYIFKTTQJTIMALITMTVFLRTQM | 1.8e-458 | 17 | 50 |
| 15 |  | RLPVFYKQRDLLFYPAWAYALPAWILKIPVSLLESAIWVIJTYYVIGFAP | 6.2e-455 | 17 | 50 |
| 16 |  | TYNGHKLNEFVPQKTSAYISQHDLHIGEMTVRETLDFSARCQGVGSRYDM | 3.5e-433 | 14 | 50 |
| 17 |  | YFEGIPGVPKIKDGYNPATWMLEVTSPAVEAQLGIDFAQIY | 1.2e-332 | 16 | 41 |
| 18 |  | EVBSETRKMFVEEVMELVELBPJRBALVGLPGVDGLSTEQRKRLTIAVEL | 8.7e-324 | 13 | 50 |
| 19 |  | EEDNEKFLKKLRDRIDRVGIDLPTVEVRFZNLSVEAECYVG | 0.00E+00 | 17 | 41 |
| 20 |  | SRQRHTSGEIINYMAVDAERIGDFIWYLHSIWMLPLQIALAJVILYKNLG | 2.00E-271 | 15 | 50 |

Supplemental Table 2-8 Motifs information of ABC transporter family members in *Prunus mume*

| Motif | LOGO | Sequence | E-value | Sites | Width |
| --- | --- | --- | --- | --- | --- |
| 1 |  | ERGRGLSGGQKQRVAIARAJLKBPSILLLDEPTSG | 2.1e-1803 | 156 | 35 |
| 2 |  | LKGVSGTFRPGELTAJVGPSGSGKSTLJD | 1.1e-1234 | 159 | 29 |
| 3 |  | DSRAAAIVMRTLRNIADTGRTVVCTIHQPSID | 5.8e-619 | 56 | 32 |
| 4 |  | VLAGRKTGGYIEGSIRISGYPKKQETFARISGYCEQNDIHSPHVTVYESL | 2.3e-635 | 35 | 50 |
| 5 |  | WNLFSGFFIPRPRIPIWWRWYYWISPVAWTLYGLLTSQFGD | 3.9e-443 | 30 | 41 |
| 6 |  | LLKPPKILLLDEATAGLDSET | 6.3e-400 | 81 | 21 |
| 7 |  | VAHRLSTVRNADDILVLSDGKIVEYGSHS | 3.1e-516 | 91 | 29 |
| 8 |  | GLKDLRSRIGJVPQEPVLFNGTIRENIDY | 6.8e-455 | 58 | 29 |
| 9 |  | TSAYVSQHDVLIPELTVRETLDFSARLQG | 3.2e-417 | 56 | 29 |
| 10 |  | NASSVQPVFALERTVFYRERAAGMYSLLPY | 5.8e-411 | 133 | 30 |
| 11 |  | RLVQEALDGVLVGRTTIVVAHRLSTJKNADVIAVVKBGVIVEKGKHEELJ | 3.0e-590 | 44 | 50 |
| 12 |  | VLEFFEYMGFKCPERKGVADFLQEVTSRKDQEQYWANKBKP | 2.3e-390 | 23 | 41 |
| 13 |  | SKDLHFPTRYSQSFWTQFKACLWKQHWSYWRNPRYNAVRFF | 1.1e-388 | 23 | 41 |
| 14 |  | RGGZEIYVGPLGRHSSKLIEYFEGIPGVSKIKDGYNPATWM | 6.1e-382 | 24 | 41 |
| 15 |  | LVTDYILKILGLDICADTLVGDEMRRGISGGQKKRVTTGEM | 1.5e-384 | 23 | 41 |
| 16 |  | ILKDVSLIIKPGRLVALVGPSGS | 6.6e-359 | 131 | 23 |
| 17 |  | RGTIAYVSQEPWJFAGTIKENILFGKEMA | 1.5e-351 | 44 | 29 |
| 18 |  | KFFWYFYFMFFTFLYFTYYGMMIVALTPNHQIAAIVASAFY | 2.60E-306 | 23 | 41 |
| 19 |  | RLPVFYKQRDLLFYPAWAYALPAWJLKIPISFVESFIWVIJTYYVIGFDP | 2.2e-385 | 22 | 50 |
| 20 |  | YGISKWELFKACFSREWLLMKRNSFVYIFKTAQJIIMAFITMTVFLRTEM | 1.7e-364 | 23 | 50 |

Supplemental Table 2-9 Motifs information of ABC transporter family members in *Rosa chinensis*

| Motif | LOGO | Sequence | E-value | Sites | Width |
| --- | --- | --- | --- | --- | --- |
| 1 |  | GERGRGLSGGQKQRVSIARAJLKBPSILLLDEPTSALDARS | 3.5e-1979 | 163 | 41 |
| 2 |  | ILKGVSGTIRPGELVALVGPSG | 1.8e-943 | 151 | 22 |
| 3 |  | GRTVVCTIHQPSIDIFELFDE | 9.5e-435 | 66 | 21 |
| 4 |  | NLSGGQKQRVALGRALLKPPKILLLDEAT | 3.3e-678 | 82 | 29 |
| 5 |  | LLSEGQIVYQGPRENVLEFFESMGFKCPERKGVADFLQEVT | 1.8e-682 | 48 | 41 |
| 6 |  | TYNGHELKEFVPQRTSAYISQHDLHI | 2.9e-511 | 179 | 26 |
| 7 |  | FRFYDPDAGKILIDGIDIKKIGLKDLRSRJGJVPQEPTLFN | 3.2e-711 | 68 | 41 |
| 8 |  | ERAAGMYSASAYAFAQVIIEJPYLLLQTIJYGAITYYMIGL | 1.1e-521 | 56 | 41 |
| 9 |  | VQEAJRREFSDCTVITVAHRJSTVRBADMILV | 6.0e-510 | 59 | 32 |
| 10 |  | ILKGVSLIIKPGRKTALVGPPGSGKSTLJ | 1.5e-472 | 85 | 29 |
| 11 |  | KTGGYIEGDITISGYPKKQETFARISGYCEQNDIHSPHVTVYESLLYSAW | 2.1e-450 | 23 | 50 |
| 12 |  | LIKELSTPPPGSKDLYFPTQYSQSFLTQCKACLWKQHLSYWRNPQYNAVR | 9.5e-446 | 26 | 50 |
| 13 |  | WNLFSGFLIPRPRIPIWWRWYYWISPVAWTLYGJJTSZFGD | 4.4e-444 | 36 | 41 |
| 14 |  | RGTIAYVSQEPWJFSGTIRENILFGKEMD | 1.1e-396 | 58 | 29 |
| 15 |  | ETLYYSAQLRLPNSMSKSEKKERAETTIREMGLQDCMBTRI | 1.40E-277 | 19 | 41 |
| 16 |  | GIMMLCGGFFRLPNDLPKPVWRYPMYYJAFHKYAFQGMYKNEFEGLTFPN | 1.80E-274 | 12 | 50 |
| 17 |  | LQEKFQSKLMEAKDKRLKATSEILRNMRILKLQAWETKFLK | 1.00E-295 | 30 | 41 |
| 18 |  | TVRETLDFSARCQGVGTRYEMLAELSRRE | 3.00E-273 | 26 | 29 |
| 19 |  | LLMKRGGQVIYAGPLGRHSSKLIEYFEGIPGVPKIKDGYNPATWMLEVTS | 1.2e-389 | 25 | 50 |
| 20 |  | IYMGALFFSLIIVMFNGFAELSMTVSRLPVFYKQRDLLFYPAWAYSLPAW | 5.7e-378 | 24 | 50 |
