# Supplementary material for: Genome-Wide Identification and Characterization of ABC Transporters in Nine Rosaceae Species Identifying MdABCG28 as a Possible Cytokinin Transporter linked to Dwarfing
Source: Int J Mol Sci. 2019 Nov 17;20(22):5783. doi: 10.3390/ijms20225783 (PMC6887749; doi:10.3390/ijms20225783)
Supplement: Supplementary file 1 [file ijms-20-05783-s001.zip › Supplemental Table 3.docx]

Supplemental Table 3. Primers used for the construction of recombinant plasmids of promoter activity assay and plant transformation of *MdABCG28*

|  | Forward primer | Reverse primer |
| --- | --- | --- |
| Primers for *MdABCG28* promoter cloning | (EcoRI)CCGGAATTCGTGCGTGCGAGAGGAGATAA | (KpnI)CGGGGTACCGAACGAGCCTGGGAGCACAT |
| Overlap PCR primers for A1 construct | TTTCCAAAAACACAAAAAGAAAATTTAAATGAACCGTCTTGACATGTTGATAAGCCTAAAAAAAAGATGCTGGAATG | CATTCCAGCATCTTTTTTTTAGGCTTATCAACATGTCAAGACGGTTCATTTAAATTTTCTTTTTGTGTTTTTGGAAA |
| Overlap PCR primers for A2 construct | TTTCCAAAAACACAAAAAGAAAATTTAAATGAACCGTCTTCACATGTTGATAAGCCTAAAAAAAAGATGCTGGAATG | CATTCCAGCATCTTTTTTTTAGGCTTATCAACATGTGAAGACGGTTCATTTAAATTTTCTTTTTGTGTTTTTGGAAA |
| Primers for *MdABCG28* overexpressing construct | (EcoRI)CCGGAATTCATGTGCTCCCAGGCTCGTTC | (KpnI)CGGGGTACCTTATCTCCTCAAGCGCACTCGATGG |
