# Supplementary material for: Genome-Wide Identification and Characterization of ABC Transporters in Nine Rosaceae Species Identifying MdABCG28 as a Possible Cytokinin Transporter linked to Dwarfing
Source: Int J Mol Sci. 2019 Nov 17;20(22):5783. doi: 10.3390/ijms20225783 (PMC6887749; doi:10.3390/ijms20225783)
Supplement: Supplementary file 1 [file ijms-20-05783-s001.zip › Supplemental Table 4.docx]

Supplemental Table 4. Primers for quantitative RT-PCR of 10 potential phytohormone transporters in MdABCG subfamily genes

| Gene | Forward primer | Reverse primer |
| --- | --- | --- |
| MdABCG6 | TGCAGAACGAGTTCCAGGAC | GTCGTCAGACACGTGGAAGT |
| MdABCG20 | CGATTTTTCAGCCCGCTGTC | GTCCGTCTGAAGGGTTCGTT |
| MdABCG22 | AGGTTAGTGGCTTACCTTGCAT | CCATTGAACGATTACAGTGTTGC |
| MdABCG27 | TCTGGTTATTTGGCGGGAGC | GTTTGCGGTTATGCCCTTGG |
| MdABCG28 | TCGAGGAGGAACGTAGCTGA | GGGTGTGCTCGCAACTAGAA |
| MdABCG29 | GGAACCTTGGATCAAAAAGTAAGC | GCGTATGGCAAAGCGGAATA |
| MdABCG41 | GCTCATGATGATGCAGTTTGTGG | ATGTTTGTGGGTGAGCCTGT |
| MdABCG56 | CTGTGCTCATTGTTTTGGTGAAAG | TTCCTCGACAGTGTTGATCCG |
| MdABCG67 | GGGGTACTTTGGTAACTCCCAT | CACCAATTGTTCGGACTTGGTC |
| MdABCG70 | CTCTCCCAACCATCGCTAAC | CTTGTTCTGGGTTCCAGCAG |
