# Supplementary material for: Genome-Wide Identification and Characterization of ABC Transporters in Nine Rosaceae Species Identifying MdABCG28 as a Possible Cytokinin Transporter linked to Dwarfing
Source: Int J Mol Sci. 2019 Nov 17;20(22):5783. doi: 10.3390/ijms20225783 (PMC6887749; doi:10.3390/ijms20225783)
Supplement: Supplementary file 1 [file Supplemental Figure 1.pdf › Supplemental Figure 1.pdf]

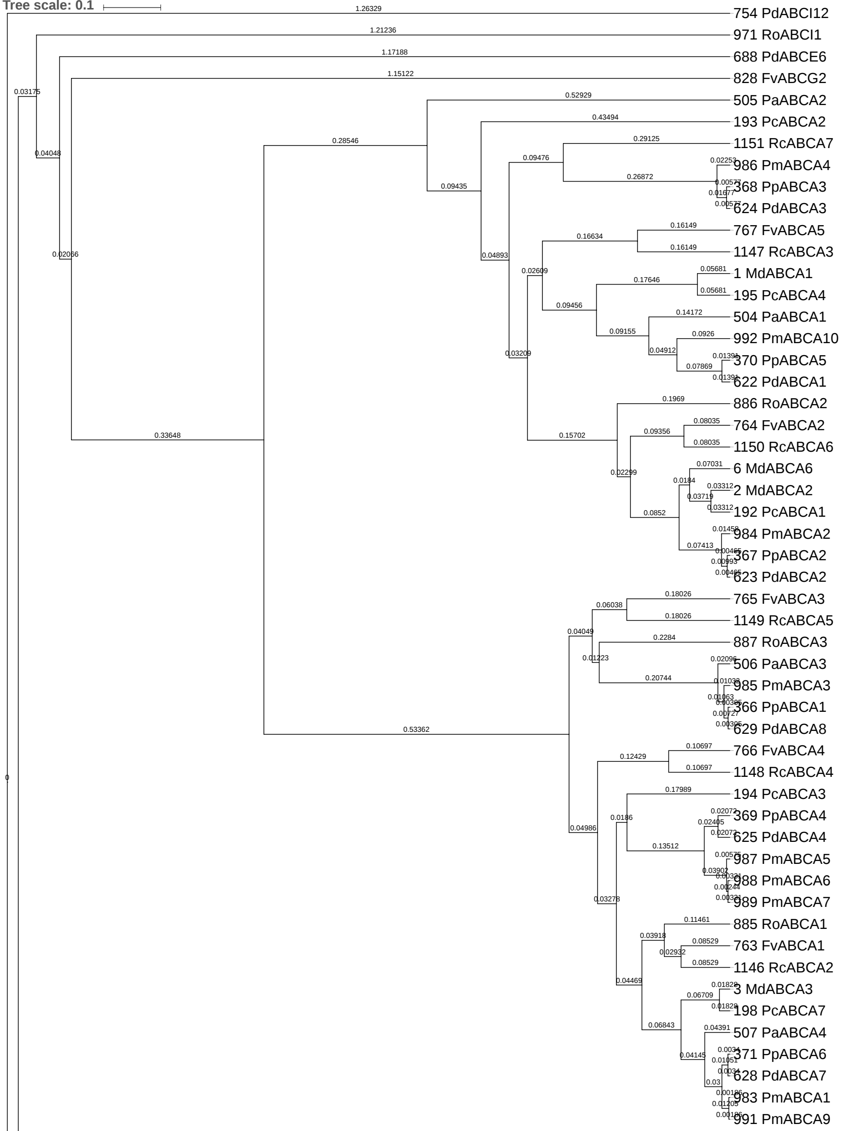

Tree scale: 0.1
754 PdABCI12
971 RoABCI1
688 PdABCE6
828 FvABCG2
505 PaABCA2
193 PcABCA2
1151 RcABCA7
986 PmABCA4
368 PpABCA3
624 PdABCA3
767 FvABCA5
1147 RcABCA3
1 MdABCA1
195 PcABCA4
504 PaABCA1
992 PmABCA10
370 PpABCA5
622 PdABCA1
886 RoABCA2
764 FvABCA2
1150 RcABCA6
6 MdABCA6
2 MdABCA2
192 PcABCA1
984 PmABCA2
367 PpABCA2
623 PdABCA2
765 FvABCA3
1149 RcABCA5
887 RoABCA3
506 PaABCA3
985 PmABCA3
366 PpABCA1
629 PdABCA8
766 FvABCA4
1148 RcABCA4
194 PcABCA3
369 PpABCA4
625 PdABCA4
987 PmABCA5
988 PmABCA6
989 PmABCA7
885 RoABCA1
763 FvABCA1
1146 RcABCA2
3 MdABCA3
198 PcABCA7
507 PaABCA4
371 PpABCA6
628 PdABCA7
983 PmABCA1
991 PmABCA9

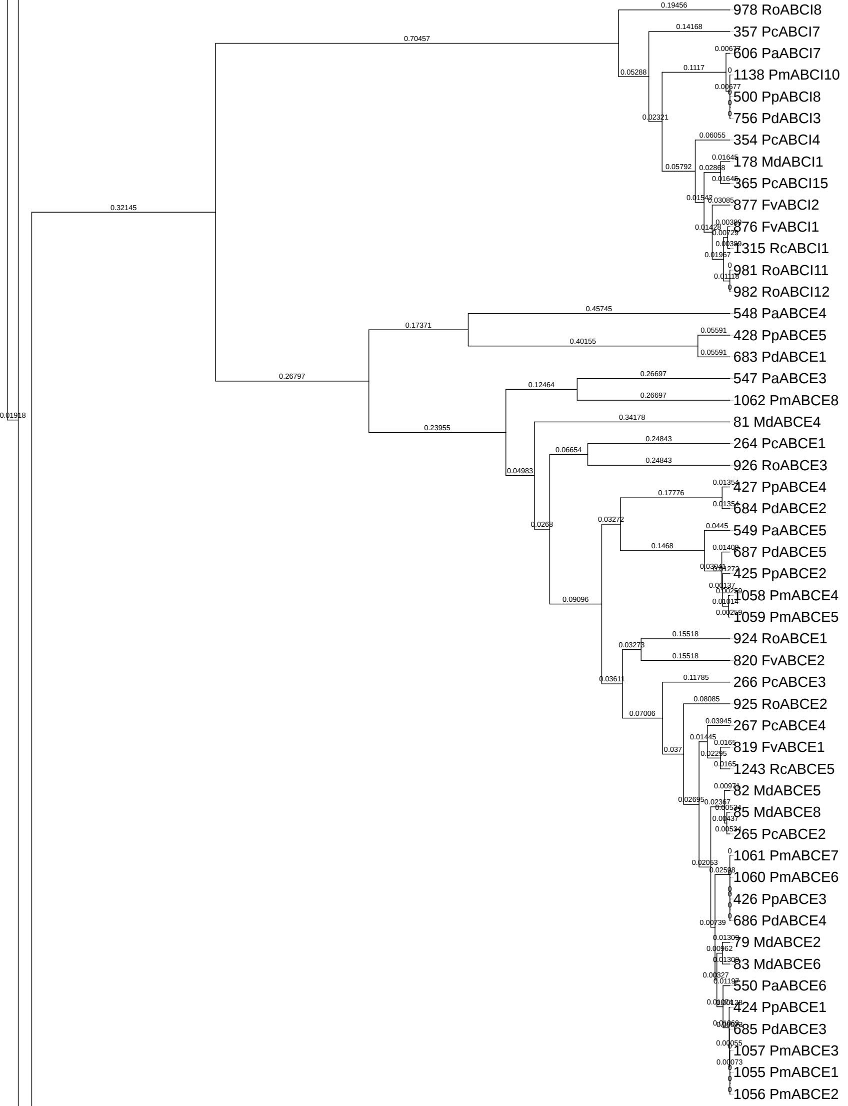

0.19456
978 RoABCI8
0.70457
0.14168
357 PcABCI7
606 PaABCI7
0.1117
0.05288
1138 PmABCI10
500 PpABCI8
756 PdABCI3
0.02321
0.06055
354 PcABCI4
178 MdABCI1
0.05792
365 PcABCI15
0.32145
877 FvABCI2
876 FvABCI1
1315 RcABCI1
981 RoABCI11
982 RoABCI12
0.45745
548 PaABCE4
0.17371
0.05591
428 PpABCE5
0.40155
0.05591
683 PdABCE1
0.26797
0.26697
547 PaABCE3
0.12464
0.26697
1062 PmABCE8
0.34178
81 MdABCE4
0.23955
0.06654
0.24843
264 PcABCE1
0.24843
926 RoABCE3
0.04983
427 PpABCE4
0.17776
684 PdABCE2
0.03272
0.0445
549 PaABCE5
0.1468
687 PdABCE5
425 PpABCE2
1058 PmABCE4
0.09096
1059 PmABCE5
0.15518
924 RoABCE1
0.03273
0.15518
820 FvABCE2
0.11785
266 PcABCE3
0.08085
925 RoABCE2
0.07006
0.03945
267 PcABCE4
819 FvABCE1
0.037
1243 RcABCE5
82 MdABCE5
0.02695
85 MdABCE8
265 PcABCE2
1061 PmABCE7
1060 PmABCE6
426 PpABCE3
686 PdABCE4
79 MdABCE2
83 MdABCE6
550 PaABCE6
424 PpABCE1
685 PdABCE3
1057 PmABCE3
1055 PmABCE1
1056 PmABCE2
0.01918

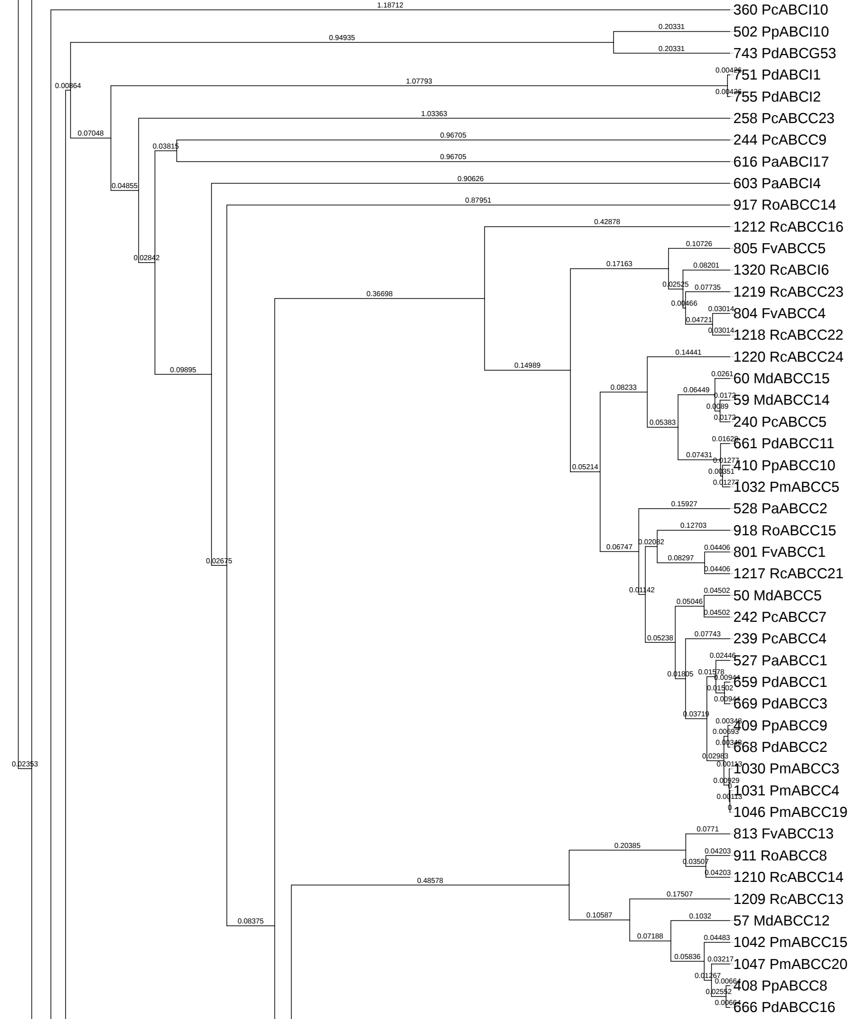
360 PcABCI10
502 PpABCI10
743 PdABCG53
751 PdABCI1
755 PdABCI2
258 PcABCC23
244 PcABCC9
616 PaABCI17
603 PaABCI4
917 RoABCC14
1212 RcABCC16
805 FvABCC5
1320 RcABCI6
1219 RcABCC23
804 FvABCC4
1218 RcABCC22
1220 RcABCC24
60 MdABCC15
59 MdABCC14
240 PcABCC5
661 PdABCC11
410 PpABCC10
1032 PmABCC5
528 PaABCC2
918 RoABCC15
801 FvABCC1
1217 RcABCC21
50 MdABCC5
242 PcABCC7
239 PcABCC4
527 PaABCC1
659 PdABCC1
669 PdABCC3
409 PpABCC9
668 PdABCC2
1030 PmABCC3
1031 PmABCC4
1046 PmABCC19
813 FvABCC13
911 RoABCC8
1210 RcABCC14
1209 RcABCC13
57 MdABCC12
1042 PmABCC15
1047 PmABCC20
408 PpABCC8
666 PdABCC16

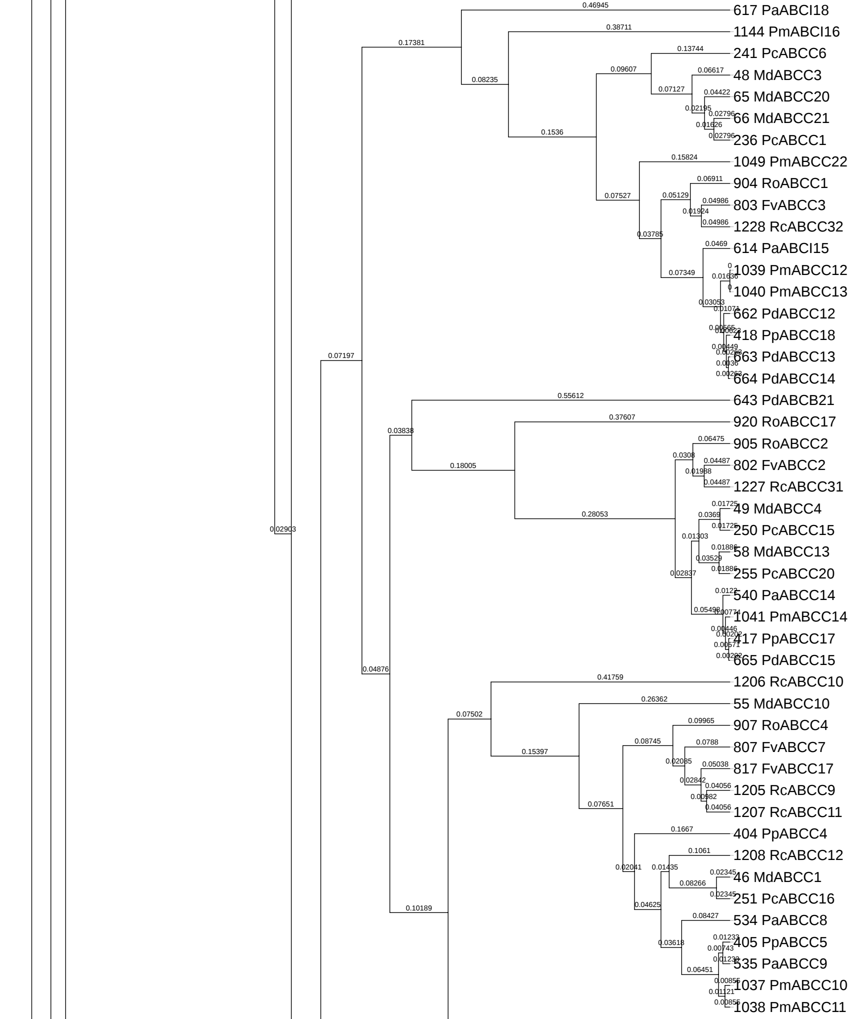

617 PaABCI18
1144 PmABCI16
241 PcABCC6
48 MdABCC3
65 MdABCC20
66 MdABCC21
236 PcABCC1
1049 PmABCC22
904 RoABCC1
803 FvABCC3
1228 RcABCC32
614 PaABCI15
1039 PmABCC12
1040 PmABCC13
662 PdABCC12
418 PpABCC18
663 PdABCC13
664 PdABCC14
643 PdABCB21
920 RoABCC17
905 RoABCC2
802 FvABCC2
1227 RcABCC31
49 MdABCC4
250 PcABCC15
58 MdABCC13
255 PcABCC20
540 PaABCC14
1041 PmABCC14
417 PpABCC17
665 PdABCC15
1206 RcABCC10
55 MdABCC10
907 RoABCC4
807 FvABCC7
817 FvABCC17
1205 RcABCC9
1207 RcABCC11
404 PpABCC4
1208 RcABCC12
46 MdABCC1
251 PcABCC16
534 PaABCC8
405 PpABCC5
535 PaABCC9
1037 PmABCC10
1038 PmABCC11

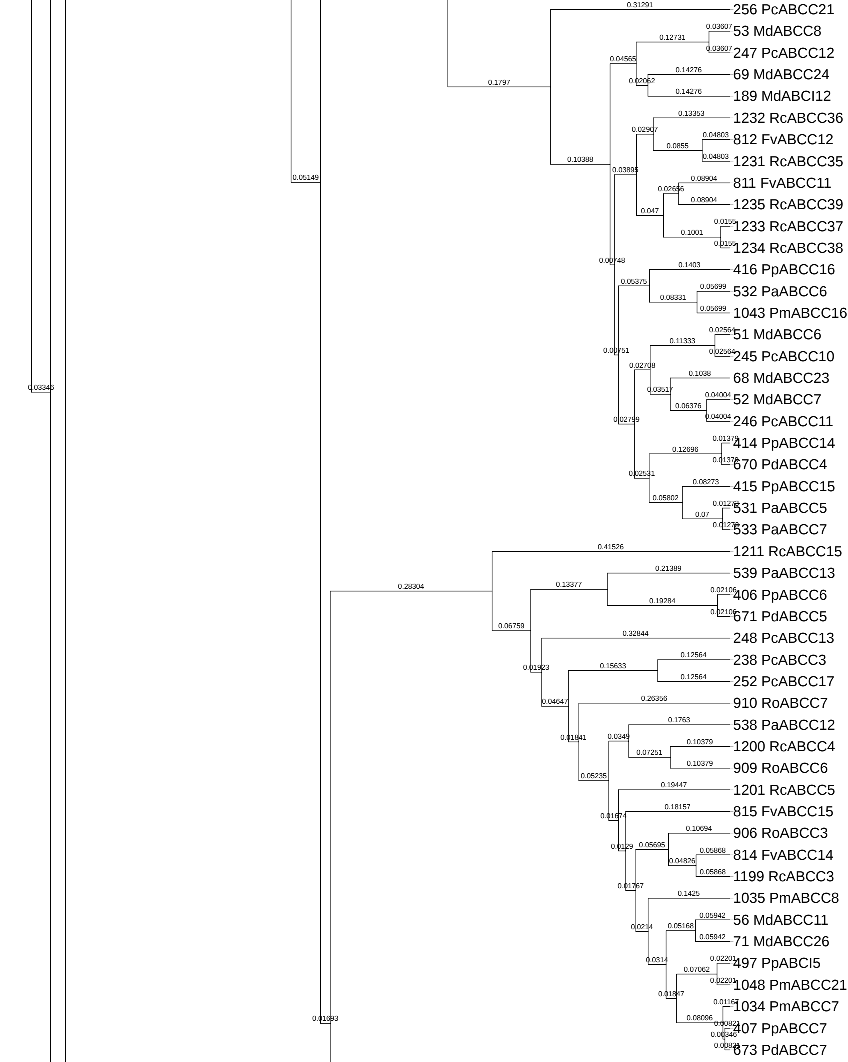
256 PcABCC21
53 MdABCC8
247 PcABCC12
69 MdABCC24
189 MdABCI12
1232 RcABCC36
812 FvABCC12
1231 RcABCC35
811 FvABCC11
1235 RcABCC39
1233 RcABCC37
1234 RcABCC38
416 PpABCC16
532 PaABCC6
1043 PmABCC16
51 MdABCC6
245 PcABCC10
68 MdABCC23
52 MdABCC7
246 PcABCC11
414 PpABCC14
670 PdABCC4
415 PpABCC15
531 PaABCC5
533 PaABCC7
1211 RcABCC15
539 PaABCC13
406 PpABCC6
671 PdABCC5
248 PcABCC13
238 PcABCC3
252 PcABCC17
910 RoABCC7
538 PaABCC12
1200 RcABCC4
909 RoABCC6
1201 RcABCC5
815 FvABCC15
906 RoABCC3
814 FvABCC14
1199 RcABCC3
1035 PmABCC8
56 MdABCC11
71 MdABCC26
497 PpABCI5
1048 PmABCC21
1034 PmABCC7
407 PpABCC7
673 PdABCC7

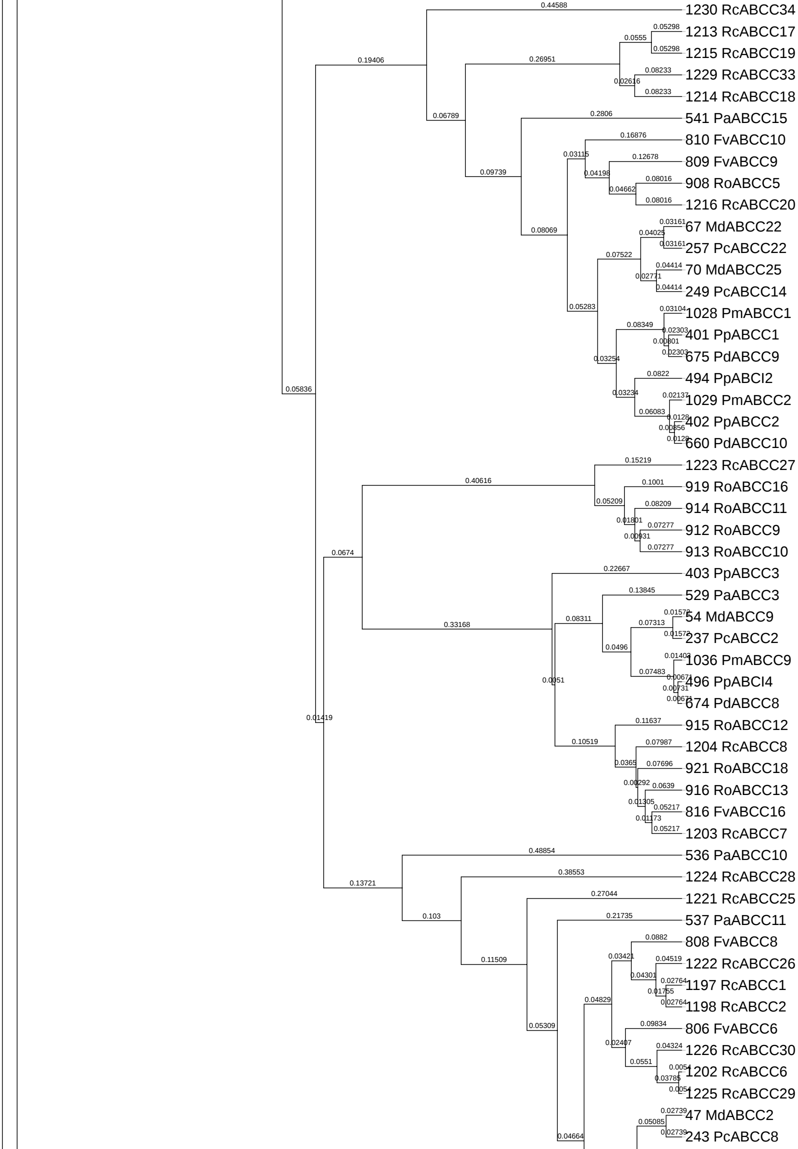
1230 RcABCC34
1213 RcABCC17
1215 RcABCC19
1229 RcABCC33
1214 RcABCC18
541 PaABCC15
810 FvABCC10
809 FvABCC9
908 RoABCC5
1216 RcABCC20
67 MdABCC22
257 PcABCC22
70 MdABCC25
249 PcABCC14
1028 PmABCC1
401 PpABCC1
675 PdABCC9
494 PpABCI2
1029 PmABCC2
402 PpABCC2
660 PdABCC10
1223 RcABCC27
919 RoABCC16
914 RoABCC11
912 RoABCC9
913 RoABCC10
403 PpABCC3
529 PaABCC3
54 MdABCC9
237 PcABCC2
1036 PmABCC9
496 PpABCI4
674 PdABCC8
915 RoABCC12
1204 RcABCC8
921 RoABCC18
916 RoABCC13
816 FvABCC16
1203 RcABCC7
536 PaABCC10
1224 RcABCC28
1221 RcABCC25
537 PaABCC11
808 FvABCC8
1222 RcABCC26
1197 RcABCC1
1198 RcABCC2
806 FvABCC6
1226 RcABCC30
1202 RcABCC6
1225 RcABCC29
47 MdABCC2
243 PcABCC8

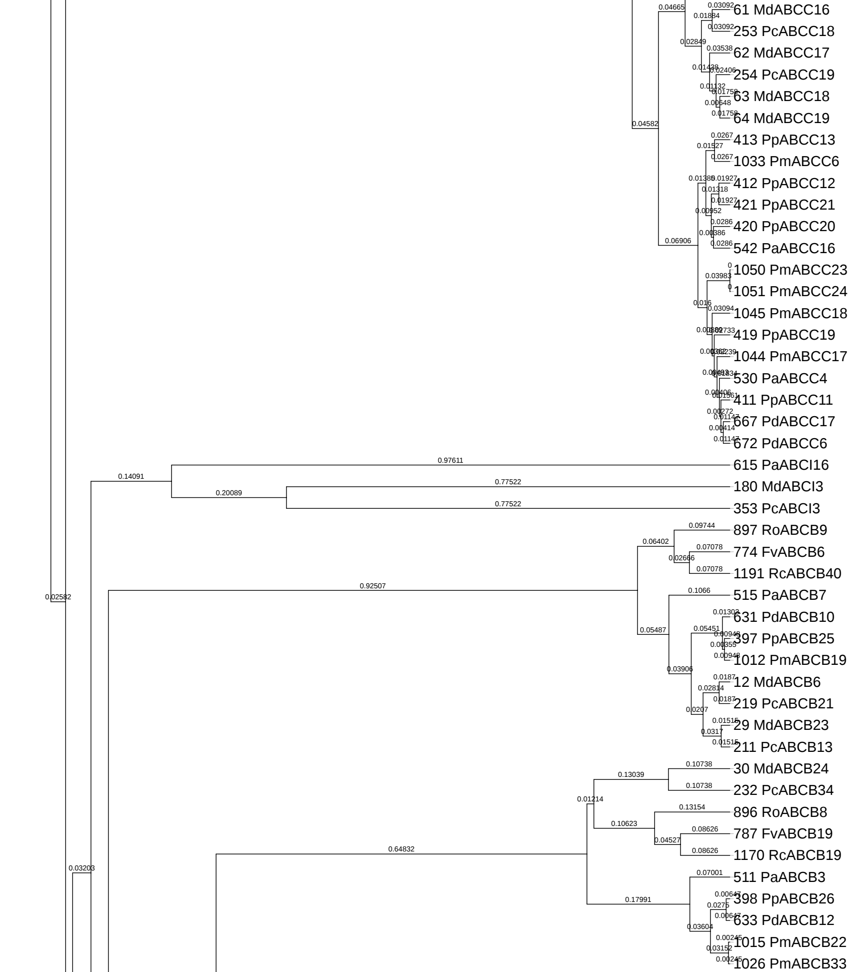
61 MdABCC16
253 PcABCC18
62 MdABCC17
254 PcABCC19
63 MdABCC18
64 MdABCC19
413 PpABCC13
1033 PmABCC6
412 PpABCC12
421 PpABCC21
420 PpABCC20
542 PaABCC16
1050 PmABCC23
1051 PmABCC24
1045 PmABCC18
419 PpABCC19
1044 PmABCC17
530 PaABCC4
411 PpABCC11
667 PdABCC17
672 PdABCC6
615 PaABCI16
180 MdABCI3
353 PcABCI3
897 RoABCB9
774 FvABCB6
1191 RcABCB40
515 PaABCB7
631 PdABCB10
397 PpABCB25
1012 PmABCB19
12 MdABCB6
219 PcABCB21
29 MdABCB23
211 PcABCB13
30 MdABCB24
232 PcABCB34
896 RoABCB8
787 FvABCB19
1170 RcABCB19
511 PaABCB3
398 PpABCB26
633 PdABCB12
1015 PmABCB22
1026 PmABCB33

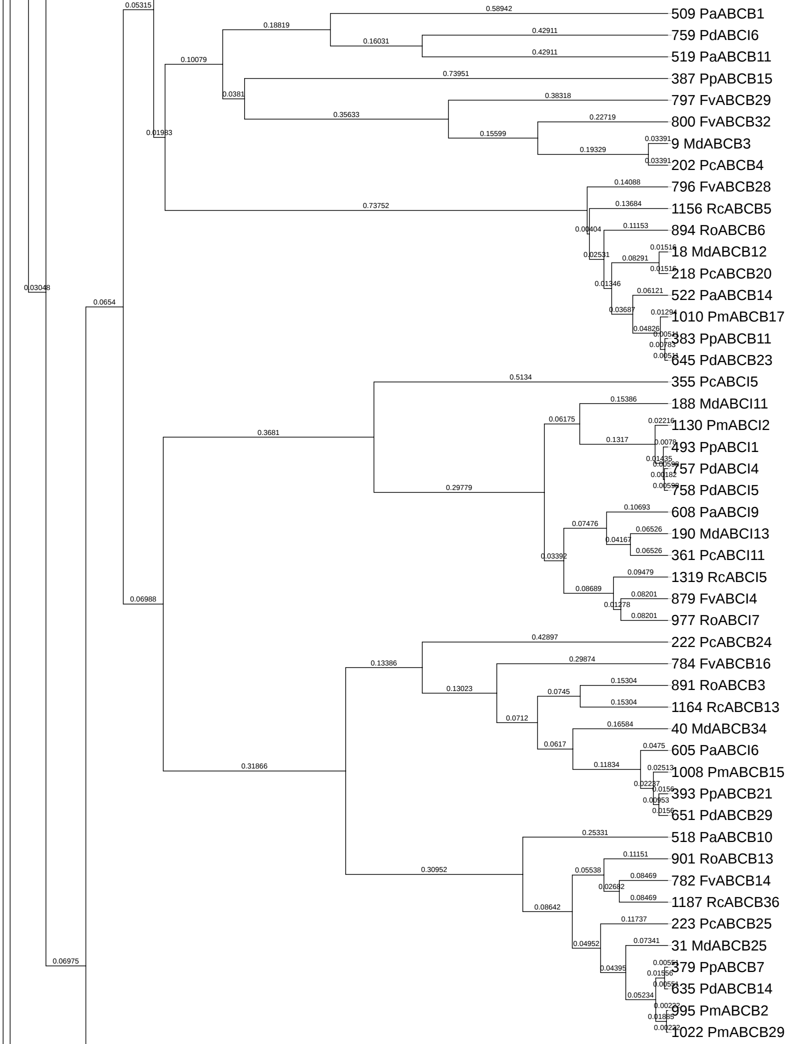
509 PaABCB1
759 PdABCI6
519 PaABCB11
387 PpABCB15
797 FvABCB29
800 FvABCB32
9 MdABCB3
202 PcABCB4
796 FvABCB28
1156 RcABCB5
894 RoABCB6
18 MdABCB12
218 PcABCB20
522 PaABCB14
1010 PmABCB17
383 PpABCB11
645 PdABCB23
355 PcABCI5
188 MdABCI11
1130 PmABCI2
493 PpABCI1
757 PdABCI4
758 PdABCI5
608 PaABCI9
190 MdABCI13
361 PcABCI11
1319 RcABCI5
879 FvABCI4
977 RoABCI7
222 PcABCB24
784 FvABCB16
891 RoABCB3
1164 RcABCB13
40 MdABCB34
605 PaABCI6
1008 PmABCB15
393 PpABCB21
651 PdABCB29
518 PaABCB10
901 RoABCB13
782 FvABCB14
1187 RcABCB36
223 PcABCB25
31 MdABCB25
379 PpABCB7
635 PdABCB14
995 PmABCB2
1022 PmABCB29

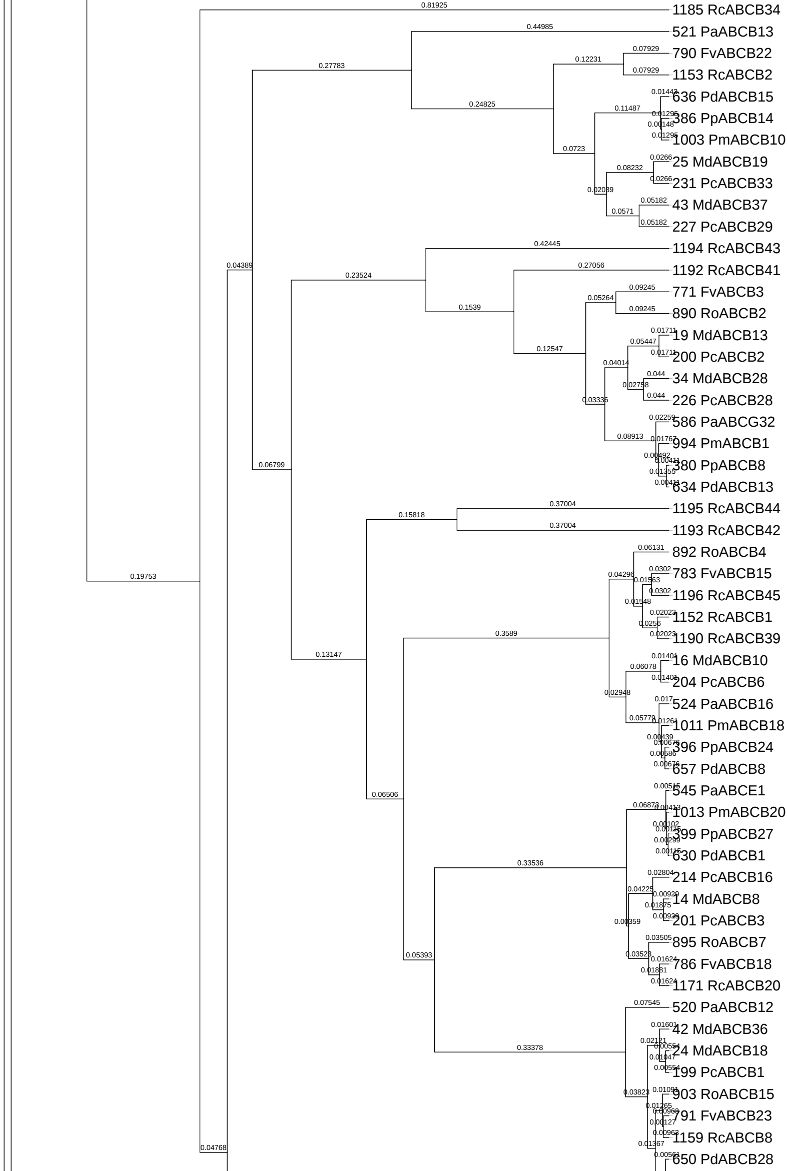
1185 RcABCB34
521 PaABCB13
790 FvABCB22
1153 RcABCB2
636 PdABCB15
386 PpABCB14
1003 PmABCB10
25 MdABCB19
231 PcABCB33
43 MdABCB37
227 PcABCB29
1194 RcABCB43
1192 RcABCB41
771 FvABCB3
890 RoABCB2
19 MdABCB13
200 PcABCB2
34 MdABCB28
226 PcABCB28
586 PaABCG32
994 PmABCB1
380 PpABCB8
634 PdABCB13
1195 RcABCB44
1193 RcABCB42
892 RoABCB4
783 FvABCB15
1196 RcABCB45
1152 RcABCB1
1190 RcABCB39
16 MdABCB10
204 PcABCB6
524 PaABCB16
1011 PmABCB18
396 PpABCB24
657 PdABCB8
545 PaABCE1
1013 PmABCB20
399 PpABCB27
630 PdABCB1
214 PcABCB16
14 MdABCB8
201 PcABCB3
895 RoABCB7
786 FvABCB18
1171 RcABCB20
520 PaABCB12
42 MdABCB36
24 MdABCB18
199 PcABCB1
903 RoABCB15
791 FvABCB23
1159 RcABCB8
650 PdABCB28

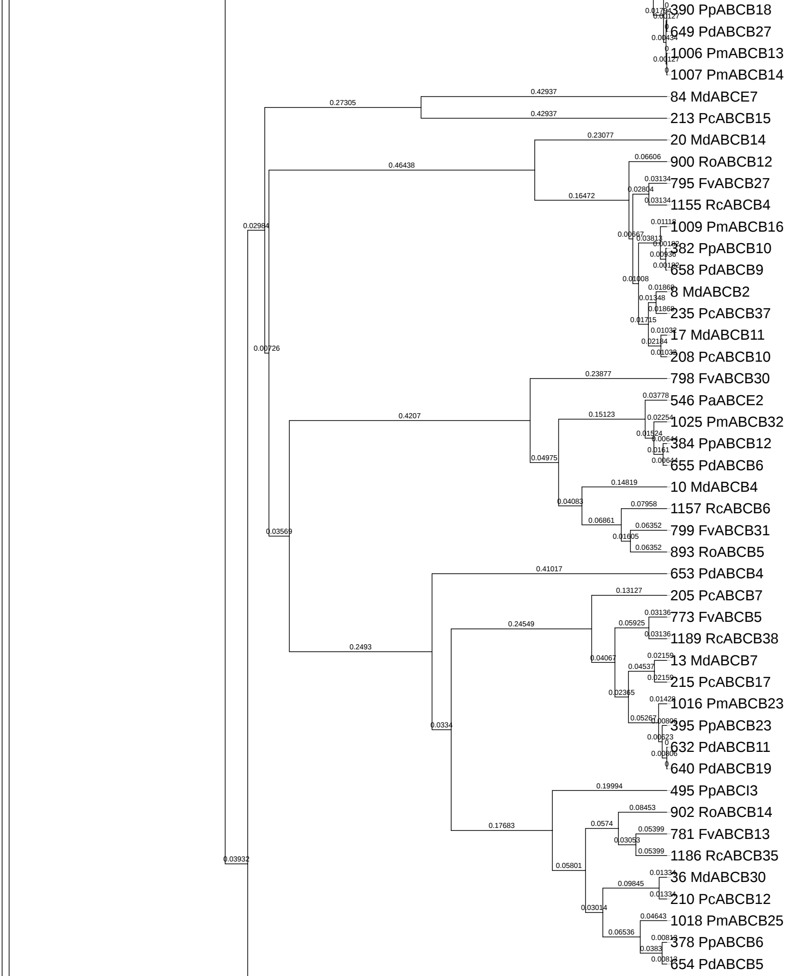
390 PpABCB18
649 PdABCB27
1006 PmABCB13
1007 PmABCB14
84 MdABCE7
213 PcABCB15
20 MdABCB14
900 RoABCB12
795 FvABCB27
1155 RcABCB4
1009 PmABCB16
382 PpABCB10
658 PdABCB9
8 MdABCB2
235 PcABCB37
17 MdABCB11
208 PcABCB10
798 FvABCB30
546 PaABCE2
1025 PmABCB32
384 PpABCB12
655 PdABCB6
10 MdABCB4
1157 RcABCB6
799 FvABCB31
893 RoABCB5
653 PdABCB4
205 PcABCB7
773 FvABCB5
1189 RcABCB38
13 MdABCB7
215 PcABCB17
1016 PmABCB23
395 PpABCB23
632 PdABCB11
640 PdABCB19
495 PpABCI3
902 RoABCB14
781 FvABCB13
1186 RcABCB35
36 MdABCB30
210 PcABCB12
1018 PmABCB25
378 PpABCB6
654 PdABCB5
0.42937
0.27305
0.42937
0.23077
0.46438
0.06606
0.03134
0.02804
0.16472
0.03134
0.02984
0.00667
0.01008
0.01715
0.00726
0.23877
0.03778
0.4207
0.15123
0.02254
0.01524
0.04975
0.14819
0.04083
0.07958
0.06861
0.06352
0.03569
0.01605
0.06352
0.41017
0.13127
0.03136
0.24549
0.05925
0.03136
0.2493
0.04067
0.02159
0.04537
0.02159
0.02365
0.05267
0.0334
0.19994
0.08453
0.0574
0.05399
0.03053
0.17683
0.05399
0.05801
0.03932
0.01334
0.09845
0.01334
0.03014
0.04643
0.06536
0.0383

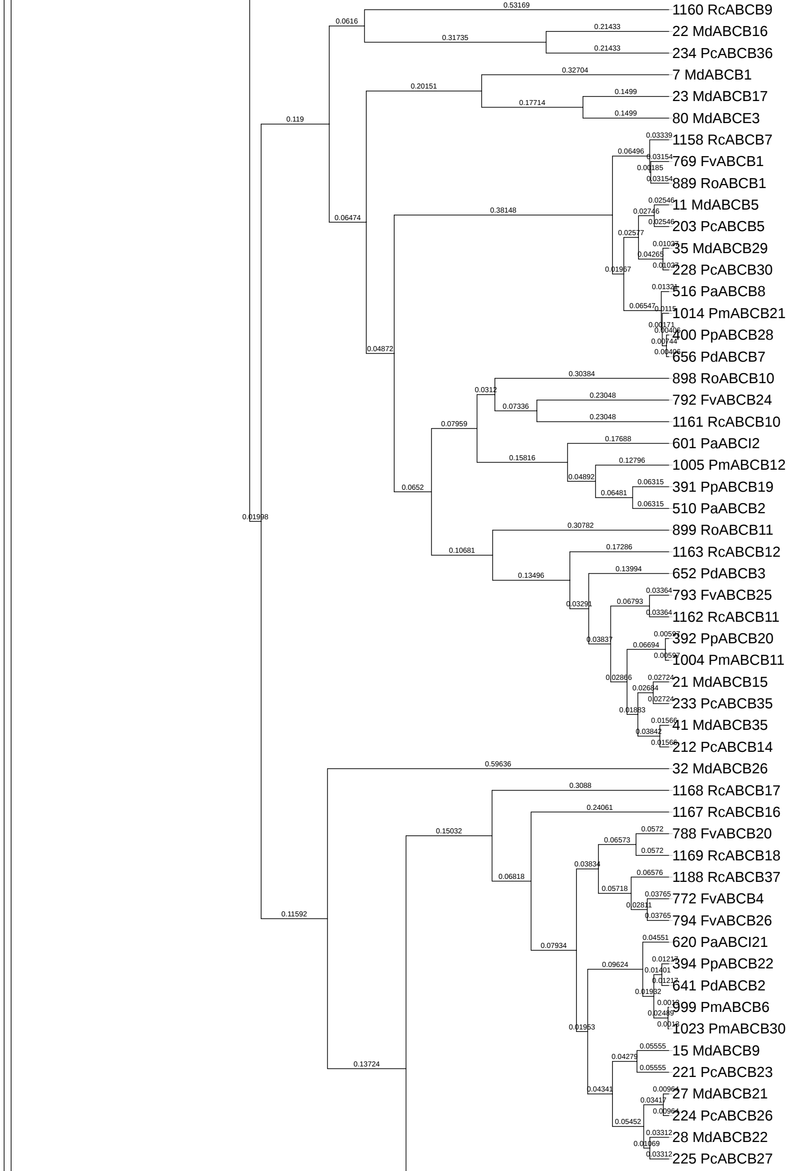
1160 RcABCB9
22 MdABCB16
234 PcABCB36
7 MdABCB1
23 MdABCB17
80 MdABCE3
1158 RcABCB7
769 FvABCB1
889 RoABCB1
11 MdABCB5
203 PcABCB5
35 MdABCB29
228 PcABCB30
516 PaABCB8
1014 PmABCB21
400 PpABCB28
656 PdABCB7
898 RoABCB10
792 FvABCB24
1161 RcABCB10
601 PaABCI2
1005 PmABCB12
391 PpABCB19
510 PaABCB2
899 RoABCB11
1163 RcABCB12
652 PdABCB3
793 FvABCB25
1162 RcABCB11
392 PpABCB20
1004 PmABCB11
21 MdABCB15
233 PcABCB35
41 MdABCB35
212 PcABCB14
32 MdABCB26
1168 RcABCB17
1167 RcABCB16
788 FvABCB20
1169 RcABCB18
1188 RcABCB37
772 FvABCB4
794 FvABCB26
620 PaABCI21
394 PpABCB22
641 PdABCB2
999 PmABCB6
1023 PmABCB30
15 MdABCB9
221 PcABCB23
27 MdABCB21
224 PcABCB26
28 MdABCB22
225 PcABCB27
0.53169
0.0616
0.21433
0.31735
0.21433
0.32704
0.20151
0.1499
0.17714
0.1499
0.119
0.06496
0.38148
0.06474
0.02577
0.01967
0.06547
0.04872
0.30384
0.0312
0.23048
0.07336
0.23048
0.07959
0.17688
0.15816
0.12796
0.04892
0.06315
0.0652
0.06481
0.06315
0.01998
0.30782
0.17286
0.10681
0.13496
0.13994
0.03364
0.06793
0.03291
0.03364
0.03837
0.06694
0.02866
0.59636
0.3088
0.24061
0.15032
0.0572
0.06573
0.0572
0.03834
0.06818
0.06576
0.05718
0.03765
0.03765
0.11592
0.07934
0.04551
0.09624
0.01953
0.05555
0.04279
0.05555
0.13724
0.04341
0.05452

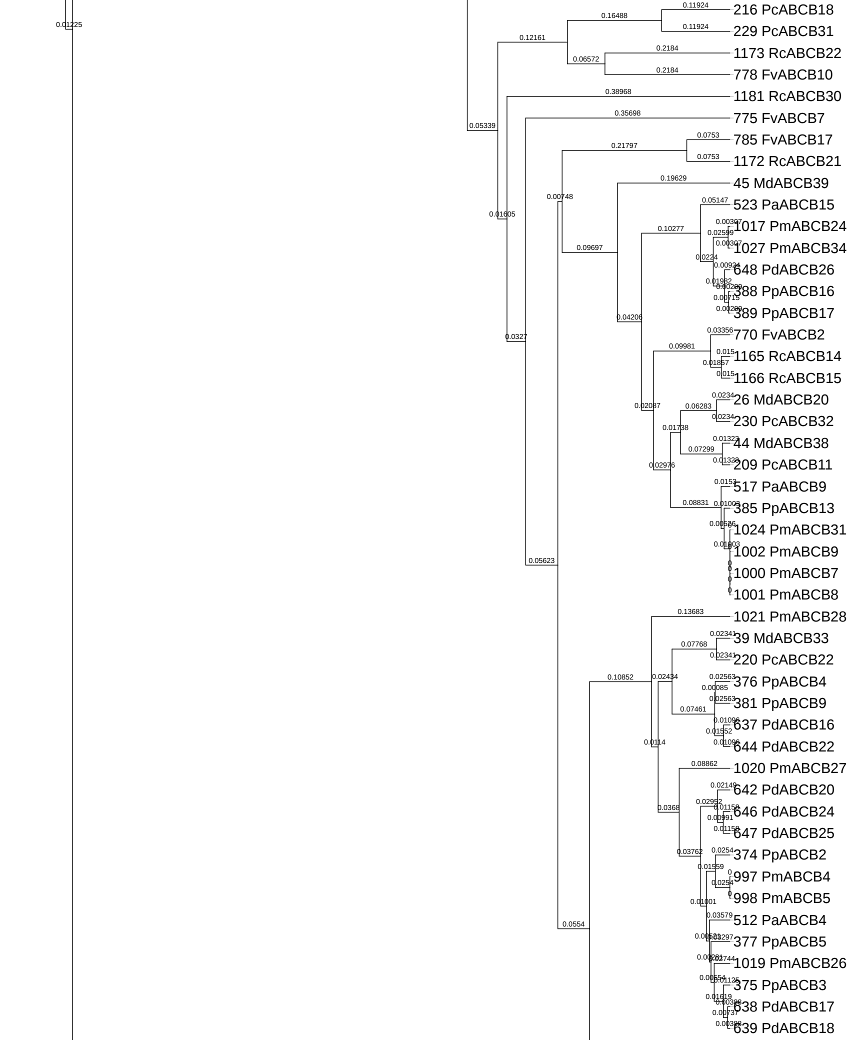

216 PcABCB18
229 PcABCB31
1173 RcABCB22
778 FvABCB10
1181 RcABCB30
775 FvABCB7
785 FvABCB17
1172 RcABCB21
45 MdABCB39
523 PaABCB15
1017 PmABCB24
1027 PmABCB34
648 PdABCB26
388 PpABCB16
389 PpABCB17
770 FvABCB2
1165 RcABCB14
1166 RcABCB15
26 MdABCB20
230 PcABCB32
44 MdABCB38
209 PcABCB11
517 PaABCB9
385 PpABCB13
1024 PmABCB31
1002 PmABCB9
1000 PmABCB7
1001 PmABCB8
1021 PmABCB28
39 MdABCB33
220 PcABCB22
376 PpABCB4
381 PpABCB9
637 PdABCB16
644 PdABCB22
1020 PmABCB27
642 PdABCB20
646 PdABCB24
647 PdABCB25
374 PpABCB2
997 PmABCB4
998 PmABCB5
512 PaABCB4
377 PpABCB5
1019 PmABCB26
375 PpABCB3
638 PdABCB17
639 PdABCB18

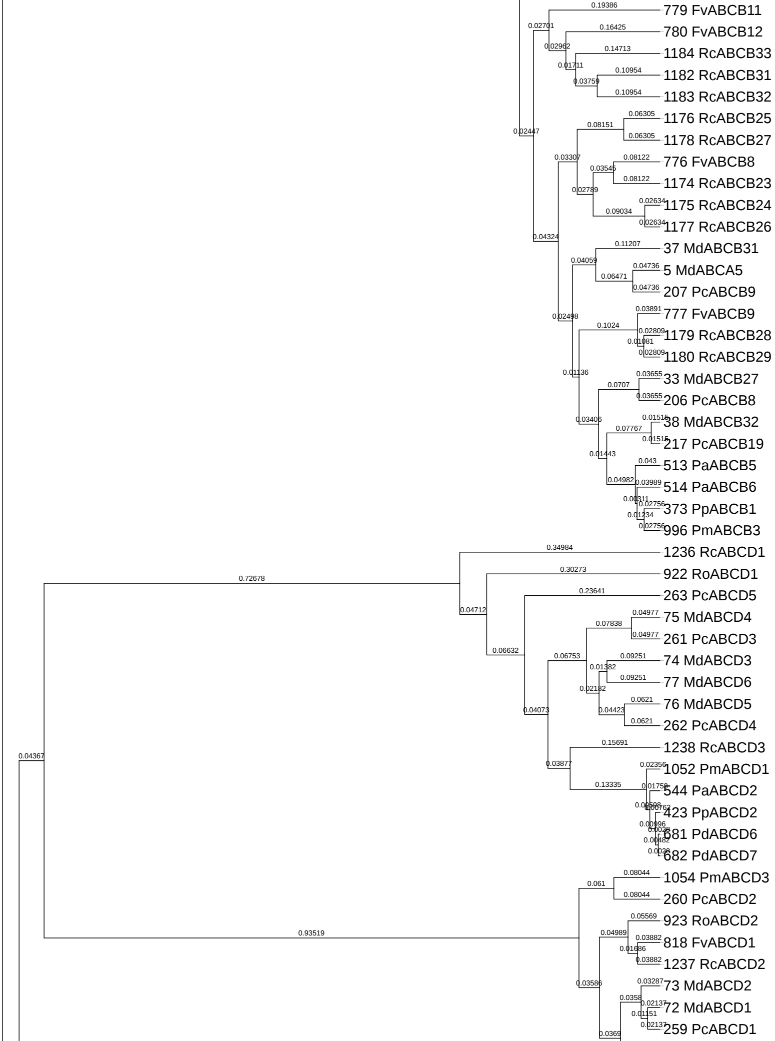
779 FvABCB11
780 FvABCB12
1184 RcABCB33
1182 RcABCB31
1183 RcABCB32
1176 RcABCB25
1178 RcABCB27
776 FvABCB8
1174 RcABCB23
1175 RcABCB24
1177 RcABCB26
37 MdABCB31
5 MdABCA5
207 PcABCB9
777 FvABCB9
1179 RcABCB28
1180 RcABCB29
33 MdABCB27
206 PcABCB8
38 MdABCB32
217 PcABCB19
513 PaABCB5
514 PaABCB6
373 PpABCB1
996 PmABCB3
1236 RcABCD1
922 RoABCD1
263 PcABCD5
75 MdABCD4
261 PcABCD3
74 MdABCD3
77 MdABCD6
76 MdABCD5
262 PcABCD4
1238 RcABCD3
1052 PmABCD1
544 PaABCD2
423 PpABCD2
681 PdABCD6
682 PdABCD7
1054 PmABCD3
260 PcABCD2
923 RoABCD2
818 FvABCD1
1237 RcABCD2
73 MdABCD2
72 MdABCD1
259 PcABCD1
0.72678
0.93519
0.04367
0.34984
0.30273
0.23641

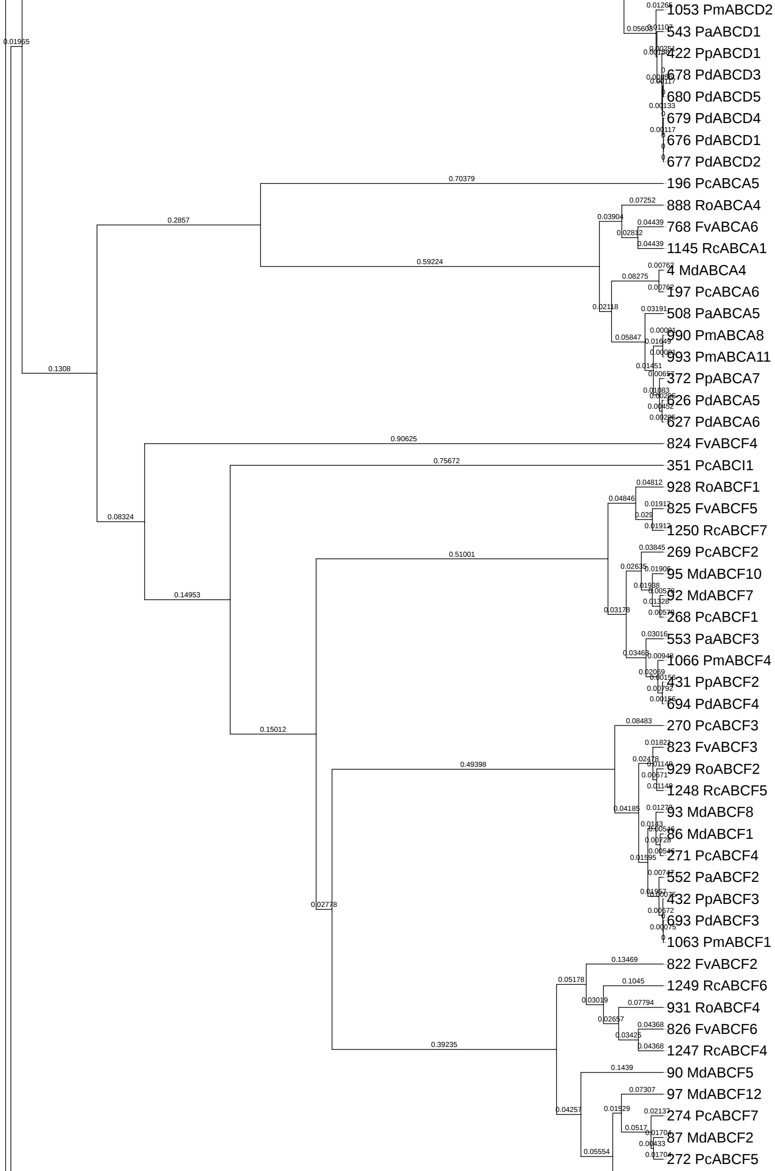

1053 PmABCD2
543 PaABCD1
422 PpABCD1
678 PdABCD3
680 PdABCD5
679 PdABCD4
676 PdABCD1
677 PdABCD2
196 PcABCA5
888 RoABCA4
768 FvABCA6
1145 RcABCA1
4 MdABCA4
197 PcABCA6
508 PaABCA5
990 PmABCA8
993 PmABCA11
372 PpABCA7
626 PdABCA5
627 PdABCA6
824 FvABCF4
351 PcABCI1
928 RoABCF1
825 FvABCF5
1250 RcABCF7
269 PcABCF2
95 MdABCF10
92 MdABCF7
268 PcABCF1
553 PaABCF3
1066 PmABCF4
431 PpABCF2
694 PdABCF4
270 PcABCF3
823 FvABCF3
929 RoABCF2
1248 RcABCF5
93 MdABCF8
86 MdABCF1
271 PcABCF4
552 PaABCF2
432 PpABCF3
693 PdABCF3
1063 PmABCF1
822 FvABCF2
1249 RcABCF6
931 RoABCF4
826 FvABCF6
1247 RcABCF4
90 MdABCF5
97 MdABCF12
274 PcABCF7
87 MdABCF2
272 PcABCF5
0.01965
0.70379
0.2857
0.59224
0.1308
0.90625
0.75672
0.08324
0.51001
0.14953
0.49398
0.15012
0.02778
0.39235

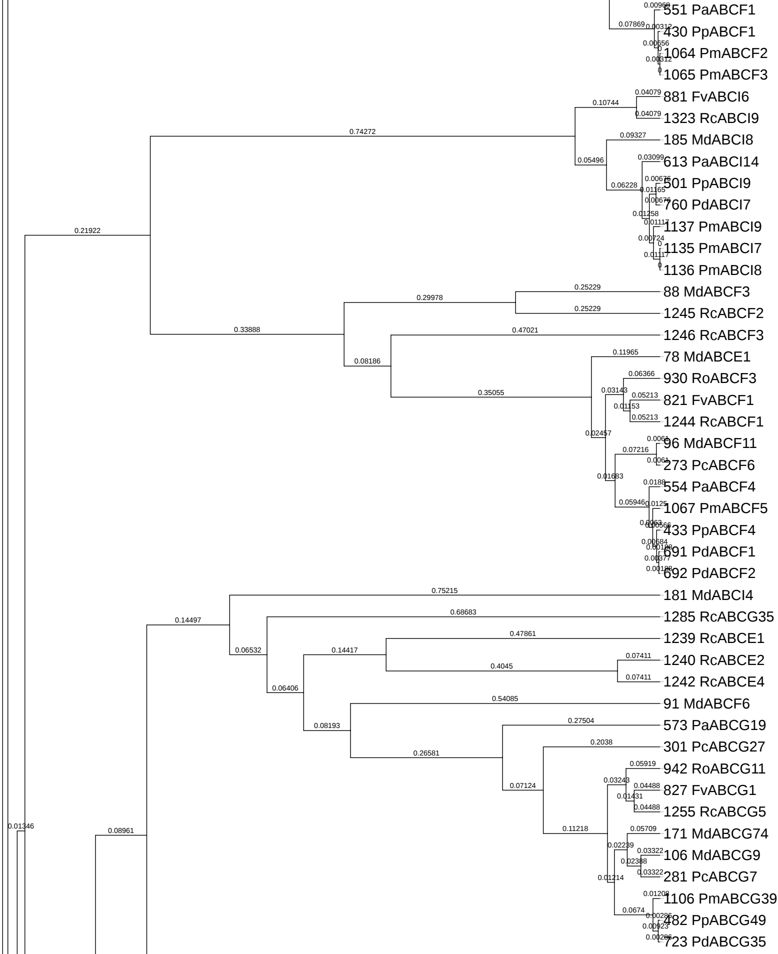
551 PaABCF1
430 PpABCF1
1064 PmABCF2
1065 PmABCF3
881 FvABCI6
1323 RcABCI9
185 MdABCI8
613 PaABCI14
501 PpABCI9
760 PdABCI7
1137 PmABCI9
1135 PmABCI7
1136 PmABCI8
88 MdABCF3
1245 RcABCF2
1246 RcABCF3
78 MdABCE1
930 RoABCF3
821 FvABCF1
1244 RcABCF1
96 MdABCF11
273 PcABCF6
554 PaABCF4
1067 PmABCF5
433 PpABCF4
691 PdABCF1
692 PdABCF2
181 MdABCI4
1285 RcABCG35
1239 RcABCE1
1240 RcABCE2
1242 RcABCE4
91 MdABCF6
573 PaABCG19
301 PcABCG27
942 RoABCG11
827 FvABCG1
1255 RcABCG5
171 MdABCG74
106 MdABCG9
281 PcABCG7
1106 PmABCG39
482 PpABCG49
723 PdABCG35

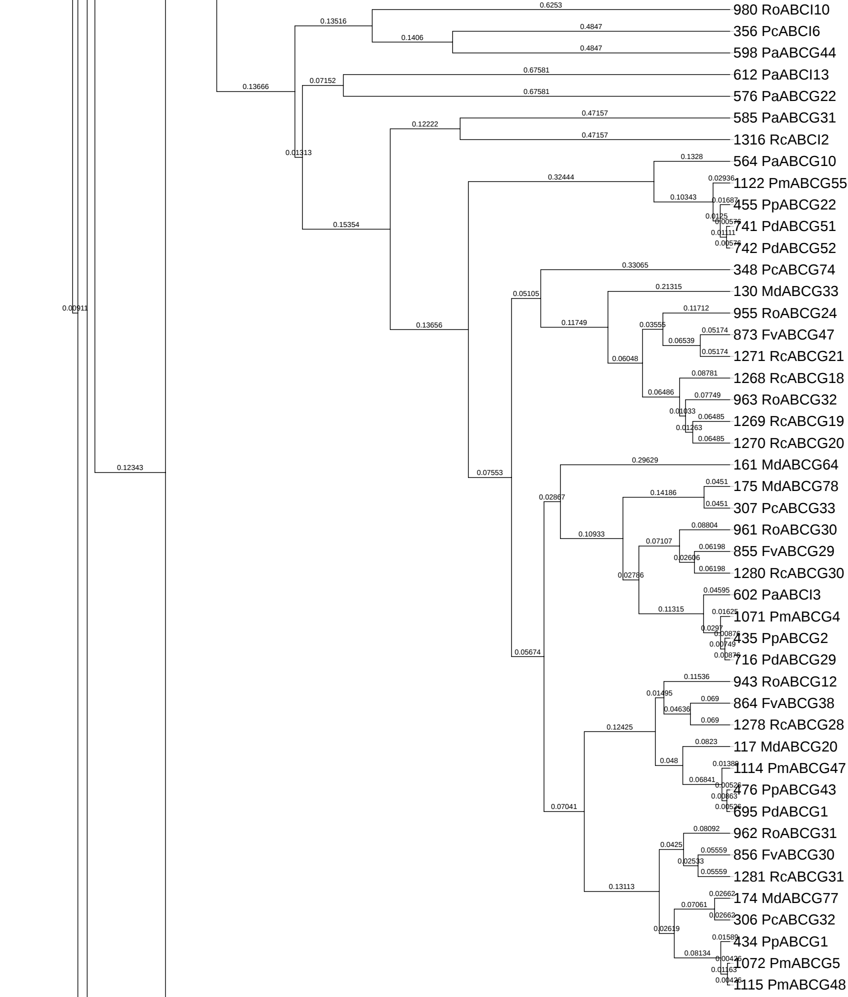
980 RoABCI10
356 PcABCI6
598 PaABCG44
612 PaABCI13
576 PaABCG22
585 PaABCG31
1316 RcABCI2
564 PaABCG10
1122 PmABCG55
455 PpABCG22
741 PdABCG51
742 PdABCG52
348 PcABCG74
130 MdABCG33
955 RoABCG24
873 FvABCG47
1271 RcABCG21
1268 RcABCG18
963 RoABCG32
1269 RcABCG19
1270 RcABCG20
161 MdABCG64
175 MdABCG78
307 PcABCG33
961 RoABCG30
855 FvABCG29
1280 RcABCG30
602 PaABCI3
1071 PmABCG4
435 PpABCG2
716 PdABCG29
943 RoABCG12
864 FvABCG38
1278 RcABCG28
117 MdABCG20
1114 PmABCG47
476 PpABCG43
695 PdABCG1
962 RoABCG31
856 FvABCG30
1281 RcABCG31
174 MdABCG77
306 PcABCG32
434 PpABCG1
1072 PmABCG5
1115 PmABCG48

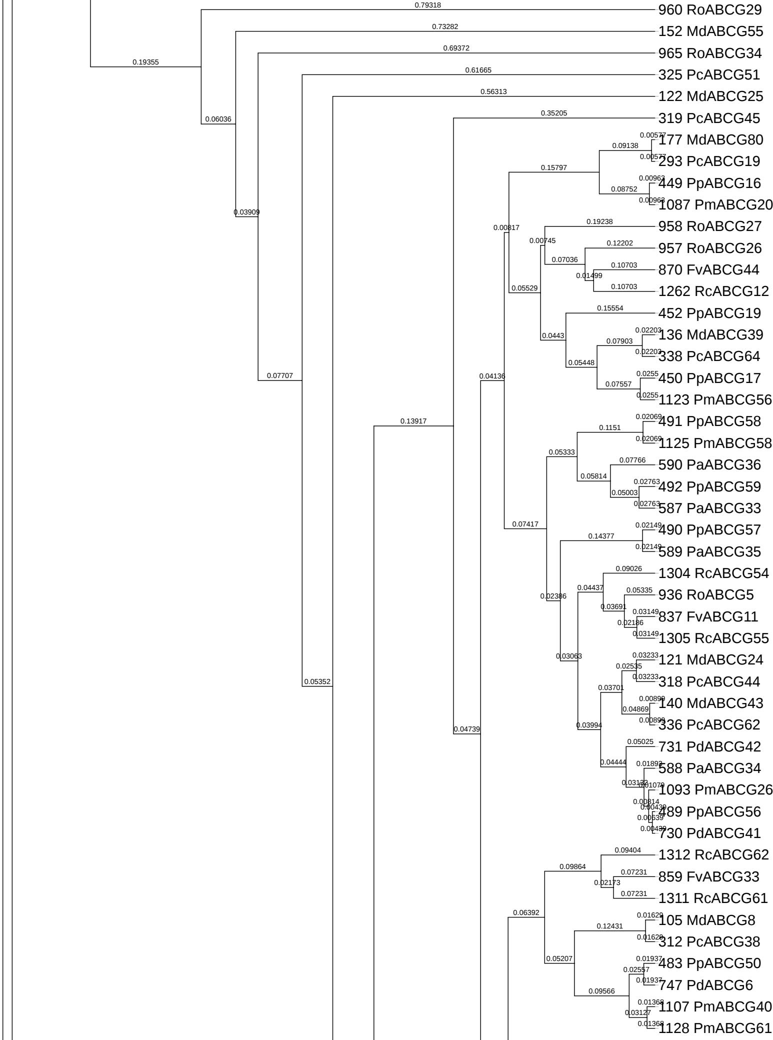
960 RoABCG29
152 MdABCG55
965 RoABCG34
325 PcABCG51
122 MdABCG25
319 PcABCG45
177 MdABCG80
293 PcABCG19
449 PpABCG16
1087 PmABCG20
958 RoABCG27
957 RoABCG26
870 FvABCG44
1262 RcABCG12
452 PpABCG19
136 MdABCG39
338 PcABCG64
450 PpABCG17
1123 PmABCG56
491 PpABCG58
1125 PmABCG58
590 PaABCG36
492 PpABCG59
587 PaABCG33
490 PpABCG57
589 PaABCG35
1304 RcABCG54
936 RoABCG5
837 FvABCG11
1305 RcABCG55
121 MdABCG24
318 PcABCG44
140 MdABCG43
336 PcABCG62
731 PdABCG42
588 PaABCG34
1093 PmABCG26
489 PpABCG56
730 PdABCG41
1312 RcABCG62
859 FvABCG33
1311 RcABCG61
105 MdABCG8
312 PcABCG38
483 PpABCG50
747 PdABCG6
1107 PmABCG40
1128 PmABCG61

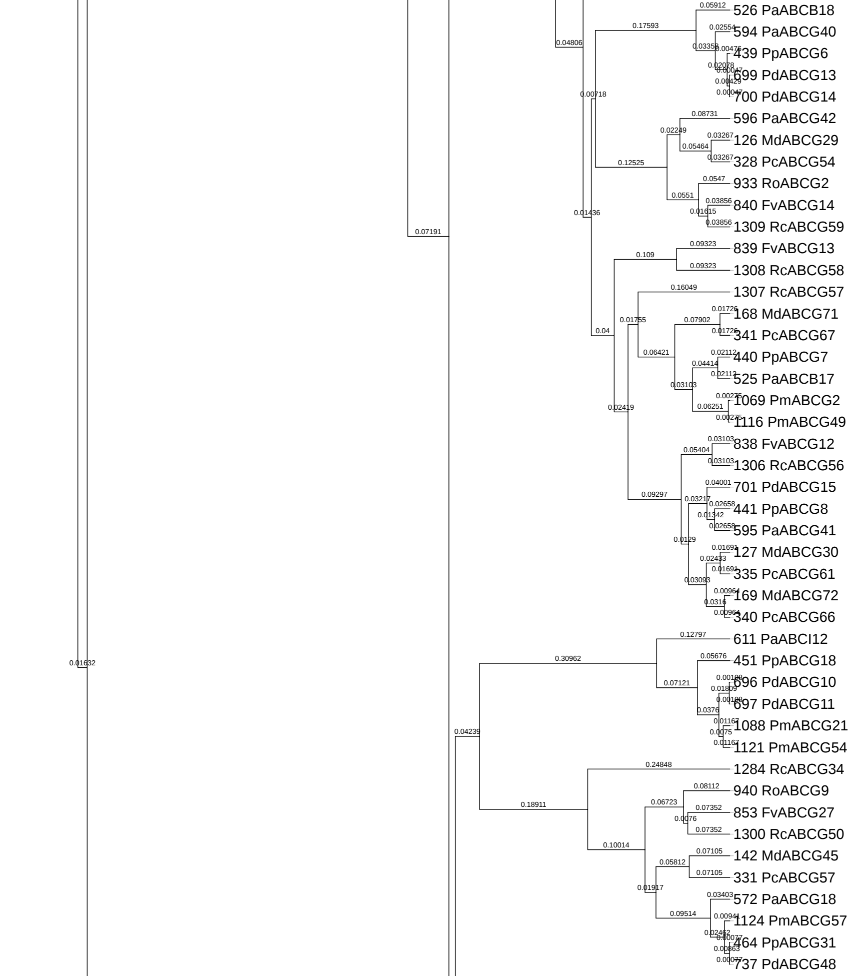
526 PaABCB18
594 PaABCG40
439 PpABCG6
699 PdABCG13
700 PdABCG14
596 PaABCG42
126 MdABCG29
328 PcABCG54
933 RoABCG2
840 FvABCG14
1309 RcABCG59
839 FvABCG13
1308 RcABCG58
1307 RcABCG57
168 MdABCG71
341 PcABCG67
440 PpABCG7
525 PaABCB17
1069 PmABCG2
1116 PmABCG49
838 FvABCG12
1306 RcABCG56
701 PdABCG15
441 PpABCG8
595 PaABCG41
127 MdABCG30
335 PcABCG61
169 MdABCG72
340 PcABCG66
611 PaABCI12
451 PpABCG18
696 PdABCG10
697 PdABCG11
1088 PmABCG21
1121 PmABCG54
1284 RcABCG34
940 RoABCG9
853 FvABCG27
1300 RcABCG50
142 MdABCG45
331 PcABCG57
572 PaABCG18
1124 PmABCG57
464 PpABCG31
737 PdABCG48

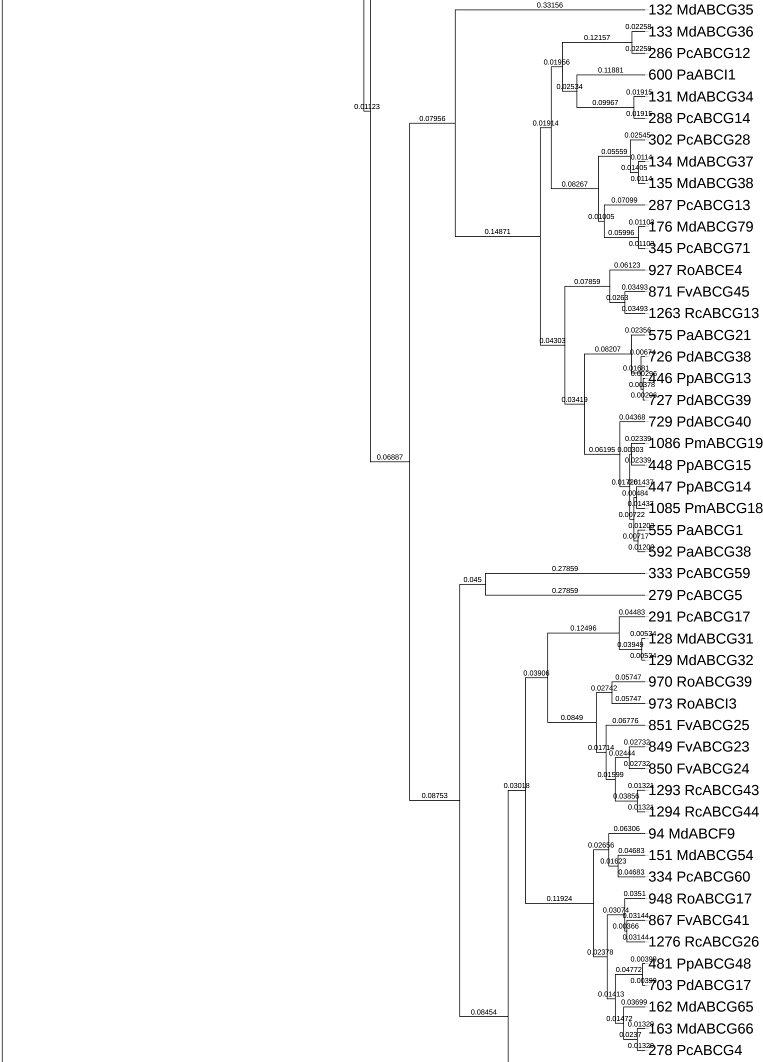
132 MdABCG35
133 MdABCG36
286 PcABCG12
600 PaABCI1
131 MdABCG34
288 PcABCG14
302 PcABCG28
134 MdABCG37
135 MdABCG38
287 PcABCG13
176 MdABCG79
345 PcABCG71
927 RoABCE4
871 FvABCG45
1263 RcABCG13
575 PaABCG21
726 PdABCG38
446 PpABCG13
727 PdABCG39
729 PdABCG40
1086 PmABCG19
448 PpABCG15
447 PpABCG14
1085 PmABCG18
555 PaABCG1
592 PaABCG38
333 PcABCG59
279 PcABCG5
291 PcABCG17
128 MdABCG31
129 MdABCG32
970 RoABCG39
973 RoABCI3
851 FvABCG25
849 FvABCG23
850 FvABCG24
1293 RcABCG43
1294 RcABCG44
94 MdABCF9
151 MdABCG54
334 PcABCG60
948 RoABCG17
867 FvABCG41
1276 RcABCG26
481 PpABCG48
703 PdABCG17
162 MdABCG65
163 MdABCG66
278 PcABCG4

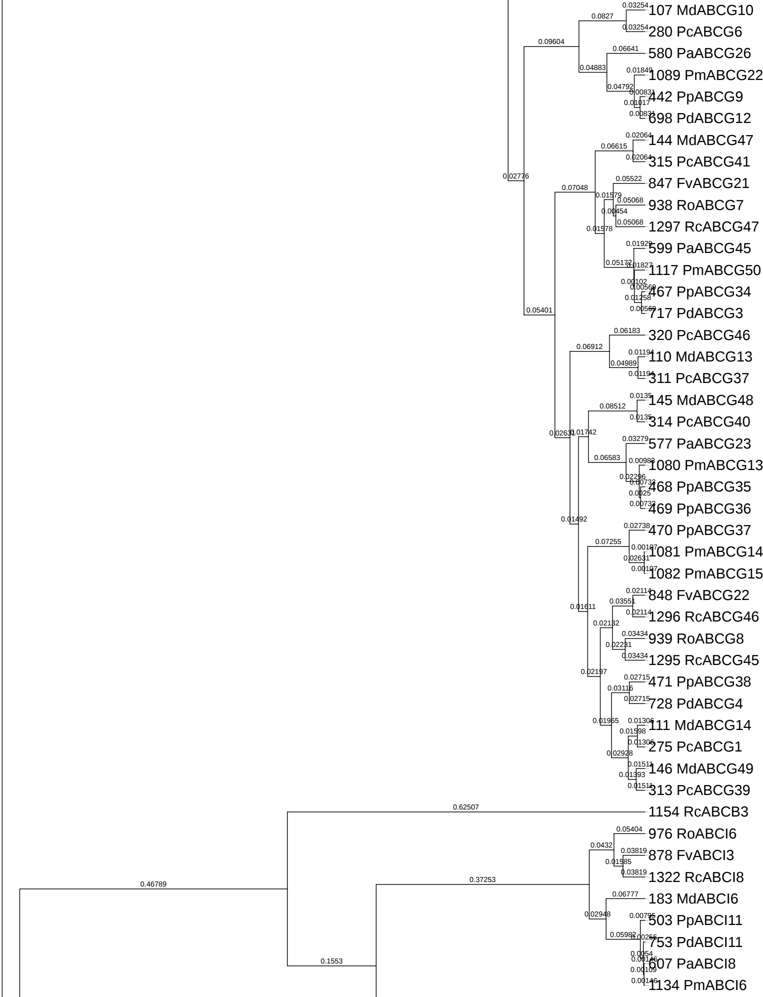
107 MdABCG10
280 PcABCG6
580 PaABCG26
1089 PmABCG22
442 PpABCG9
698 PdABCG12
144 MdABCG47
315 PcABCG41
847 FvABCG21
938 RoABCG7
1297 RcABCG47
599 PaABCG45
1117 PmABCG50
467 PpABCG34
717 PdABCG3
320 PcABCG46
110 MdABCG13
311 PcABCG37
145 MdABCG48
314 PcABCG40
577 PaABCG23
1080 PmABCG13
468 PpABCG35
469 PpABCG36
470 PpABCG37
1081 PmABCG14
1082 PmABCG15
848 FvABCG22
1296 RcABCG46
939 RoABCG8
1295 RcABCG45
471 PpABCG38
728 PdABCG4
111 MdABCG14
275 PcABCG1
146 MdABCG49
313 PcABCG39
1154 RcABCB3
976 RoABCI6
878 FvABCI3
1322 RcABCI8
183 MdABCI6
503 PpABCI11
753 PdABCI11
607 PaABCI8
1134 PmABCI6

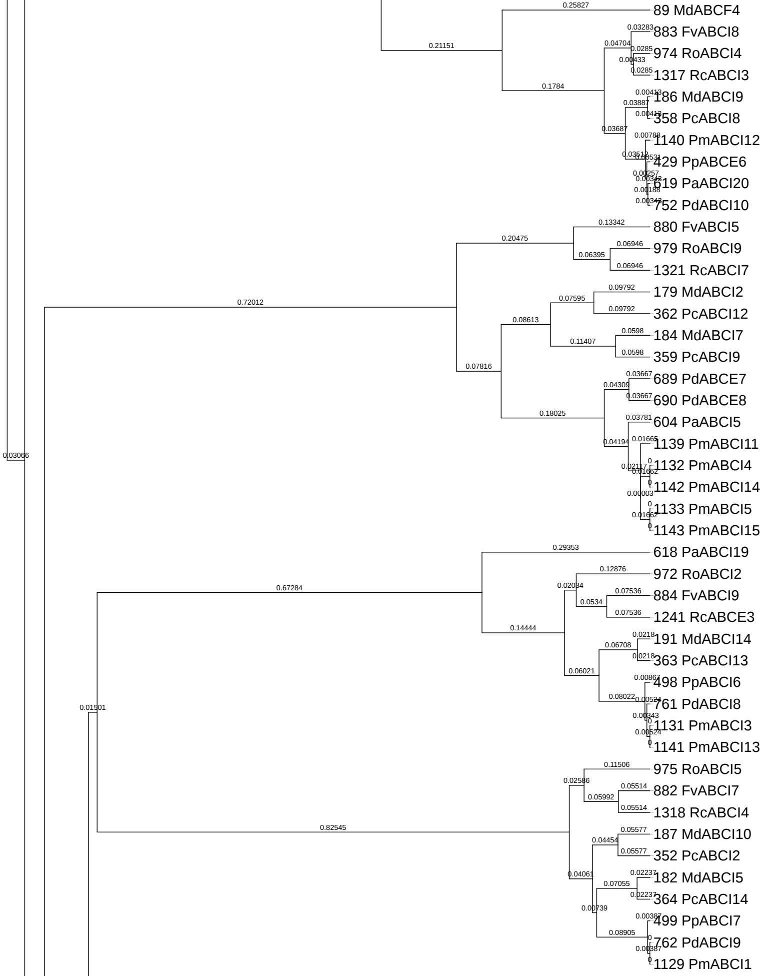
89 MdABCF4
883 FvABCI8
974 RoABCI4
1317 RcABCI3
186 MdABCI9
358 PcABCI8
1140 PmABCI12
429 PpABCE6
619 PaABCI20
752 PdABCI10
880 FvABCI5
979 RoABCI9
1321 RcABCI7
179 MdABCI2
362 PcABCI12
184 MdABCI7
359 PcABCI9
689 PdABCE7
690 PdABCE8
604 PaABCI5
1139 PmABCI11
1132 PmABCI4
1142 PmABCI14
1133 PmABCI5
1143 PmABCI15
618 PaABCI19
972 RoABCI2
884 FvABCI9
1241 RcABCE3
191 MdABCI14
363 PcABCI13
498 PpABCI6
761 PdABCI8
1131 PmABCI3
1141 PmABCI13
975 RoABCI5
882 FvABCI7
1318 RcABCI4
187 MdABCI10
352 PcABCI2
182 MdABCI5
364 PcABCI14
499 PpABCI7
762 PdABCI9
1129 PmABCI1

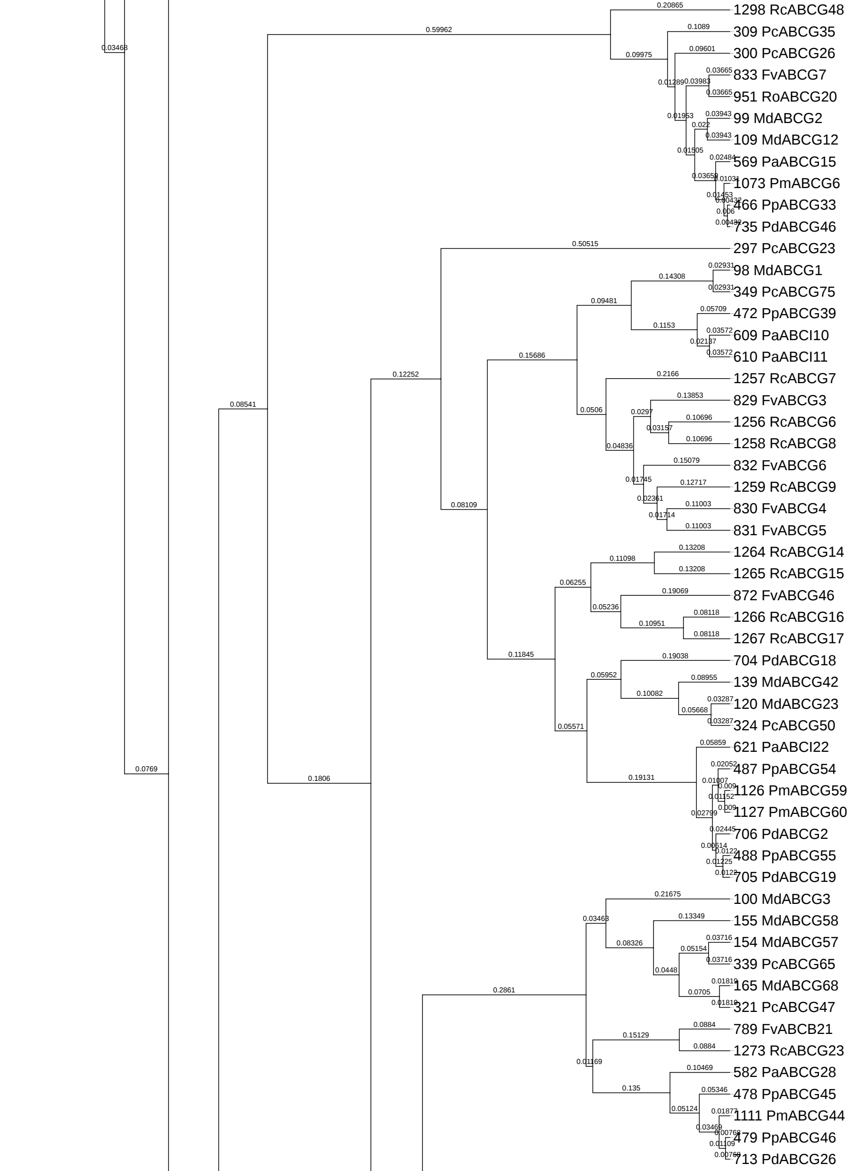
1298 RcABCG48
309 PcABCG35
300 PcABCG26
833 FvABCG7
951 RoABCG20
99 MdABCG2
109 MdABCG12
569 PaABCG15
1073 PmABCG6
466 PpABCG33
735 PdABCG46
297 PcABCG23
98 MdABCG1
349 PcABCG75
472 PpABCG39
609 PaABCI10
610 PaABCI11
1257 RcABCG7
829 FvABCG3
1256 RcABCG6
1258 RcABCG8
832 FvABCG6
1259 RcABCG9
830 FvABCG4
831 FvABCG5
1264 RcABCG14
1265 RcABCG15
872 FvABCG46
1266 RcABCG16
1267 RcABCG17
704 PdABCG18
139 MdABCG42
120 MdABCG23
324 PcABCG50
621 PaABCI22
487 PpABCG54
1126 PmABCG59
1127 PmABCG60
706 PdABCG2
488 PpABCG55
705 PdABCG19
100 MdABCG3
155 MdABCG58
154 MdABCG57
339 PcABCG65
165 MdABCG68
321 PcABCG47
789 FvABCB21
1273 RcABCG23
582 PaABCG28
478 PpABCG45
1111 PmABCG44
479 PpABCG46
713 PdABCG26

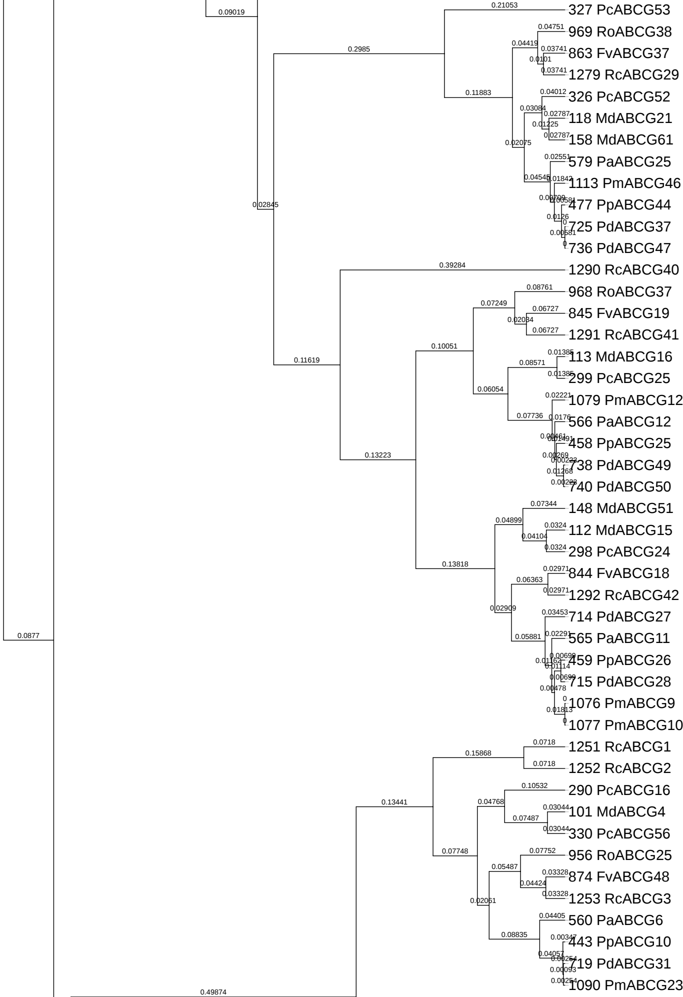
327 PcABCG53
969 RoABCG38
863 FvABCG37
1279 RcABCG29
326 PcABCG52
118 MdABCG21
158 MdABCG61
579 PaABCG25
1113 PmABCG46
477 PpABCG44
725 PdABCG37
736 PdABCG47
1290 RcABCG40
968 RoABCG37
845 FvABCG19
1291 RcABCG41
113 MdABCG16
299 PcABCG25
1079 PmABCG12
566 PaABCG12
458 PpABCG25
738 PdABCG49
740 PdABCG50
148 MdABCG51
112 MdABCG15
298 PcABCG24
844 FvABCG18
1292 RcABCG42
714 PdABCG27
565 PaABCG11
459 PpABCG26
715 PdABCG28
1076 PmABCG9
1077 PmABCG10
1251 RcABCG1
1252 RcABCG2
290 PcABCG16
101 MdABCG4
330 PcABCG56
956 RoABCG25
874 FvABCG48
1253 RcABCG3
560 PaABCG6
443 PpABCG10
719 PdABCG31
1090 PmABCG23

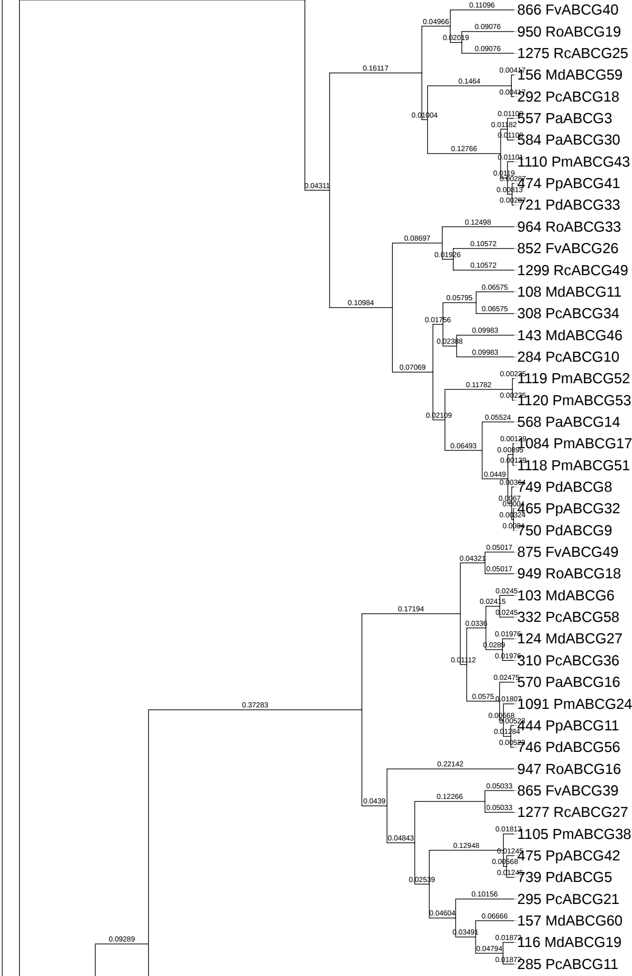
866 FvABCG40
950 RoABCG19
1275 RcABCG25
156 MdABCG59
292 PcABCG18
557 PaABCG3
584 PaABCG30
1110 PmABCG43
474 PpABCG41
721 PdABCG33
964 RoABCG33
852 FvABCG26
1299 RcABCG49
108 MdABCG11
308 PcABCG34
143 MdABCG46
284 PcABCG10
1119 PmABCG52
1120 PmABCG53
568 PaABCG14
1084 PmABCG17
1118 PmABCG51
749 PdABCG8
465 PpABCG32
750 PdABCG9
875 FvABCG49
949 RoABCG18
103 MdABCG6
332 PcABCG58
124 MdABCG27
310 PcABCG36
570 PaABCG16
1091 PmABCG24
444 PpABCG11
746 PdABCG56
947 RoABCG16
865 FvABCG39
1277 RcABCG27
1105 PmABCG38
475 PpABCG42
739 PdABCG5
295 PcABCG21
157 MdABCG60
116 MdABCG19
285 PcABCG11

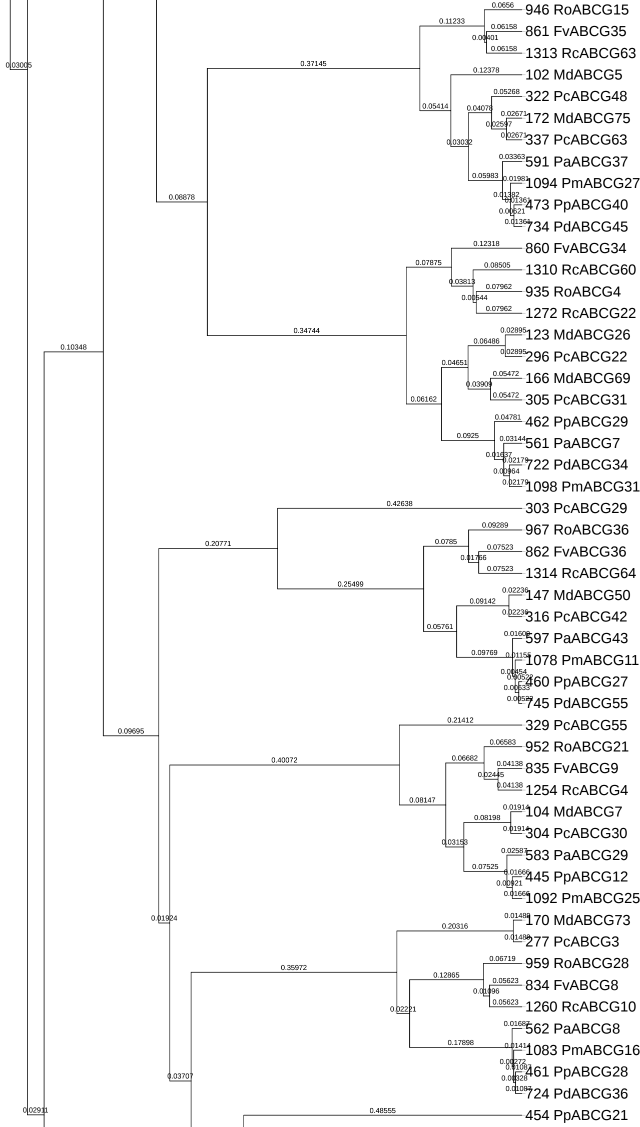
946 RoABCG15
861 FvABCG35
1313 RcABCG63
102 MdABCG5
322 PcABCG48
172 MdABCG75
337 PcABCG63
591 PaABCG37
1094 PmABCG27
473 PpABCG40
734 PdABCG45
860 FvABCG34
1310 RcABCG60
935 RoABCG4
1272 RcABCG22
123 MdABCG26
296 PcABCG22
166 MdABCG69
305 PcABCG31
462 PpABCG29
561 PaABCG7
722 PdABCG34
1098 PmABCG31
303 PcABCG29
967 RoABCG36
862 FvABCG36
1314 RcABCG64
147 MdABCG50
316 PcABCG42
597 PaABCG43
1078 PmABCG11
460 PpABCG27
745 PdABCG55
329 PcABCG55
952 RoABCG21
835 FvABCG9
1254 RcABCG4
104 MdABCG7
304 PcABCG30
583 PaABCG29
445 PpABCG12
1092 PmABCG25
170 MdABCG73
277 PcABCG3
959 RoABCG28
834 FvABCG8
1260 RcABCG10
562 PaABCG8
1083 PmABCG16
461 PpABCG28
724 PdABCG36
454 PpABCG21

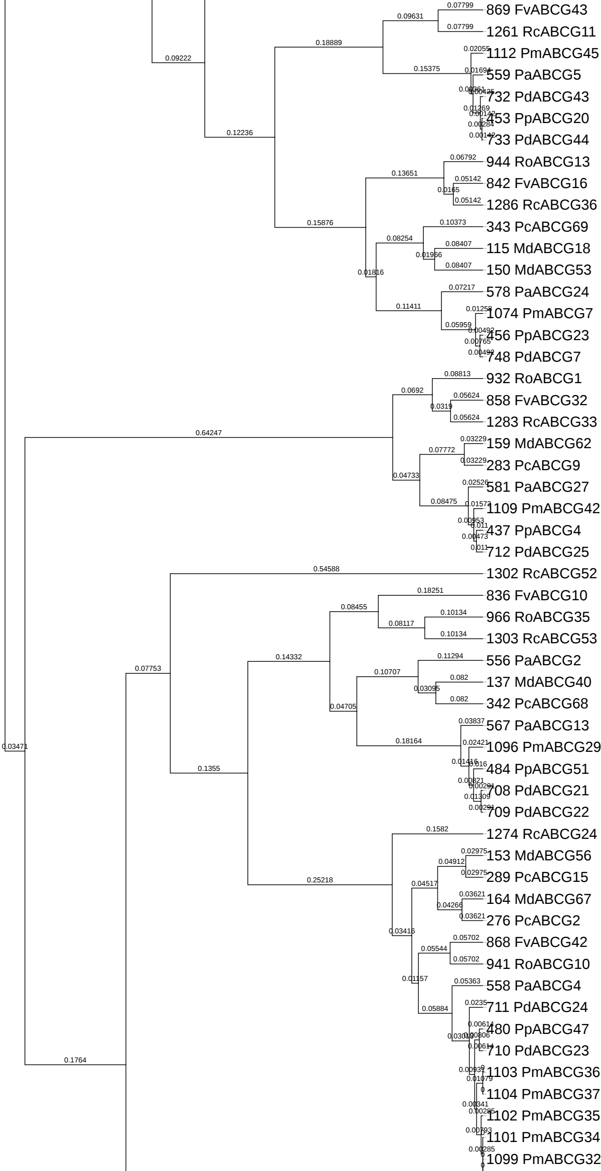
869 FvABCG43
1261 RcABCG11
1112 PmABCG45
559 PaABCG5
732 PdABCG43
453 PpABCG20
733 PdABCG44
944 RoABCG13
842 FvABCG16
1286 RcABCG36
343 PcABCG69
115 MdABCG18
150 MdABCG53
578 PaABCG24
1074 PmABCG7
456 PpABCG23
748 PdABCG7
932 RoABCG1
858 FvABCG32
1283 RcABCG33
159 MdABCG62
283 PcABCG9
581 PaABCG27
1109 PmABCG42
437 PpABCG4
712 PdABCG25
1302 RcABCG52
836 FvABCG10
966 RoABCG35
1303 RcABCG53
556 PaABCG2
137 MdABCG40
342 PcABCG68
567 PaABCG13
1096 PmABCG29
484 PpABCG51
708 PdABCG21
709 PdABCG22
1274 RcABCG24
153 MdABCG56
289 PcABCG15
164 MdABCG67
276 PcABCG2
868 FvABCG42
941 RoABCG10
558 PaABCG4
711 PdABCG24
480 PpABCG47
710 PdABCG23
1103 PmABCG36
1104 PmABCG37
1102 PmABCG35
1101 PmABCG34
1099 PmABCG32
0.07799
0.09631
0.18889
0.15375
0.09222
0.12236
0.06792
0.13651
0.05142
0.15876
0.10373
0.08254
0.08407
0.01816
0.07217
0.11411
0.05959
0.08813
0.0692
0.05624
0.64247
0.03229
0.07772
0.04733
0.08475
0.54588
0.18251
0.08455
0.10134
0.08117
0.14332
0.11294
0.10707
0.082
0.07753
0.04705
0.18164
0.03471
0.1355
0.1582
0.04912
0.25218
0.04517
0.03416
0.05702
0.05544
0.05363
0.05884
0.1764

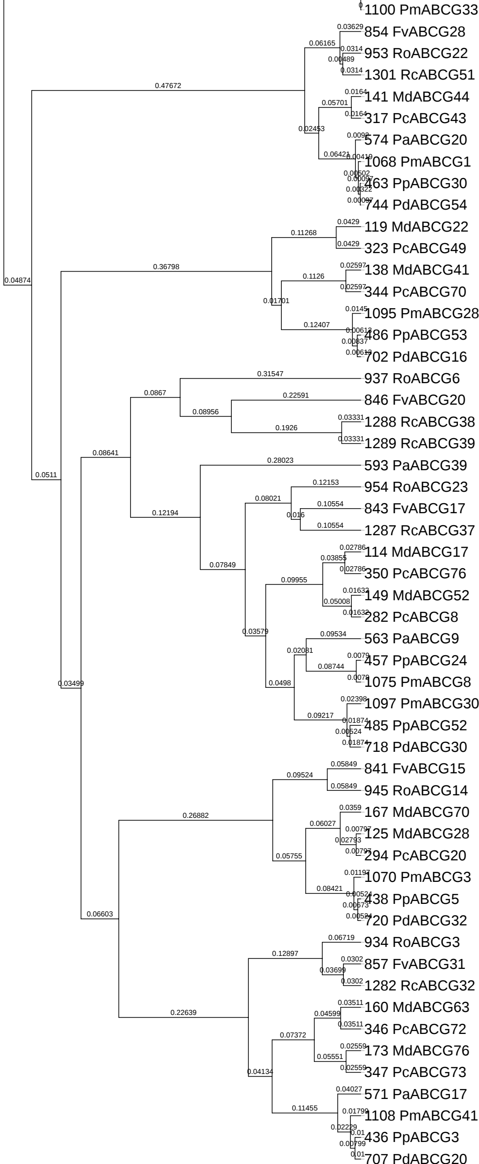
1100 PmABCG33
854 FvABCG28
953 RoABCG22
1301 RcABCG51
141 MdABCG44
317 PcABCG43
574 PaABCG20
1068 PmABCG1
463 PpABCG30
744 PdABCG54
119 MdABCG22
323 PcABCG49
138 MdABCG41
344 PcABCG70
1095 PmABCG28
486 PpABCG53
702 PdABCG16
937 RoABCG6
846 FvABCG20
1288 RcABCG38
1289 RcABCG39
593 PaABCG39
954 RoABCG23
843 FvABCG17
1287 RcABCG37
114 MdABCG17
350 PcABCG76
149 MdABCG52
282 PcABCG8
563 PaABCG9
457 PpABCG24
1075 PmABCG8
1097 PmABCG30
485 PpABCG52
718 PdABCG30
841 FvABCG15
945 RoABCG14
167 MdABCG70
125 MdABCG28
294 PcABCG20
1070 PmABCG3
438 PpABCG5
720 PdABCG32
934 RoABCG3
857 FvABCG31
1282 RcABCG32
160 MdABCG63
346 PcABCG72
173 MdABCG76
347 PcABCG73
571 PaABCG17
1108 PmABCG41
436 PpABCG3
707 PdABCG20
